# Supplementary material for: Constructing high-efficiency orange-red thermally activated delayed fluorescence emitters by three-dimension molecular engineering
Source: Nat Commun. 2022 Dec 19;13:7828. doi: 10.1038/s41467-022-35591-w (PMC9763412; doi:10.1038/s41467-022-35591-w)
Supplement: Supplementary file 1 — Supplementary Information [file 41467_2022_35591_MOESM1_ESM.pdf]

Supplementary information

Constructing High-Efficiency Orange-Red Thermally Activated Delayed Fluorescence Emitters by Three-Dimension Molecular Engineering

Lei Hua[1,2], Yuchao Liu[3], Binbin Liu[2], Zhennan Zhao[1,2], Lei Zhang[2], Shouke Yan[1,3], Zhongjie Ren[1,2]*

[1] State Key Laboratory of Chemical Resource Engineering, Beijing University of Chemical Technology, Beijing, 100029, China.
[2] Beijing Advanced Innovation Center for Soft Matter Science and Engineering, Beijing University of Chemical Technology, Beijing, 100029, China.
[3] Key Laboratory of Rubber-Plastics, Ministry of Education, Qingdao University of Science & Technology, Qingdao, 266042, China.

Table of contents

Supplementary Note 1: Methods…………………………………………..…………… S2

Supplementary Note 2: Materials synthesis………………………..………………… S5

Supplementary Note 3: NMR, HPLC and HRMS spectra…………………….……..... S14

Supplementary Note 4: Supplementary figures…………………………………..….... S39

Supplementary Note 5: Supplementary tables……….…………………………..…... S46

Supplementary References…………………………………………………...…...…… S47

Supplementary Note 1: Methods

S 1.1 Characterization

The nuclear magnetic resonance (NMR) spectra were obtained using a Bruker AVANCE III 400 spectrometer (400 MHz), a Bruker AVANCE III 300 spectrometer (300 MHz) or a Bruker AVANCE III 700 spectrometer (700 MHz). ^{1}H NMR and ^{13}C NMR were measured with TMS as internal standard. ^{1}H NMR spectra data are reported as chemical shift, relative integral, multiplicity (s = singlet, d = doublet, m = multiplet), coupling constant (*J* in Hz) and assignment.

X-ray crystallographic data was made with Synchrotron Radiation (λ = 0.82653 Å). All calculations were performed using the SHELXL-97 and the Crystal Structure crystallographic software package.

Molecular masses were determined by a MALDI-FTICR-MS with anhydrous dichloromethane as the matrix.

Ultraviolet-visible (UV-Vis) absorption spectra were recorded on a Hitachi U-2910 spectrophotometer in air atmosphere.

Room and ultra-low temperature (77 K) and photoluminescence (PL) spectra were recorded on a Hitachi F-7000 fluorescence spectrophotometer. The energy gap (ΔE_{ST}) between the lowest singlet (S_1) and triplet excited states (T_1) were determined from the difference values of the onset positions of fluorescence and phosphorescence spectra.

Fluorescence decay of doped films (x wt% doped in 5 wt% poly(N-vinylcarbazole) (PVK) and 100-x wt% 9H-carbazole-3-carbonitrile (mCP-CN)) were recorded in vacuum on a FS-5 spectrometer from Edinburgh Instruments Limited with picosecond pulsed diode laser emitting at 300 nm. Temperature-dependent fluorescence decay from 77 K to 300 K were also measured with the support of Oxford variable temperature accessories.

The photoluminance quantum yields (PLQYs) of the blended films were measured on FS-5 with an integrating sphere (φ = 150 mm). The quantum efficiencies and rate constants were determined using the following equations according to the literatures[1]:

$$k_{PF} = \frac{\Phi_{PF}}{\tau_{PF}} \quad (S1)$$

$$k_{DF} = \frac{\Phi_{DF}}{\tau_{DF}} \quad (S2)$$

$$k_{ISC} = \frac{\Phi_{PF}}{\Phi_{DF} + \Phi_{PF}} k_{PF} \quad (S3)$$

$$k_{RISC} = \frac{k_{DF} k_{PF}}{k_{ISC}} \frac{\Phi_{DF}}{\Phi_{PF}} \quad (S4)$$

$$k_{\mathrm{PF}} = k_r^S + k_{nr}^S + k_{\mathrm{ISC}} \quad (S5)$$

$$k_r^S = \eta_{\mathrm{PF}} \cdot k_{\mathrm{PF}} \quad (S6)$$

$$k_{nr}^S = k_{\mathrm{PF}} - k_r^S - k_{\mathrm{ISC}} \quad (S7)$$

The PLQYs of the materials in vacuum can be converted by integrating the steady state spectrum, and the PLQY in air. The equation is as follows:

$$PLQY_{vac} = \frac{I_{vac}}{I_{air}} PLQY_{air} \quad (S8)$$

In which, I_{vac} and I_{air} is the integrate of the steady state spectrum in vacuum and air atmospheres, respectively.

Cyclic voltammetry (CV) was carried out in nitrogen-purged acetonitrile at room temperature with a CHI voltametric analyzer. Tetrabutylammonium hexafluorophosphate (TBAPF6 0.1 M) was used as the supporting electrolyte. The conventional three-electrode configuration consists of a glassy carbon working electrode, a platinum wire auxiliary electrode, and an Ag/$AgNO_3$ pseudo-reference electrode with ferrocenium-ferrocene (Fc^+/Fc) as the external standard. Cyclic voltammograms were obtained at scan rate of 100 mV s^{-1}. The onset potential was determined from the intersection of two tangents drawn at the rising and background current of the cyclic voltammogram. The highest occupied molecular orbital (HOMO) and lowest unoccupied molecular orbital (LUMO) energy levels were calculated according to the external reference ferroceneredox couple in acetonitrile by using the following formulas[2]:

$$E_{HOMO} = -\left(E_{(onset,ox\ vs\ Fc^+/Fc)} + 4.8\right) \quad (S9)$$

$$E_{LUMO} = -\left(E_{(onset,red\ vs\ Fc^+/Fc)} + 4.8\right) \quad (S10)$$

The morphologies of the blended films (2% emitters doped in 5% PVK and 93% mCP-CN) coated on the quartz substrate were measured using atomic force microscopy (Agilent Technologies 5500) under tapping mode.

Differential scanning calorimetry (DSC) was performed on a TA Q2000 differential scanning calorimeter at a heating rate of 10 ºC min^{-1} from 30 to 300 ºC under nitrogen atmosphere. Thermogravimetric analysis (TGA) was performed with a METTLER TOLEDO TGA/DSC 1/1100SF instrument at a heating rate of 20 ºC min^{-1} from 30 to 780 ºC under nitrogen atmosphere.

High performance liquid chromatograph completed by Shimadzu LC10Atvp high performance liquid chromatograph with C18 chromatographic columns. And the mobile phase is tetrahydrofuran and n-hexane with flow rate of 0.5 mL/min.

The angle-dependent p-polarized emission were measured by a fiber optical spectrometer with an automatic rotation stage. The emitting dipole orientation was determined by least square fitting of the measured angle-dependent p-polarized emission intensity with calculated results.

S 1.2 Molecular simulation

Density functional theory (DFT) calculation was performed by Gaussian 09 in the B3LYP mode with a 6-31G (d, p) basis set in the ground state. According to the optimized results, HOMO and LUMO levels can be obtained. The excited states energy levels and the energy properties in the excited states were determined using Gaussian 09 with a PBE1PBE/6-311G (d, p) basis in time dependent (TD) mode. To boost the calculation precision of the singlet and triplet energy levels, the number of calculated states was set to 10. The natural transition orbital (NTO) analysis was performed using Multiwfn 3.6 based on Gaussian output results[3, 4]. The distributions of hole and electron can be found from the NTO results and the dominate nature, charge transfer (CT) or local excited state (LE), can be found out by the overlap integral of main contribution orbits.

To analyzing the impact of natural flip quantitatively, the SOC matrix elements for these molecules were calculated using the ORCA 4.1.2 package with B3LYP/G TZVP method, and five states were considered in the analog computation.

S 1.3 Optical simulation

The optical simulation was performed to predict the maximum external quantum efficiency (EQE) of tBu-S-mCP as functions of PLQY under the assumption of no electrical loss. The device structure was used for the simulation under the assumption of perfect electrical balance. The recombination

zone was assumed to be located in the middle of the emission layer. The input parameters include refractive index value, extinction coefficient, thickness of each layer values, as well as photoluminescence spectrum of the emitting layer. The refractive indexes and extinction coefficient of emitting layer was measured by variable angle spectroscopic ellipsometry (VASE). The other refractive index values were obtained in the literature [5, 6].

The increased ratio of light out-coupling efficiency for the light-emitting layer with horizontal dipole orientation versus non-oriented one (66.7%) was calculated using finite difference time domain (FDTD) approach by Lumerical FDTD Solutions 8.7.3. An emission dipole with a horizontal orientation (parallel to the substrate) was placed at middle positions. All photons emitted from the dipole into the glass substrate was integrated in the half-space bounded by the reflecting Al cathode. To compare the relative outcoupling efficiency, Gaussian-oscillating dipole pulse with fixed photon number was set for monitoring the light intensity.

Supplementary Note 2: Materials synthesis

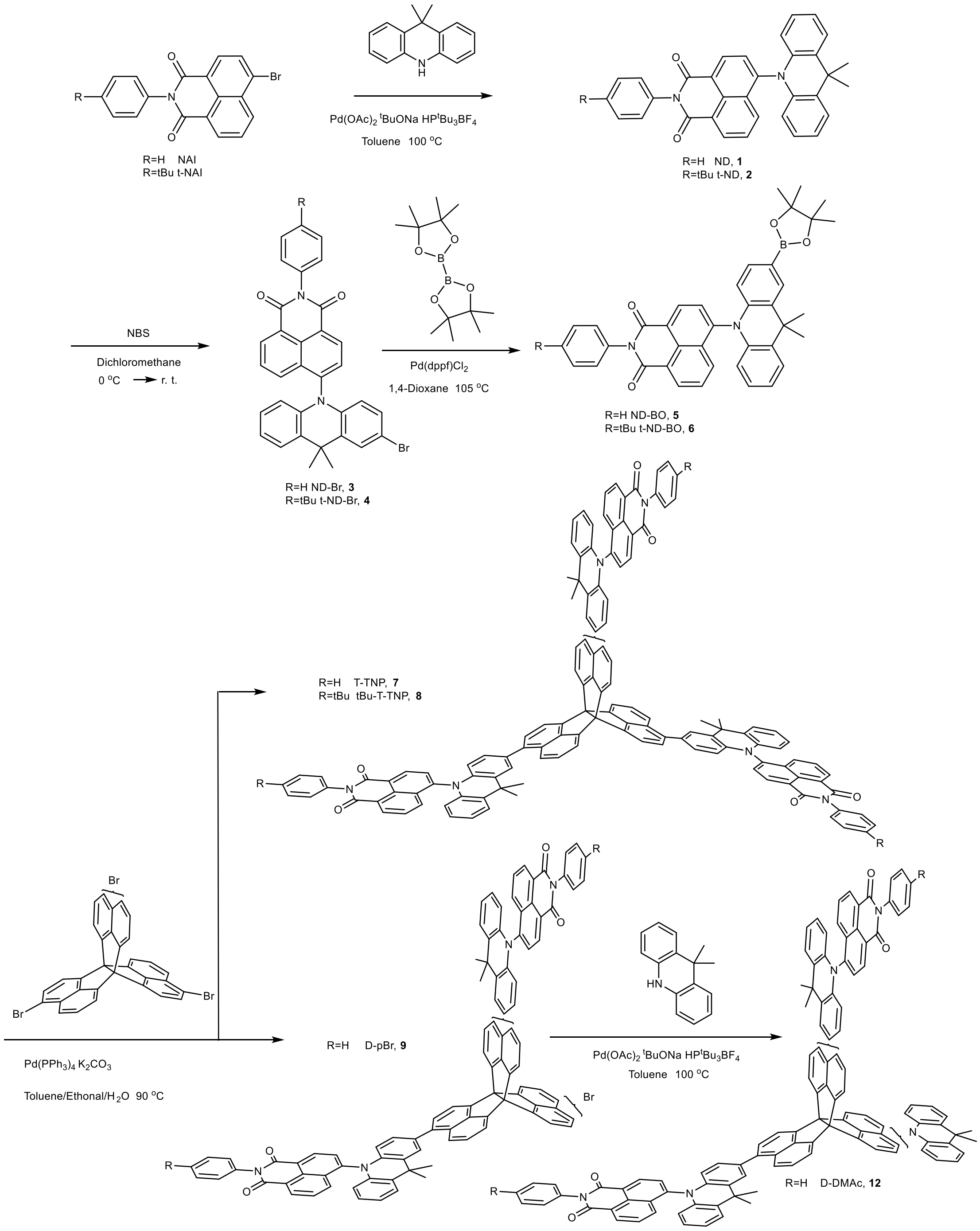

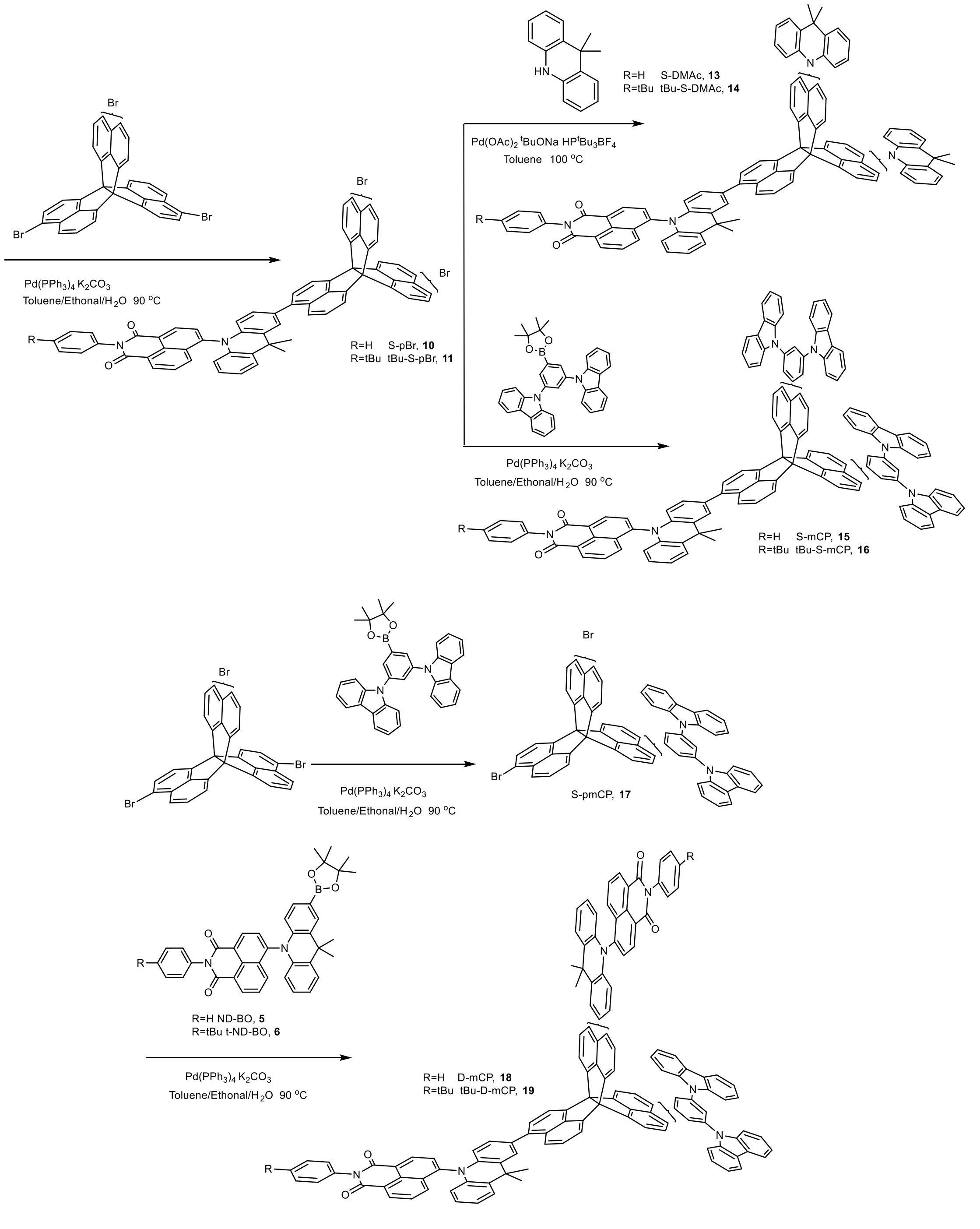

Supplementary Figure 1. Synthesis route of the three series emitters

Synthesis of ND (Compound 1)[7-9]

NAI (1000 mg, 2.8 mmol), 9,9-dimethyl-10H-acridine (627.9 mg, 3.0 mmol), palladium(II)acetate (22.4 mg, 0.1 mmol), tri-tert-butylphosphine tetrafluoroborate (86.2 mg, 0.3 mmol) and sodium tert-butoxide (537 mg, 5.6 mmol) were added to a 100 mL two-necked vial under an argon atmosphere. 40 mL toluene was injected sequentially as solvent. After stirring at 100 ºC for 24 h, the reaction was cooled down to room temperature. Then the mixture was washed three times by saturated salt water and extracted by dichloromethane. The obtained solution was dried with anhydrous magnesium sulfate, then filtered, and concentrated under reduced pressure. The crude product was further purified by column chromatography on silica gel using petroleum ether/ dichloromethane (v/v = 1/2) as eluent to obtain ND (**1**) as an orange

solid (1030 mg, 77% yield). ^{1}H NMR (400 MHz, Acetone-d_6) δ 8.84 (d, J = 7.6 Hz, 1H), 8.62 (dd, J = 7.2, 1.2 Hz, 1H), 8.06 (dd, J = 8.5, 1.2 Hz, 1H), 7.98 (d, J = 7.6 Hz, 1H), 7.83 (dd, J = 8.4, 7.2 Hz, 1H), 7.63 (dd, J = 7.7, 1.6 Hz, 2H), 7.61 – 7.55 (m, 2H), 7.54 – 7.46 (m, 3H), 6.96 (td, J = 7.5, 1.3 Hz, 2H), 6.89 (ddd, J = 8.1, 7.2, 1.6 Hz, 2H), 6.03 (dd, J = 8.1, 1.3 Hz, 2H), 1.81 (d, J = 39.1 Hz, 7H). ^{13}C NMR (75 MHz, Chloroform-d) δ 164.22, 163.88, 144.67, 140.22, 135.39, 133.12, 132.52, 130.97, 130.80, 130.64, 130.09, 129.62, 129.00, 128.75, 128.25, 126.88, 126.15, 124.02, 123.27, 121.46, 114.16, 36.21, 32.91, 32.26. MALDI-MS: m/z [M$^-$] calcd for $C_{33}H_{24}N_2O_2$ 480.1843 found 480.1841.

Synthesis of t-ND (Compound 2)

t-NAI (816.6 mg, 2.0 mmol), 9,9-dimethyl-10H-acridine (460.4 mg, 2.2 mmol), palladium(II)acetate (22.4 mg, 0.1 mmol), tri-tert-butylphosphine tetrafluoroborate (86.2 mg, 0.3 mmol) and sodium tert-butoxide (384.4 mg, 4 mmol) were added to a 100 mL two-necked vial under an argon atmosphere. 40 mL toluene was injected sequentially as solvent. After stirring at 100 ºC for 24 h, the reaction was cooled down to room temperature. Then the mixture was washed three times by saturated salt water and extracted by dichloromethane. The obtained solution was dried with anhydrous magnesium sulfate, then filtered, and concentrated under reduced pressure. The crude product was further purified by column chromatography on silica gel using petroleum ether/ dichloromethane (v/v = 1/1) as eluent to obtain t-ND (**2**) as an orange solid (860 mg, 80% yield). ^{1}H NMR (400 MHz, Chloroform-d) δ 8.87 (d, J = 7.6 Hz, 1H), 8.69 (dd, J = 7.2, 1.2 Hz, 1H), 8.10 (dd, J = 8.4, 1.2 Hz, 1H), 7.84 (d, J = 7.7 Hz, 1H), 7.67 (dd, J = 8.4, 7.2 Hz, 1H), 7.63 – 7.58 (m, 2H), 7.55 (dd, J = 7.8, 1.6 Hz, 2H), 7.29 (d, J = 8.5 Hz, 3H), 6.97 (td, J = 7.6, 1.3 Hz, 2H), 6.88 (ddd, J = 8.7, 7.2, 1.6 Hz, 2H), 5.99 (dd, J = 8.2, 1.2 Hz, 2H), 1.82 (d, J = 30.6 Hz, 6H), 1.40 (s, 9H). ^{13}C NMR (176 MHz, Chloroform-d) δ 164.30, 163.97, 151.70, 144.57, 140.22, 133.06, 132.55, 132.48, 130.98, 130.94, 130.78, 130.54, 130.08, 128.22, 128.06, 126.88, 126.65, 126.14, 124.09, 123.35, 121.45, 114.16, 36.20, 34.93, 32.87, 32.29, 31.53. MALDI-MS: m/z [M$^-$] calcd for $C_{37}H_{32}N_2O_2$ 536.2469 found 536.2466.

Synthesis of ND-Br (Compound 3)

ND (1 g, 2.1 mmol) was dissolved in 20 mL anhydrous dichloromethane at 0 ºC. The N-bromosuccinimide (407.39 mg, 2.29 mmol) dissolved in 20 mL anhydrous dichloromethane was dropped into reaction at 0 ºC with a constant pressure funnel in darkness. The reaction was quenched by water after stirring for overnight. Then the mixture was washed three times by water and extracted by dichloromethane. The obtained solution was dried with anhydrous magnesium sulfate, then filtered, and concentrated under reduced pressure. The crude product obtained was recrystallized with ethanol and acetone, then dried under reduced pressure to obtain ND-Br (**3**) as a pink solid (840 mg, 72% yield). ^{1}H NMR (400 MHz, DMSO-d_6) δ 8.74 (d, J = 7.7 Hz, 1H), 8.56 (dd, J = 7.1, 1.2 Hz, 1H), 8.00 (d, J = 7.7 Hz, 1H), 7.95 (dd, J = 8.4, 1.2 Hz, 1H), 7.85 (dd, J = 8.5, 7.2 Hz, 1H), 7.73 (d, J = 2.4 Hz, 1H), 7.61 (dd, J = 7.8, 1.7 Hz, 1H), 7.59 – 7.53 (m, 2H), 7.53 – 7.46 (m, 1H), 7.46 – 7.40 (m, 2H), 7.08 (dd, J = 8.8, 2.3 Hz, 1H), 6.96 (td, J = 7.4, 1.4 Hz, 1H), 6.90 (ddd, J = 8.7, 7.1, 1.6 Hz, 1H), 5.91 (dd, J = 8.1, 1.3 Hz, 1H), 5.86 (d, J = 8.8 Hz, 1H), 1.82 (s, 3H), 1.72 (s, 3H). ^{13}C NMR (176 MHz, Chloroform-d) δ 164.11, 163.77, 144.03, 139.84, 139.41, 135.34, 133.06, 132.60, 132.28, 130.82, 130.79, 130.73, 130.31, 129.68, 129.63, 129.59, 129.06, 129.03, 128.74, 128.41, 127.11, 126.11, 124.13, 123.55, 121.88, 115.79, 114.29, 113.89, 36.40, 32.85, 31.99. MALDI-MS: m/z [M$^-$] calcd for $C_{33}H_{23}N_2O_2Br$ 558.0948 found 558.0947.

Synthesis of t-ND-Br (Compound 4)

The synthesis of t-ND-Br (**4**) is similar to that of ND-Br, with the difference that the material is replaced

by t-ND, 70% yield. ^{1}H NMR (400 MHz, Chloroform-*d*) δ 8.86 (d, *J* = 7.7 Hz, 1H), 8.70 (dd, *J* = 7.2, 1.2 Hz, 1H), 8.03 (dd, *J* = 8.5, 1.2 Hz, 1H), 7.81 (d, *J* = 7.7 Hz, 1H), 7.69 (dd, *J* = 8.4, 7.2 Hz, 1H), 7.63 – 7.58 (m, 3H), 7.54 (dd, *J* = 7.8, 1.5 Hz, 1H), 7.28 (d, *J* = 8.5 Hz, 2H), 7.02 – 6.94 (m, 2H), 6.89 (ddd, *J* = 8.6, 7.2, 1.6 Hz, 1H), 5.99 (dd, *J* = 8.2, 1.2 Hz, 1H), 5.87 (d, *J* = 8.8 Hz, 1H), 1.80 (d, *J* = 35.6 Hz, 6H), 1.40 (s, 9H). ^{13}C NMR (176 MHz, Chloroform-*d*) δ 164.20, 163.86, 151.76, 143.93, 139.84, 139.41, 133.02, 132.57, 132.48, 132.26, 130.76, 130.67, 130.22, 129.91, 129.67, 129.57, 129.05, 128.39, 128.04, 127.10, 126.66, 126.10, 124.19, 123.62, 121.85, 115.78, 114.28, 113.86, 36.39, 34.94, 32.82, 32.02, 31.52. MALDI-MS: m/z [M^-] calcd for $C_{37}H_{31}N_2O_2Br$ 614.1574 found 614.1574.

Synthesis of ND-BO (Compound 5)

ND-Br (840 mg, 1.5 mmol), bis(pinacolato)diboron (600 mg, 2.36 mmol), potassium acetate (442 mg, 4.5 mmol) and [1,1'-bis(diphenylphosphino)ferrocene]dichloropalladium(II) (32.9 mg, 0.045 mmol) were added to a 100 mL two-necked vial under an argon atmosphere. Then 20 mL 1,4-dioxam was injected as solvent. After stirring at 105 ºC for 18 h, the reaction was cooled down to room temperature. Then the mixture was washed three times by saturated salt water and extracted by ethyl acetate. The obtained solution was dried with anhydrous magnesium sulfate, then filtered, and concentrated under reduced pressure. The crude product was further purified by column chromatography on silica gel using petroleum ether/ ethyl acetate (v/v = 9/1) as eluent to obtain ND-BO (**5**) as an orange solid (640 mg, 70% yield). ^{1}H NMR (400 MHz, Chloroform-*d*) δ 8.87 (d, *J* = 7.6 Hz, 1H), 8.69 (dd, *J* = 7.2, 1.2 Hz, 1H), 8.05 (dd, *J* = 8.5, 1.2 Hz, 1H), 7.99 (d, *J* = 1.4 Hz, 1H), 7.83 (d, *J* = 7.7 Hz, 1H), 7.66 (dd, *J* = 8.5, 7.2 Hz, 1H), 7.63 – 7.55 (m, 3H), 7.55 – 7.49 (m, 1H), 7.40 – 7.36 (m, 2H), 7.33 (dd, *J* = 8.2, 1.4 Hz, 1H), 6.98 (td, *J* = 7.5, 1.3 Hz, 1H), 6.88 (ddd, *J* = 8.6, 7.2, 1.5 Hz, 1H), 5.97 (dd, *J* = 7.6, 2.8 Hz, 2H), 1.90 (s, 3H), 1.80 (s, 3H), 1.33 (s, 12H).^{13}C NMR (101 MHz, Chloroform-*d*) δ 164.20, 163.87, 144.31, 142.51, 139.83, 135.40, 133.78, 133.08, 133.04, 132.53, 130.82, 130.79, 130.72, 130.52, 129.62, 129.23, 128.99, 128.76, 128.28, 126.85, 126.26, 124.04, 123.42, 121.82, 114.33, 113.56, 85.26, 36.17, 33.24, 32.38, 24.98. MALDI-MS: m/z [M^+] calcd for $C_{39}H_{36}BN_2O_4$ 607.2759 found 607.2758.

Synthesis of t-ND-BO (Compound 6)

The synthesis of t-ND-BO (**6**) is similar to that of ND-BO, with the difference that the material is replaced by t-ND-Br, 78% yield. ^{1}H NMR (400 MHz, Chloroform-*d*) δ 8.86 (d, *J* = 7.7 Hz, 1H), 8.68 (dd, *J* = 7.2, 1.2 Hz, 1H), 8.04 (dd, *J* = 8.5, 1.2 Hz, 1H), 7.99 (d, *J* = 1.4 Hz, 1H), 7.82 (d, *J* = 7.6 Hz, 1H), 7.65 (dd, *J* = 8.5, 7.2 Hz, 1H), 7.63 – 7.58 (m, 2H), 7.56 (dd, *J* = 7.8, 1.5 Hz, 1H), 7.33 (dd, *J* = 8.3, 1.4 Hz, 1H), 7.31 – 7.27 (m, 2H), 6.98 (td, *J* = 7.5, 1.2 Hz, 1H), 6.87 (ddd, *J* = 8.6, 7.2, 1.5 Hz, 1H), 6.04 – 5.91 (m, 2H), 1.85 (d, *J* = 38.7 Hz, 6H), 1.40 (s, 9H), 1.32 (s, 12H). ^{13}C NMR (101 MHz, Chloroform-*d*) δ 164.29, 163.97, 151.70, 144.23, 142.52, 139.84, 133.78, 133.04, 132.56, 132.50, 130.78, 130.71, 130.43, 129.22, 128.27, 128.07, 126.85, 126.65, 126.25, 124.12, 123.50, 121.81, 114.33, 113.56, 83.68, 36.17, 34.93, 33.22, 32.41, 31.53, 24.97. MALDI-MS: m/z [M^+] calcd for $C_{43}H_{44}N_2O_4$ 663.3396 found 663.3383.

Synthesis of T-TNP (Compound 7)

ND-BO (400 mg, 0.66 mmol), 3,9,17-tribromo-triperyleno[3,3,3]propellane (TNP-3Br) (mixture of isomers) (140.5 mg, 0.22 mmol), tetrakis(triphenylphosphine)palladium (38.1 mg, 0.03 mmol) and potassium carbonate (273.4 mg, 2 mmol) were added to a 100 mL two-necked vial under an argon atmosphere. 6 mL toluene, 1 mL water and 2 mL ethanol were injected sequentially as mixed solvent. After stirring at 90 ºC for 24 h, the reaction was cooled down to room temperature. Then the mixture was washed three times by saturated salt water and extracted by dichloromethane. The obtained solution was

dried with anhydrous magnesium sulfate, then filtered, and concentrated under reduced pressure. The crude product was further purified by column chromatography on silica gel using petroleum ether/ dichloromethane (v/v = 1/2) as eluent to obtain T-TNP(**7**) as a red solid (mixture of isomers) (230 mg, 57% yield, 99.91% purity). ^{1}H NMR (400 MHz, Chloroform-*d*) δ 8.88 (d, *J* = 7.7 Hz, 3H), 8.71 (dd, *J* = 7.2, 1.2 Hz, 3H), 8.18 (dd, *J* = 8.5, 1.2 Hz, 3H), 8.11 (tt, *J* = 7.1, 3.8 Hz, 6H), 7.88 (d, *J* = 7.7 Hz, 3H), 7.77 – 7.70 (m, 6H), 7.64 – 7.48 (m, 21H), 7.36 (dd, *J* = 8.3, 1.3 Hz, 6H), 7.02 – 6.94 (m, 6H), 6.90 (ddd, *J* = 8.7, 7.3, 1.6 Hz, 3H), 6.06 (d, *J* = 8.5 Hz, 3H), 6.02 (dd, *J* = 8.2, 1.3 Hz, 3H), 1.84 (s, 9H), 1.78 (s, 9H). ^{13}C NMR (101 MHz, $CDCl_3$) δ 164.19, 163.84, 147.07, 145.85, 144.58, 140.06, 139.45, 137.70, 137.60, 135.38, 133.12, 132.88, 132.58, 132.49, 131.03, 130.94, 130.81, 130.61, 130.03, 129.96, 129.62, 129.02, 128.83, 128.75, 128.37, 128.07, 127.79, 126.99, 126.35, 124.05, 123.35, 123.08, 121.59, 119.41, 119.27, 119.18, 114.26, 114.19, 79.41, 78.80, 36.28, 33.14, 32.68. MALDI-MS: m/z [M^+] calcd for $C_{131}H_{84}N_6O_6$ 1836.6446 found 1836.6423.

Synthesis of tBu-T-TNP (Compound 8)

The synthesis of tBu-T-TNP (**8**) is similar to that of T-TNP, with the difference that the material ND-BO is replaced by t-ND-BO. In addition, the eluent ratio of column chromatography was changed to petroleum ether/ dichloromethane (v/v = 1/1). tBu-T-TNP (**8**) was also a red solid (mixture of isomers), (280 mg, 63% yield, 99.99% purity). ^{1}H NMR (400 MHz, Chloroform-*d*) δ 8.87 (d, *J* = 7.7 Hz, 3H), 8.70 (dd, *J* = 7.3, 1.2 Hz, 3H), 8.17 (dd, *J* = 8.5, 1.2 Hz, 3H), 8.10 (tt, *J* = 7.1, 3.8 Hz, 6H), 7.87 (d, *J* = 7.7 Hz, 3H), 7.76 – 7.68 (m, 6H), 7.64 – 7.57 (m, 9H), 7.57 – 7.48 (m, 9H), 7.28 (d, *J* = 8.4 Hz, 6H), 7.01 – 6.93 (m, 6H), 6.89 (td, *J* = 7.7, 7.2, 1.6 Hz, 3H), 6.08 – 5.98 (m, 6H), 1.80 (d, *J* = 22.5 Hz, 18H), 1.40 (s, 27H). ^{13}C NMR (101 MHz, Chloroform-*d*) δ 164.28, 163.94, 151.73, 147.07, 145.85, 144.49, 140.06, 139.45, 137.70, 137.60, 133.09, 132.87, 132.53, 130.98, 130.94, 130.79, 130.52, 130.02, 129.95, 129.04, 128.82, 128.35, 128.06, 127.79, 126.98, 126.65, 126.34, 124.12, 123.42, 123.08, 121.57, 119.41, 119.18, 114.26, 79.41, 78.82, 36.27, 34.93, 33.10, 32.72, 31.52. MALDI-MS: m/z [M^-] calcd for $C_{143}H_{106}N_6O_6$ 2004.834 found 2004.832.

Synthesis of D-pBr (Compound 9) and S-pBr (Compound 10)

ND-BO (542 mg, 0.90 mmol), TNP-3Br (440 mg, 0.69 mmol), potassium carbonate (214.07 mg, 1.54 mmol) and tetrakis(triphenylphosphine)palladium (30 mg, 0.026 mmol) were added to a 50 mL two-necked vial under an argon atmosphere. 6 mL toluene, 1 mL water and 2 mL ethanol were injected sequentially as mixed solvent. After stirring at 90 ºC for 24 h, the reaction was cooled down to room temperature. Then the mixture was washed three times by saturated salt water and extracted by dichloromethane. The obtained solution was dried with anhydrous magnesium sulfate, then filtered, and concentrated under reduced pressure. The crude product was further purified by column chromatography on silica gel using petroleum ether/ dichloromethane (v/v = 1/2) as eluent. D-pBr (**9**, mixture of isomers) (171 mg, 26.4% yield) and S-pBr (**10**, mixture of isomers) (245 mg, 26.2% yield) were collected successively.

D-pBr (**9**), ^{1}H NMR (400 MHz, Chloroform-*d*) δ 8.89 (d, *J* = 7.7 Hz, 2H), 8.75 – 8.67 (m, 2H), 8.21 – 8.15 (m, 2H), 8.09 (dtt, *J* = 19.8, 7.5, 3.8 Hz, 5H), 7.97 – 7.91 (m, 1H), 7.88 (d, *J* = 7.7 Hz, 2H), 7.83 (d, *J* = 8.4 Hz, 1H), 7.79 (d, *J* = 7.4 Hz, 1H), 7.78 – 7.70 (m, 4H), 7.70 – 7.65 (m, 1H), 7.64 – 7.48 (m, 14H), 7.37 (dd, *J* = 7.2, 1.8 Hz, 4H), 7.03 – 6.94 (m, 4H), 6.90 (td, *J* = 7.8, 7.3, 1.6 Hz, 2H), 6.12 – 5.97 (m, 4H), 1.81 (d, *J* = 23.8 Hz, 12H). ^{13}C NMR (176 MHz, Chloroform-*d*) δ 164.18, 163.83, 147.07, 146.65, 146.53, 145.78, 145.40, 145.29, 144.57, 140.06, 139.51, 138.28, 137.79, 137.59, 136.44, 135.39, 133.11, 132.77, 132.57, 131.90, 131.81, 131.03, 130.94, 130.82, 130.59, 130.07, 129.97, 129.61,

129.05, 129.00, 128.85, 128.76, 128.36, 128.07, 127.80, 126.99, 126.34, 124.07, 123.94, 123.38, 123.25, 121.61, 120.30, 120.01, 119.44, 119.40, 119.17, 114.27, 79.52, 78.72, 36.28, 33.12, 32.67. MALDI-MS: m/z [M^-] calcd for $C_{98}H_{61}N_4O_4Br$ 1436.3870 found 1436.3827.

S-pBr (**10**), ^{1}H NMR (400 MHz, Chloroform-*d*) δ 8.89 (d, *J* = 7.7 Hz, 1H), 8.71 (d, *J* = 7.2 Hz, 1H), 8.18 (d, *J* = 8.4 Hz, 1H), 8.14 – 7.98 (m, 4H), 7.95 – 7.86 (m, 3H), 7.83 (d, *J* = 8.4 Hz, 2H), 7.79 (d, *J* = 7.5 Hz, 2H), 7.77 – 7.70 (m, 2H), 7.66 (t, *J* = 7.7 Hz, 2H), 7.62 – 7.50 (m, 7H), 7.37 (d, *J* = 7.6 Hz, 2H), 6.97 (dd, *J* = 8.4, 2.1 Hz, 2H), 6.94 – 6.86 (m, 1H), 6.04 (dd, *J* = 16.0, 8.3 Hz, 2H), 1.81 (d, *J* = 23.8 Hz, 6H). ^{13}C NMR (101 MHz, Chloroform-*d*) δ 164.36, 164.01, 147.25, 146.83, 146.71, 145.58, 145.47, 144.75, 140.24, 139.69, 138.46, 137.98, 137.77, 135.57, 133.29, 132.95, 132.75, 132.08, 131.99, 131.21, 131.12, 131.00, 130.77, 130.25, 130.15, 129.79, 129.24, 129.17, 129.03, 128.94, 128.54, 128.25, 127.98, 127.17, 126.52, 124.56, 124.25, 124.12, 123.56, 123.43, 121.79, 120.48, 120.19, 119.62, 119.58, 119.40, 119.35, 114.45, 79.70, 78.43, 36.47, 33.30, 32.85. HRMS (ESI, positive): m/z $(M)^+$ calcd for $C_{65}H_{38}N_2O_2Br_2$ 1036.1295 found 1036.1313.

Synthesis of tBu-S-pBr (Compound 11)

The synthesis of tBu-S-pBr (**11**) is similar to that of tBu-T-TNP (**8**), with the difference that the material t-ND-BO feed 0.9 eq. tBu-S-pBr (**11**) was a pink solid (110 mg, 43% yield. ^{1}H NMR (400 MHz, Chloroform-*d*) δ 8.87 (d, *J* = 7.7 Hz, 1H), 8.70 (dd, *J* = 7.3, 1.1 Hz, 1H), 8.16 (dd, *J* = 8.4, 1.2 Hz, 1H), 8.13 – 7.98 (m, 4H), 7.93 – 7.62 (m, 10H), 7.62 – 7.56 (m, 3H), 7.56 – 7.52 (m, 2H), 7.50 (d, *J* = 7.1 Hz, 1H), 7.28 (s, 2H), 7.01 – 6.93 (m, 2H), 6.89 (td, *J* = 7.7, 7.3, 1.5 Hz, 1H), 6.03 (ddd, *J* = 14.3, 8.4, 1.2 Hz, 2H), 1.80 (d, *J* = 22.4 Hz, 6H), 1.39 (s, 9H). ^{13}C NMR (176 MHz, Chloroform-*d*) δ 164.27, 163.93, 151.74, 146.64, 146.52, 146.20, 146.08, 144.80, 144.45, 140.05, 139.55, 138.13, 137.96, 137.47, 133.07, 132.63, 132.54, 131.89, 131.85, 130.98, 130.92, 130.80, 130.50, 130.08, 129.98, 129.95, 129.07, 128.87, 128.35, 128.06, 127.80, 127.00, 126.65, 126.33, 124.14, 124.11, 123.46, 123.41, 121.61, 120.33, 120.27, 120.05, 119.99, 119.42, 119.36, 119.22, 114.28, 114.22, 79.43, 78.78, 36.29, 34.93, 33.09, 32.71, 31.53. MALDI-MS: m/z [M^-] calcd for $C_{69}H_{46}N_2O_2Br_2$ 1092.1931 found 1092.1946.

Synthesis of D-DMAc (Compound 12)

D-pBr (171 mg, 0.12 mmol), 9,9-dimethyl-10H-acridine (50 mg, 0.24 mmol), palladium(II)acetate (2.7 mg, 0.012 mmol), tri-tert-butylphosphine tetrafluoroborate (6.9 mg, 0.024 mmol) and sodium tert-butoxide (45.7 mg, 0.48 mmol) were added to a 50 mL two-necked vial under an argon atmosphere. 5 mL toluene was injected sequentially as solvent. After stirring at 110 ºC for 24 h, the reaction was cooled down to room temperature. Then the mixture was washed three times by saturated salt water and extracted by dichloromethane. The obtained solution was dried with anhydrous magnesium sulfate, then filtered, and concentrated under reduced pressure. The crude product was further purified by column chromatography on silica gel using petroleum ether/ dichloromethane (v/v = 1/1) as eluent. The product obtained was recrystallized with ethanol and acetone, then dried under reduced pressure to obtain D-DMAc (**12**) (mixture of isomers) as a red solid (63 mg, 34% yield, 99.98% purity). ^{1}H NMR (400 MHz, Chloroform-*d*) δ 8.89 (d, *J* = 7.7 Hz, 2H), 8.71 (d, *J* = 7.3 Hz, 2H), 8.25 – 8.06 (m, 8H), 7.89 (d, *J* = 7.7 Hz, 2H), 7.80 (dd, *J* = 8.5, 4.2 Hz, 2H), 7.73 (t, *J* = 7.8 Hz, 2H), 7.69 – 7.48 (m, 17H), 7.45 (d, *J* = 7.5 Hz, 2H), 7.36 (d, *J* = 7.3 Hz, 4H), 6.99 (q, *J* = 8.0, 6.9 Hz, 4H), 6.90 (t, *J* = 7.7 Hz, 2H), 6.84 (t, *J* = 7.5 Hz, 2H), 6.74 (t, *J* = 7.9 Hz, 2H), 6.07 (d, *J* = 8.5 Hz, 2H), 6.05 – 5.94 (m, 4H), 1.92 – 1.74 (m, 18H). ^{13}C NMR (101 MHz, Chloroform-*d*) δ 164.29, 163.85, 147.35, 147.28, 146.90, 145.65, 144.57, 140.65, 140.06, 139.50, 137.92, 137.84, 137.70, 136.01, 135.84, 135.37, 133.14, 132.75, 132.60, 131.70, 131.04,

130.95, 130.83, 130.61, 130.11, 130.03, 129.80, 129.63, 129.15, 129.01, 128.76, 128.38, 128.10, 127.87, 126.96, 126.58, 126.35, 125.59, 125.29, 124.07, 123.32, 121.97, 121.62, 121.28, 120.56, 120.34, 119.64, 119.40, 114.50, 114.27, 80.88, 79.21, 36.31, 36.10, 33.14, 32.99, 32.72, 31.86, 31.32. MALDI-MS: m/z [M^+] calcd for $C_{113}H_{76}N_5O_4$ 1566.5892 found 1566.5883.

Synthesis of S-DMAc (Compound 13) and tBu-S-DMAc (Compound 14)

The synthesis of S-DMAc (**13**) and tBu-S-DMAc (**14**) is similar to that of D-DMAc (**12**), with the difference that the material 9,9-dimethyl-10H-acridine feed 3 eq and S-pBr and tBu-S-pBr were used instead of D-pBr.

S-DMAc (**13**) was obtained in pink soild (mixture of isomers) with 54% yield, 99.98% purity. ^{1}H NMR (400 MHz, Chloroform-*d*) δ 8.90 (d, *J* = 7.7 Hz, 1H), 8.72 (dd, *J* = 7.2, 1.2 Hz, 1H), 8.35 – 8.06 (m, 7H), 7.90 (d, *J* = 7.8 Hz, 1H), 7.89 – 7.84 (m, 1H), 7.75 (dd, *J* = 8.5, 7.2 Hz, 1H), 7.70 (d, *J* = 2.6 Hz, 1H), 7.68 – 7.63 (m, 1H), 7.62 – 7.57 (m, 5H), 7.56 – 7.53 (m, 1H), 7.51 (d, *J* = 7.5 Hz, 1H), 7.47 (td, *J* = 6.2, 5.1, 2.9 Hz, 5H), 7.42 (dd, *J* = 8.3, 2.6 Hz, 2H), 7.37 (dd, *J* = 7.2, 1.7 Hz, 2H), 7.06 (dq, *J* = 8.4, 2.8 Hz, 1H), 6.99 (t, *J* = 7.5 Hz, 1H), 6.95 – 6.79 (m, 7H), 6.79 – 6.71 (m, 2H), 6.15 (dt, *J* = 7.7, 2.1 Hz, 2H), 6.10 (dt, *J* = 8.5, 1.7 Hz, 1H), 6.06 – 5.95 (m, 3H), 1.91 – 1.84 (m, 3H), 1.81 (d, *J* = 6.1 Hz, 9H), 1.73 (s, 6H). ^{13}C NMR (101 MHz, Chloroform-*d*) δ 164.19, 163.85, 147.20, 147.14, 147.08, 145.47, 144.55, 140.64, 140.06, 139.67, 137.99, 137.70, 135.74, 135.65, 135.38, 133.14, 132.60, 131.88, 131.38, 131.31, 131.04, 130.96, 130.92, 130.84, 130.60, 130.17, 129.97, 129.85, 129.63, 129.01, 128.76, 128.40, 128.15, 127.93, 127.02, 126.70, 126.59, 126.35, 125.67, 125.60, 124.08, 123.40, 121.66, 121.54, 121.45, 120.65, 120.29, 119.71, 114.51, 114.30, 80.33, 79.52, 48.20, 36.34, 36.12, 33.12, 32.72, 32.66, 31.98. HRMS (ESI, positive): m/z $(M+H)^+$ calcd for $C_{95}H_{66}N_4O_2$ 1295.5259 found 1295.5198

tBu-S-DMAc (**14**) was also obtained in pink soild (mixture of isomers) with 63% yield, 99.99% purity. ^{1}H NMR (400 MHz, DMSO-d_6) δ 8.88 – 8.68 (m, 3H), 8.66 – 8.45 (m, 5H), 8.07 (dd, *J* = 13.5, 8.0 Hz, 2H), 7.89 (t, *J* = 7.9 Hz, 1H), 7.74 (t, *J* = 6.5 Hz, 15H), 7.34 (d, *J* = 8.2 Hz, 2H), 7.26 (dd, *J* = 8.4, 4.8 Hz, 2H), 7.07 (q, *J* = 7.3 Hz, 1H), 6.94 (dt, *J* = 19.1, 7.3 Hz, 2H), 6.88 – 6.66 (m, 8H), 6.05 (dd, *J* = 8.4, 2.9 Hz, 1H), 5.93 (dd, *J* = 15.6, 7.9 Hz, 3H), 5.80 – 5.69 (m, 2H), 1.90 – 1.63 (m, 19H), 1.38 (s, 9H). ^{13}C NMR (101 MHz, Chloroform-*d*) δ 164.37, 163.88, 152.09, 147.15, 145.36, 144.51, 140.65, 140.07, 139.66, 138.06, 137.69, 135.74, 135.61, 133.20, 132.57, 131.88, 131.40, 131.32, 131.01, 130.94, 130.83, 130.56, 130.17, 129.97, 129.86, 129.59, 129.13, 128.49, 128.15, 128.07, 127.93, 127.01, 126.69, 126.59, 126.34, 126.31, 125.68, 125.61, 124.16, 123.52, 123.40, 121.65, 121.55, 121.45, 120.64, 120.53, 120.21, 114.50, 114.35, 79.63, 78.35, 36.35, 36.13, 34.95, 33.09, 32.73, 32.00, 31.54, 29.86. MALDI-MS: m/z [M^+] calcd for $C_{99}H_{74}N_4O_2$ 1350.5806 found 1350.5783.

Synthesis of S-mCP (Compound 15)

S-pBr (52 mg, 0.05 mmol), 9,9'-(5-(4,4,5,5-tetramethyl-1,3,2-dioxaborolan-2-yl)-1,3-phenylene)bis(9H-carbazole) (mCP-BO) (53 mg, 0.1 mmol), potassium carbonate (62 mg, 0.45 mmol) and tetrakis(triphenylphosphine)palladium (7 mg, 0.08 mmol) were added to a 50 mL two-necked vial under an argon atmosphere. 6 mL toluene, 1 mL water and 2 mL ethanol were injected sequentially as mixed solvent. After stirring at 90 ºC for 24 h, the reaction was cooled down to room temperature. Then the mixture was washed three times by saturated salt water and extracted by dichloromethane. The obtained solution was dried with anhydrous magnesium sulfate, then filtered, and concentrated under reduced pressure. The crude product was further purified by column chromatography on silica gel using petroleum ether/ dichloromethane (v/v = 1/2) as eluent. The product obtained was recrystallized with ethanol and acetone, then dried under reduced pressure to obtain S-mCP (**15**) as a red solid (mixture of

isomers), (43 mg, 51% yield, 99.98% purity). ^{1}H NMR (400 MHz, Chloroform-*d*) δ 8.88 (d, *J* = 7.7 Hz, 1H), 8.71 (dd, *J* = 7.3, 1.2 Hz, 1H), 8.25 – 8.07 (m, 16H), 7.93 (dd, *J* = 8.4, 2.1 Hz, 2H), 7.90 – 7.85 (m, 3H), 7.80 (t, *J* = 1.8 Hz, 4H), 7.78 – 7.70 (m, 4H), 7.56 (dd, *J* = 27.0, 7.8 Hz, 18H), 7.41 (ddd, *J* = 8.3, 7.1, 1.2 Hz, 8H), 7.34 – 7.27 (m, 8H), 7.02 – 6.94 (m, 2H), 6.90 (td, *J* = 7.7, 7.2, 1.6 Hz, 1H), 6.08 – 5.99 (m, 2H), 1.80 (d, *J* = 23.7 Hz, 6H). ^{13}C NMR (101 MHz, Chloroform-*d*) δ 164.17, 163.84, 147.12, 145.86, 145.30, 144.56, 143.45, 142.54, 140.54, 140.05, 139.56, 137.72, 137.65, 135.85, 135.37, 133.18, 132.77, 132.41, 131.02, 130.94, 130.81, 130.59, 130.48, 130.05, 129.95, 129.84, 129.64, 129.01, 128.76, 128.37, 128.04, 127.71, 127.00, 126.57, 126.34, 124.28, 123.81, 123.57, 123.35, 122.62, 121.73, 120.59, 119.92, 119.32, 114.22, 109.90, 79.45, 78.92, 36.00, 33.14, 31.73. MALDI-MS: m/z [M^-] calcd for $C_{125}H_{76}N_6O_2$ 1692.602 found 1692.602.

Synthesis of tBu-S-mCP (Compound 16)

The synthesis of tBu-S-mCP (**16**) is similar to that of S-mCP. tBu-S-mCP (**16**) is also obtained in pink solid (mixture of isomers) with 53% yield, 99.98% purity. ^{1}H NMR (400 MHz, Chloroform-*d*) δ 8.87 (d, *J* = 7.7 Hz, 1H), 8.70 (dd, *J* = 7.3, 1.2 Hz, 1H), 8.26 – 8.06 (m, 14H), 7.92 (dd, *J* = 8.4, 2.1 Hz, 2H), 7.89 – 7.83 (m, 3H), 7.79 (t, *J* = 1.8 Hz, 4H), 7.78 – 7.68 (m, 4H), 7.67 – 7.49 (m, 16H), 7.40 (ddd, *J* = 8.3, 7.2, 1.2 Hz, 8H), 7.31 – 7.27 (m, 8H), 7.01 – 6.94 (m, 2H), 6.89 (ddd, *J* = 8.6, 7.2, 1.6 Hz, 1H), 6.07 – 6.00 (m, 2H), 1.80 (d, *J* = 22.4 Hz, 7H), 1.40 (s, 9H). ^{13}C NMR (101 MHz, Chloroform-*d*) δ 164.28, 163.94, 151.74, 147.19, 146.87, 145.54, 145.40, 144.45, 143.46, 140.55, 140.05, 139.57, 139.51, 137.72, 135.90, 133.07, 132.75, 132.54, 130.98, 130.91, 130.80, 130.48, 130.05, 129.95, 129.83, 129.66, 129.13, 128.91, 128.34, 128.06, 127.80, 126.66, 126.58, 126.34, 124.14, 123.81, 123.59, 123.42, 122.76, 121.59, 120.59, 119.32, 114.27, 109.90, 79.52, 78.54, 36.28, 34.94, 33.11, 32.72, 31.53. MALDI-MS: m/z [M^-] calcd for $C_{129}H_{84}N_6O_2$ 1748.665 found 1748.665.

Synthesis of S-pmCP (Compound 17)

The synthesis of S-pmCP (**17**) is similar to that of tBu-S-pBr, with the difference that the material t-ND-BO is replaced by mCP-BO. S-pmCP was obtained in grey solid (mixture of isomers) with 43% yield. ^{1}H NMR (400 MHz, Chloroform-*d*) δ 8.19 – 8.00 (m, 9H), 7.91 (dt, *J* = 7.8, 3.3 Hz, 1H), 7.87 (q, *J* = 2.2 Hz, 1H), 7.80 (t, *J* = 1.7 Hz, 2H), 7.73 (dd, *J* = 5.6, 3.3 Hz, 2H), 7.62 – 7.52 (m, 10H), 7.43 – 7.37 (m, 4H), 7.29 (t, *J* = 7.5 Hz, 4H). ^{13}C NMR (176 MHz, Chloroform-*d*) δ 146.96, 146.87, 146.39, 146.22, 145.97, 145.86, 145.78, 143.41, 143.29, 140.55, 139.60, 138.09, 137.50, 136.04, 132.64, 131.91, 131.88, 130.43, 130.02, 129.83, 129.65, 128.72, 126.59, 126.34, 124.22, 123.82, 122.80, 120.59, 119.29, 109.89, 79.40, 78.35. MALDI-MS: m/z [M^+] calcd for $C_{62}H_{34}O_2Br_2$ 964.1083 found 964.1074.

Synthesis of D-mCP (Compound 18) and tBu-D-mCP (Compound 19)

The synthesis of D-mCP (**18**) and tBu-D-mCP (**19**) is similar to that of T-TNP, with the difference that the material TNP-3Br is replaced by S-pmCP.

D-mCP (**18**) was obtained in pink solid (mixture of isomers) with 47% yield, 99.99% purity. ^{1}H NMR (400 MHz, Chloroform-*d*) δ 8.88 (d, *J* = 7.7 Hz, 2H), 8.70 (dd, *J* = 7.2, 1.2 Hz, 2H), 8.24 – 8.03 (m, 12H), 7.93 – 7.85 (m, 4H), 7.79 (d, *J* = 1.9 Hz, 2H), 7.77 – 7.69 (m, 5H), 7.65 – 7.49 (m, 19H), 7.44 – 7.33 (m, 8H), 7.32 – 7.27 (m, 4H), 7.02 – 6.93 (m, 4H), 6.89 (ddd, *J* = 8.7, 7.2, 1.6 Hz, 2H), 6.08 – 5.98 (m, 4H), 1.80 (d, *J* = 23.7 Hz, 12H). ^{13}C NMR (101 MHz, Chloroform-*d*) δ 164.19, 163.84, 147.38, 146.78, 145.63, 144.58, 143.37, 140.54, 140.05, 139.55, 139.48, 137.75, 137.68, 135.71, 135.38, 133.13, 132.83, 132.47, 131.03, 130.97, 130.94, 130.82, 130.61, 130.45, 130.05, 129.96, 129.62, 129.06, 129.01, 128.87, 128.76, 128.37, 128.06, 127.80, 126.96, 126.58, 126.34, 124.05, 123.81, 123.36, 121.60, 120.58, 119.35, 114.26, 109.91, 78.87, 78.32, 36.28, 33.14, 32.68, 1.17.MALDI-MS: m/z [M^-] calcd for

$C_{128}H_{80}N_6O_4$ 1764.624 found 1764.626.

tBu-D-mCP (**19**) was obtained in pink solid (mixture of isomers) with 54% yield, 99.98% purity. ^{1}H NMR (400 MHz, Chloroform-*d*) δ 8.87 (d, *J* = 7.7 Hz, 2H), 8.70 (dd, *J* = 7.2, 1.2 Hz, 2H), 8.23 – 8.04 (m, 11H), 7.95 – 7.84 (m, 4H), 7.79 (d, *J* = 1.9 Hz, 2H), 7.77 – 7.68 (m, 5H), 7.66 – 7.48 (m, 17H), 7.40 (ddd, *J* = 8.3, 7.1, 1.2 Hz, 4H), 7.29 (td, *J* = 7.2, 6.8, 1.1 Hz, 6H), 7.02 – 6.93 (m, 4H), 6.89 (ddd, *J* = 8.7, 7.1, 1.6 Hz, 2H), 6.10 – 5.97 (m, 4H), 1.80 (d, *J* = 22.4 Hz, 12H), 1.40 (s, 18H). ^{13}C NMR (101 MHz, Chloroform-*d*) δ 164.28, 163.94, 151.74, 147.43, 146.79, 145.62, 145.57, 144.48, 143.51, 140.54, 140.06, 139.54, 139.48, 137.71, 135.71, 133.08, 132.81, 132.54, 130.98, 130.92, 130.79, 130.52, 130.45, 130.03, 129.95, 129.62, 129.07, 128.87, 128.34, 128.06, 127.79, 126.98, 126.66, 126.58, 126.34, 124.12, 123.81, 123.54, 123.43, 121.58, 120.59, 119.34, 114.23, 109.90, 80.63, 78.94, 36.28, 34.94, 33.11, 32.72, 31.53. MALDI-MS: m/z [M^-] calcd for $C_{136}H_{96}N_6O_4$ 1876.749 found 1876.749.

Supplementary Note 3: NMR, HPLC and HRMS spectra

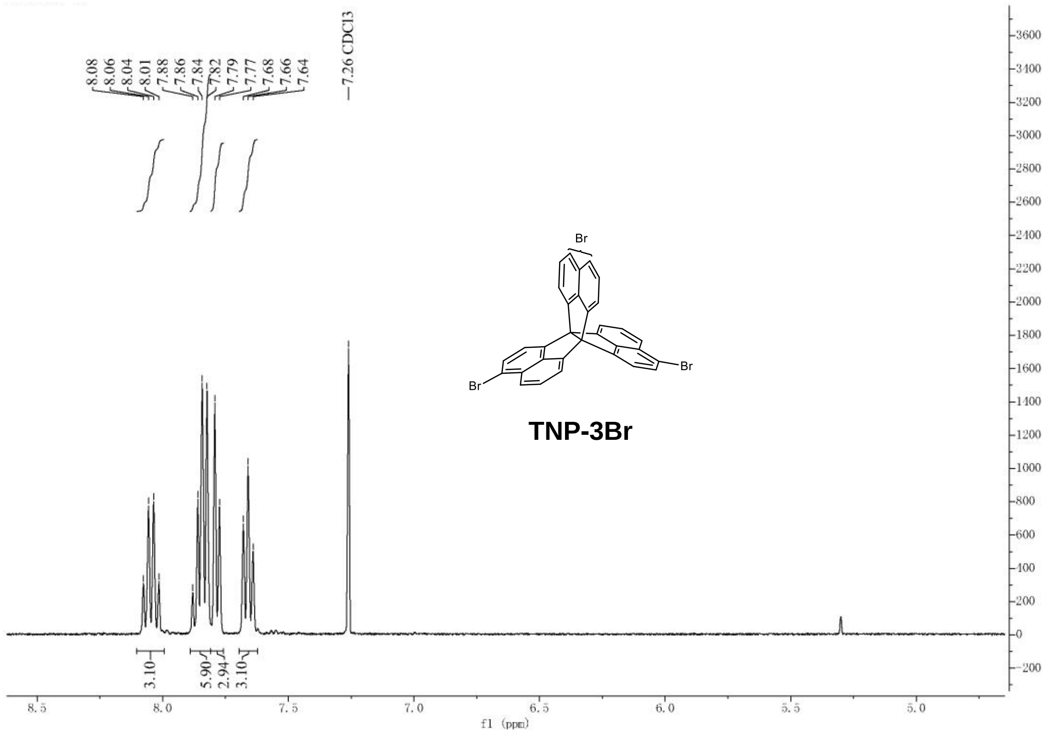

Supplementary Figure 2. [1]**H NMR spectrum of TNP-3Br in chloroform-*d*.**

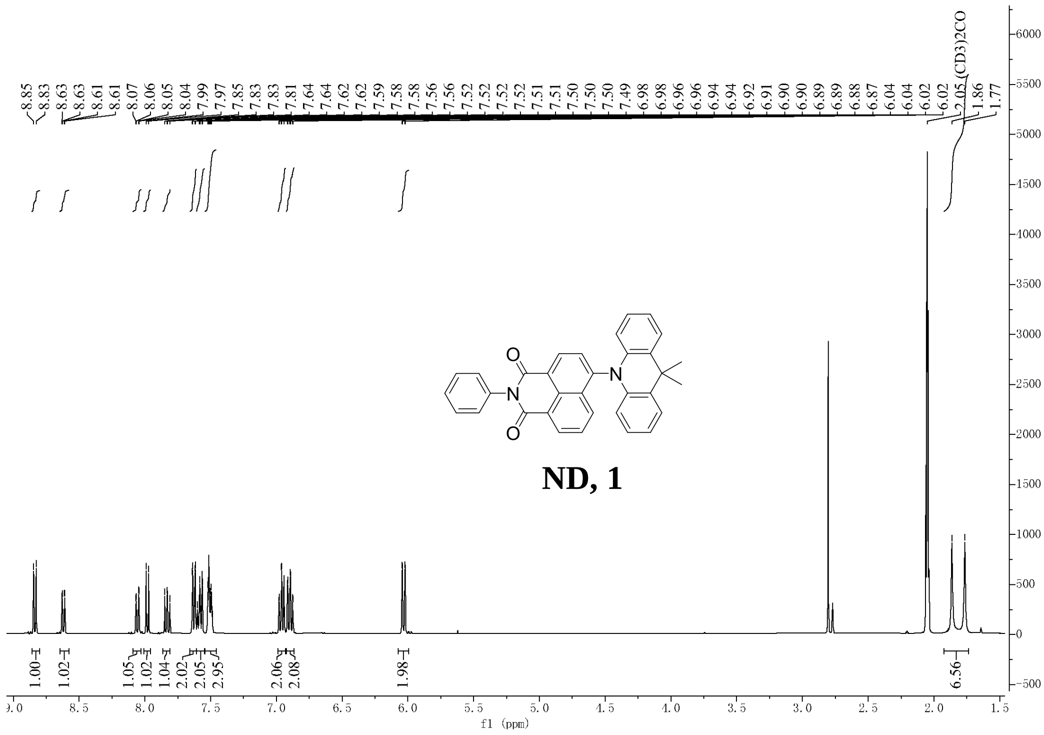

Supplementary Figure 3. [1]**H NMR spectrum of 1 in Acetone-*d*$_6$.**

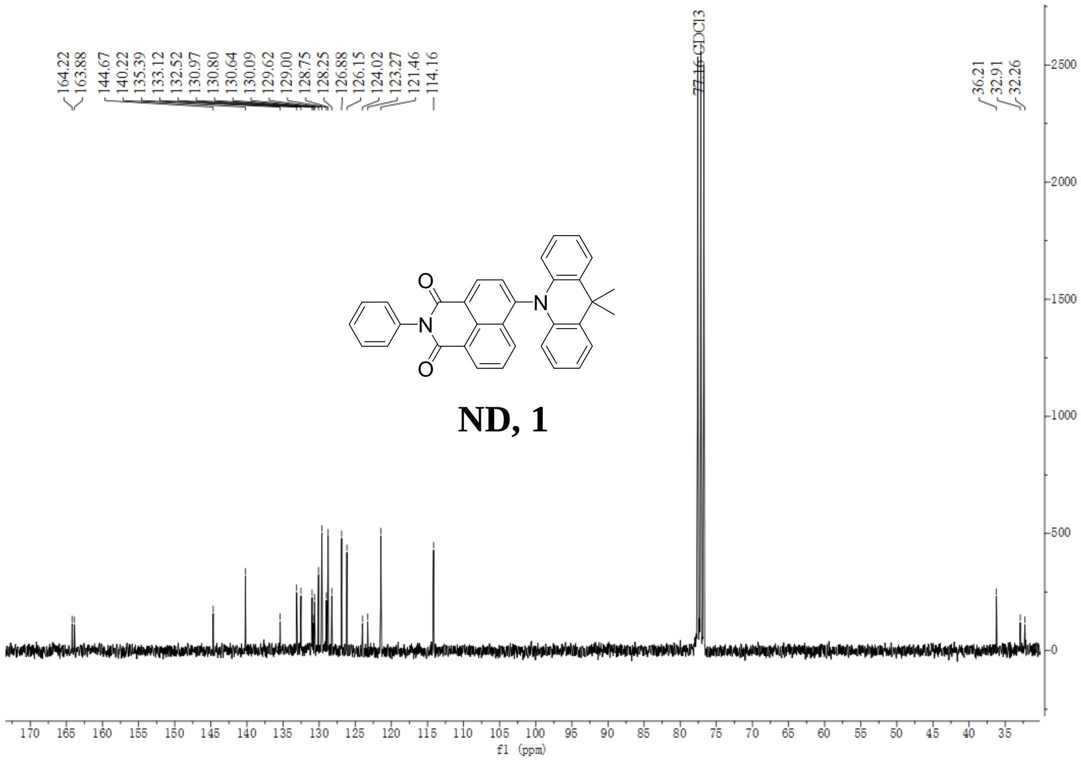

Supplementary Figure 4. ^{13}**C NMR spectrum of 1 in chloroform-*d*.**

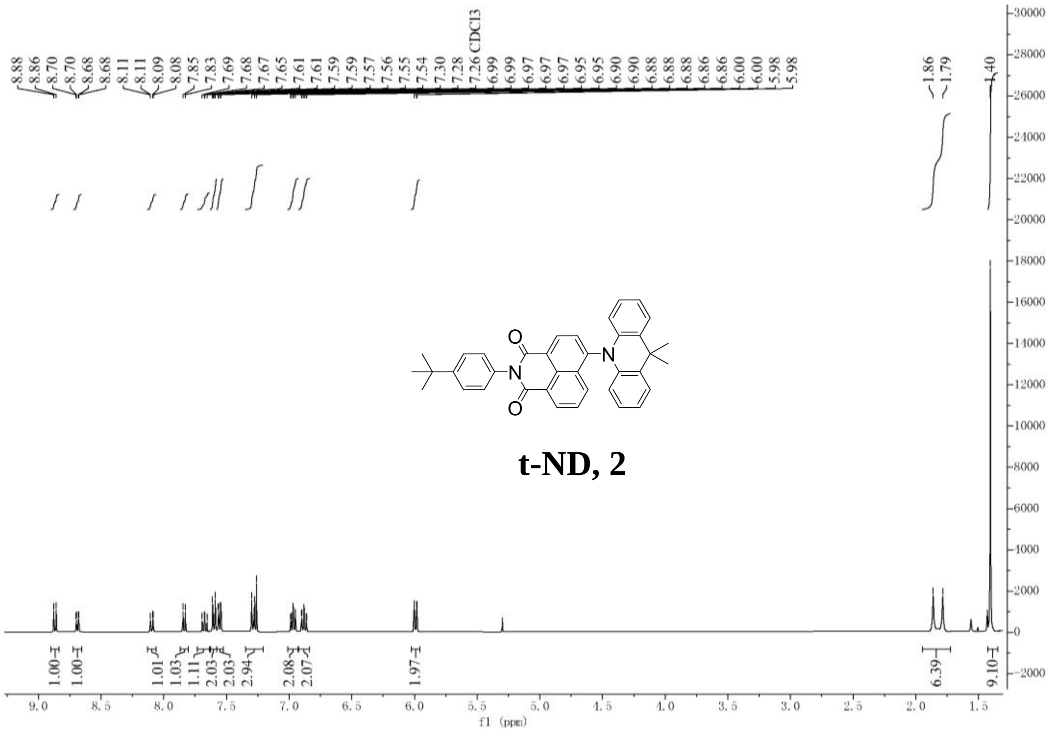

Supplementary Figure 5. ^{1}**H NMR spectrum of 2 in chloroform-*d*.**

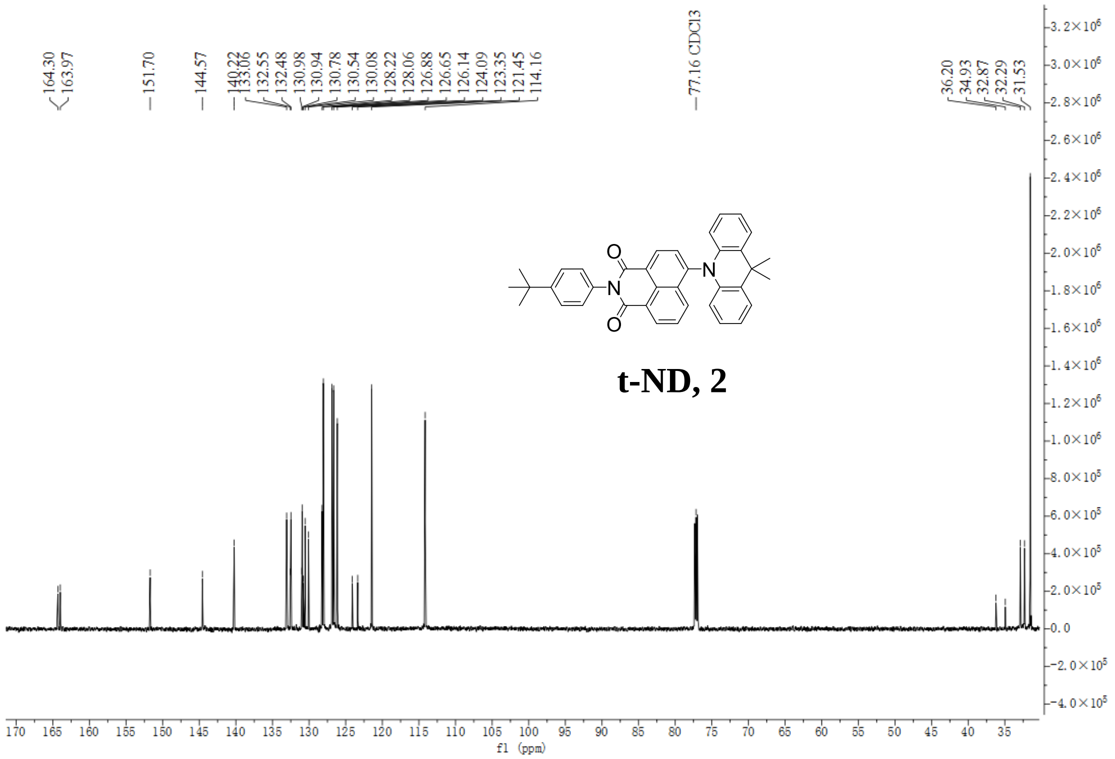

Supplementary Figure 6. ^{13}C NMR spectrum of 2 in chloroform-*d*.

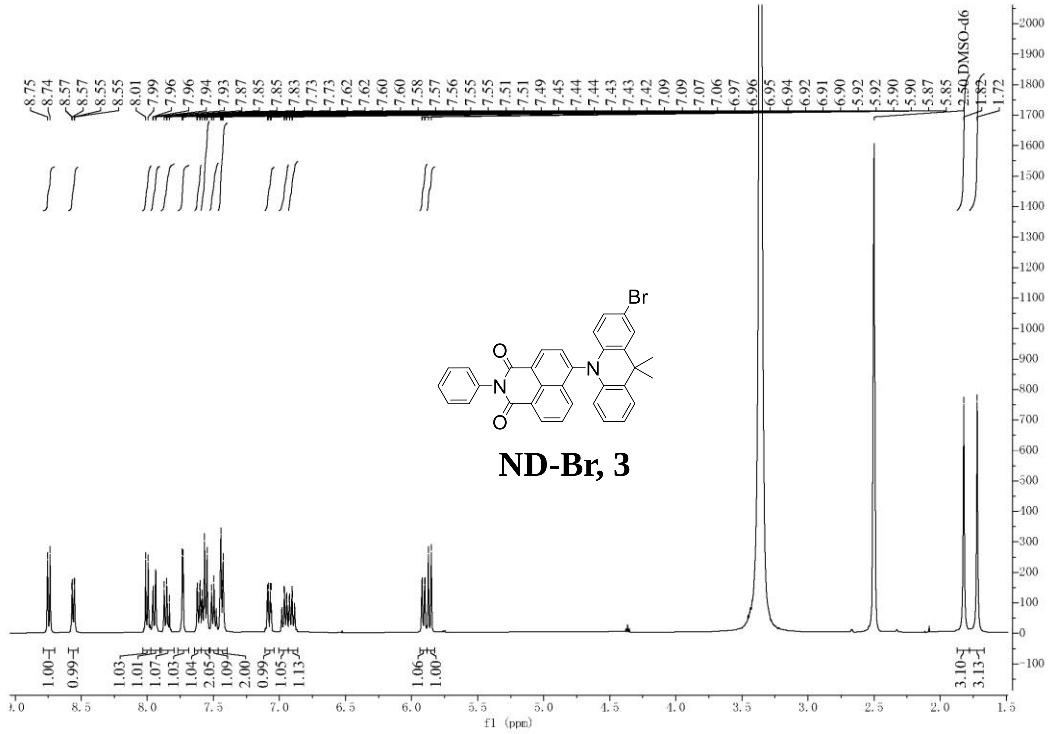

Supplementary Figure 7. ^{1}H NMR spectrum of 3 in DMSO-d_6.

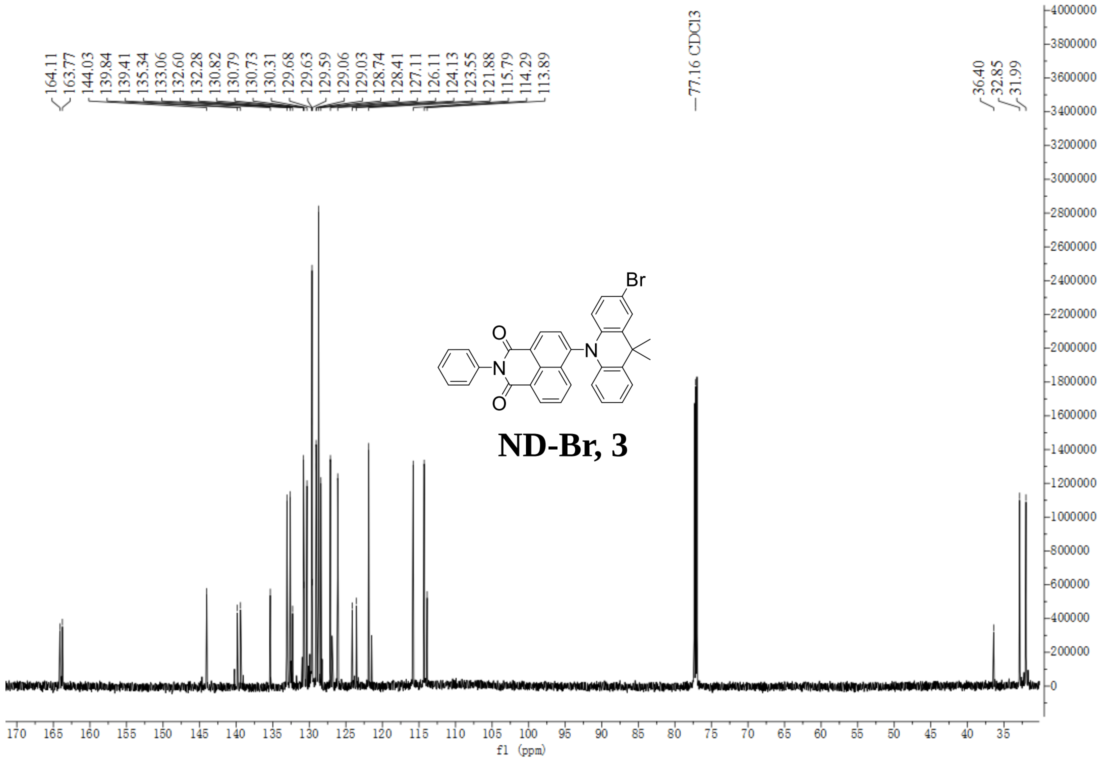

Supplementary Figure 8. ^{13}C NMR spectrum of 3 in chloroform-*d*.

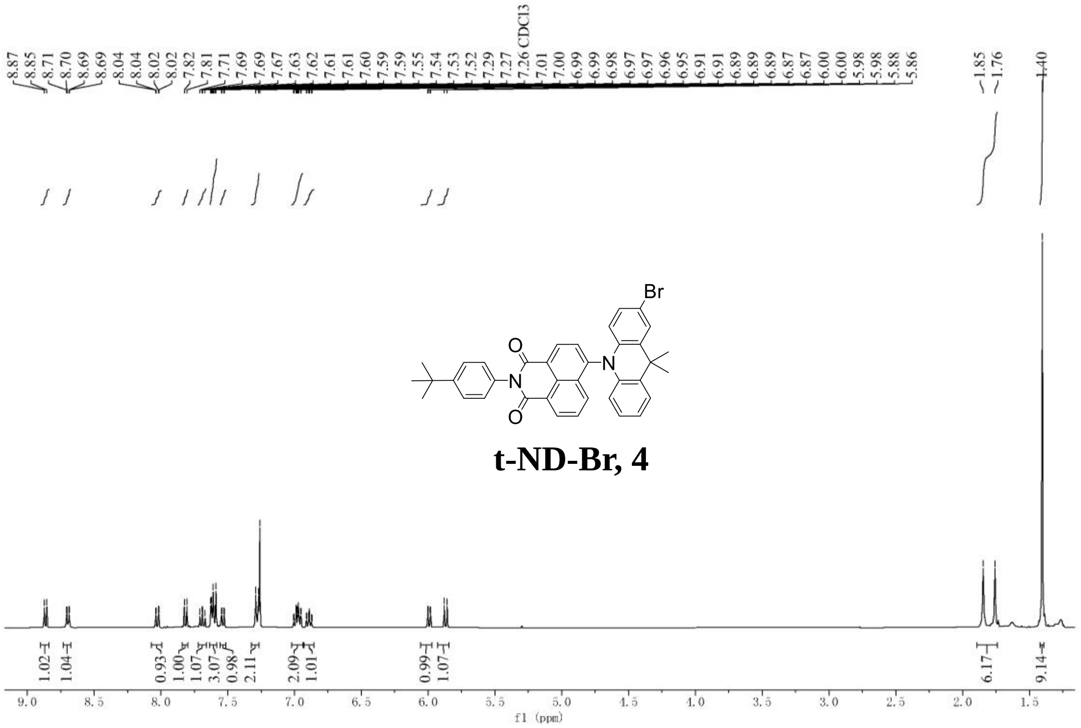

Supplementary Figure 9. ^{1}H NMR spectrum of 4 in chloroform-*d*.

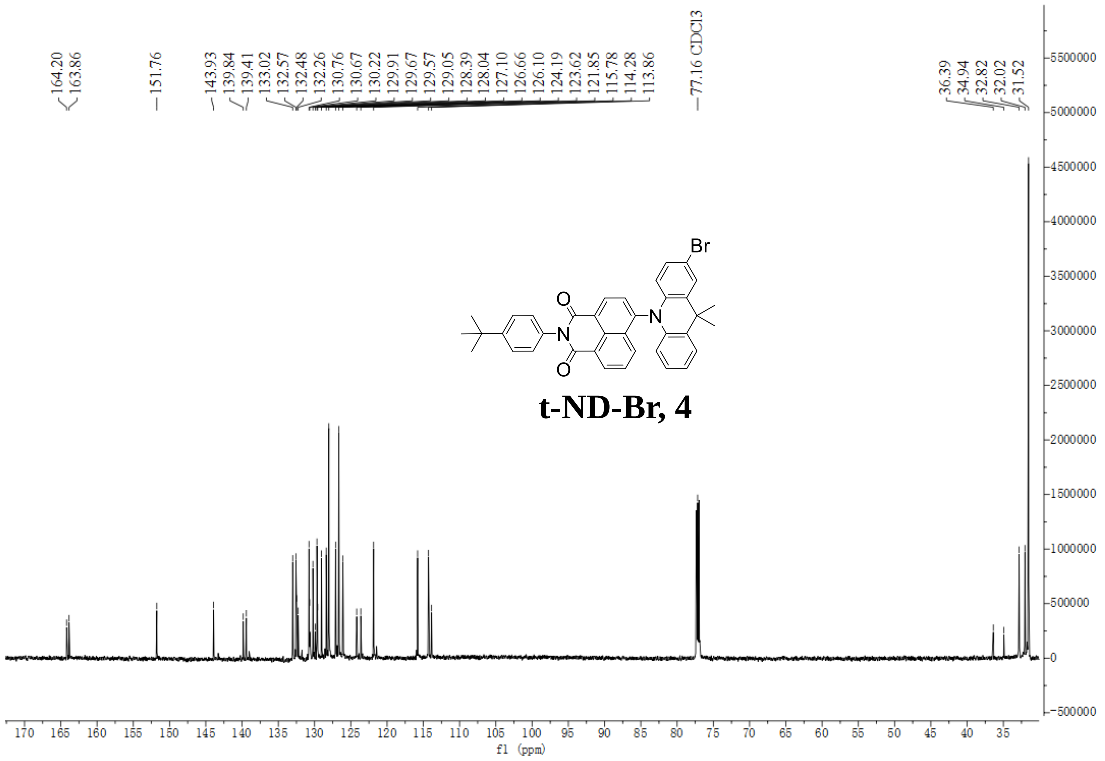

Supplementary Figure 10. ^{13}C **NMR spectrum of 4 in chloroform-*d*.**

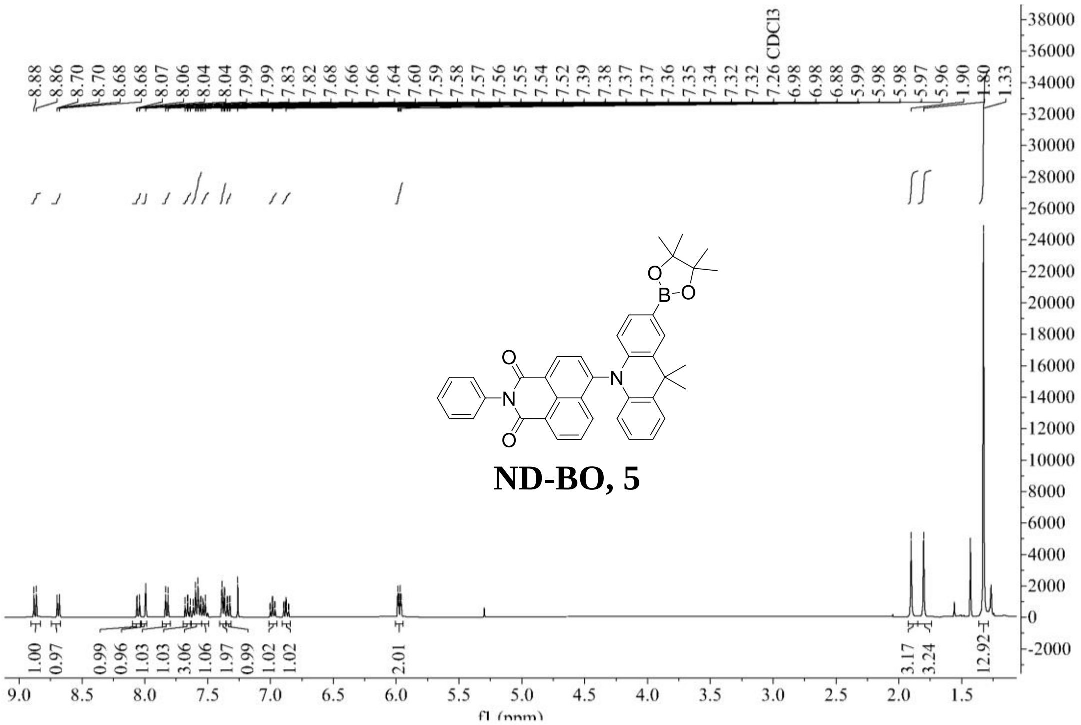

Supplementary Figure 11. ^{1}H **NMR spectrum of 5 in chloroform-*d*.**

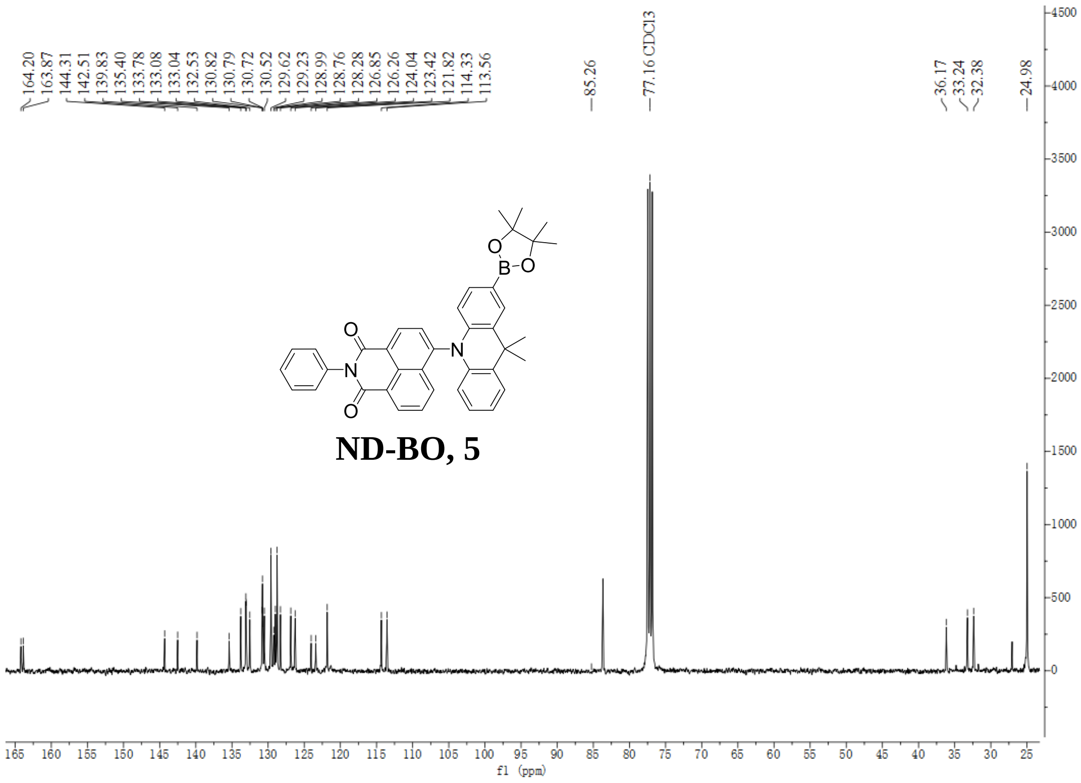

Supplementary Figure 12. ^{13}C NMR spectrum of 5 in chloroform-*d*.

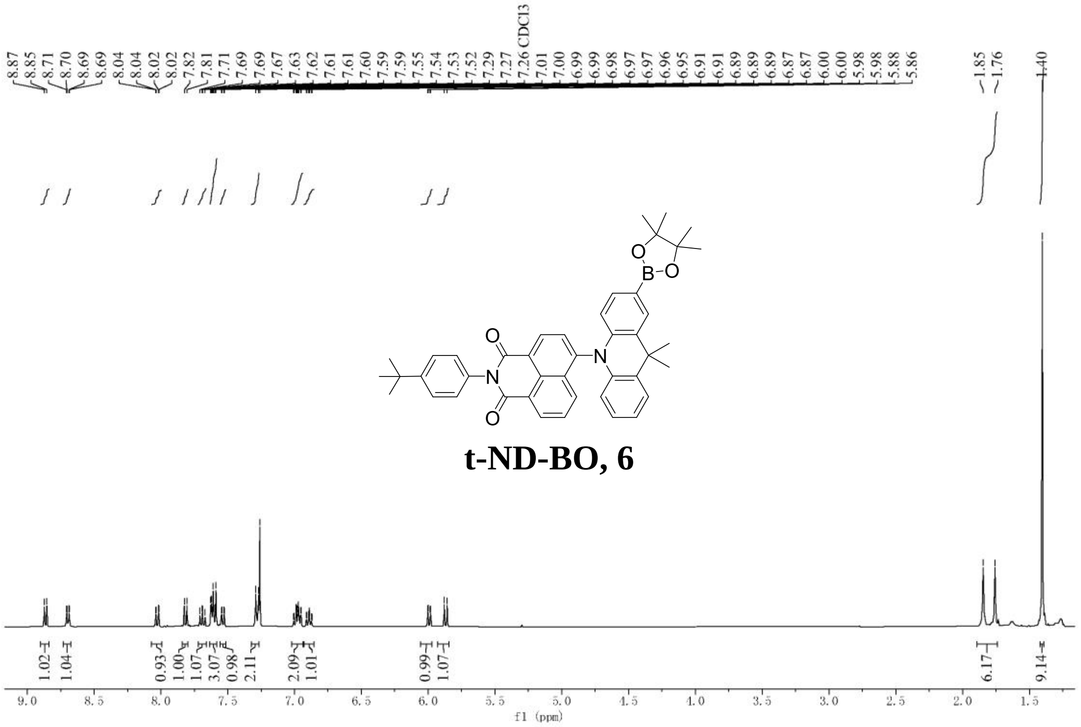

Supplementary Figure 13. ^{1}H NMR spectrum of 6 in chloroform-*d*.

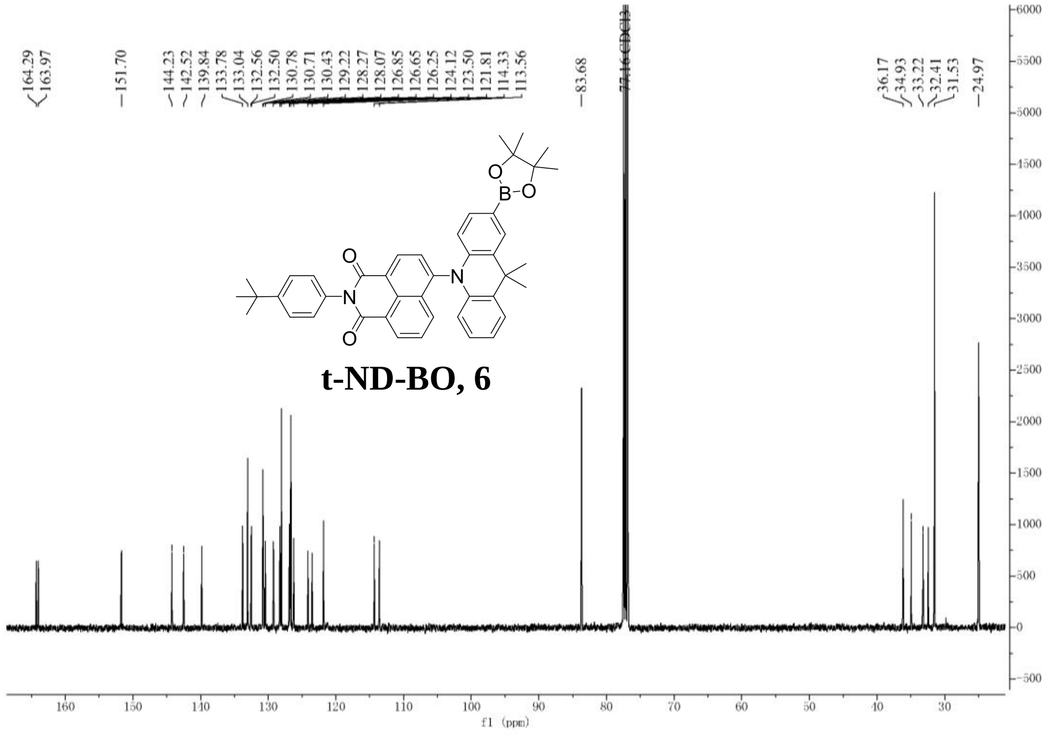

Supplementary Figure 14. ^{13}C NMR spectrum of 6 in chloroform-*d*.

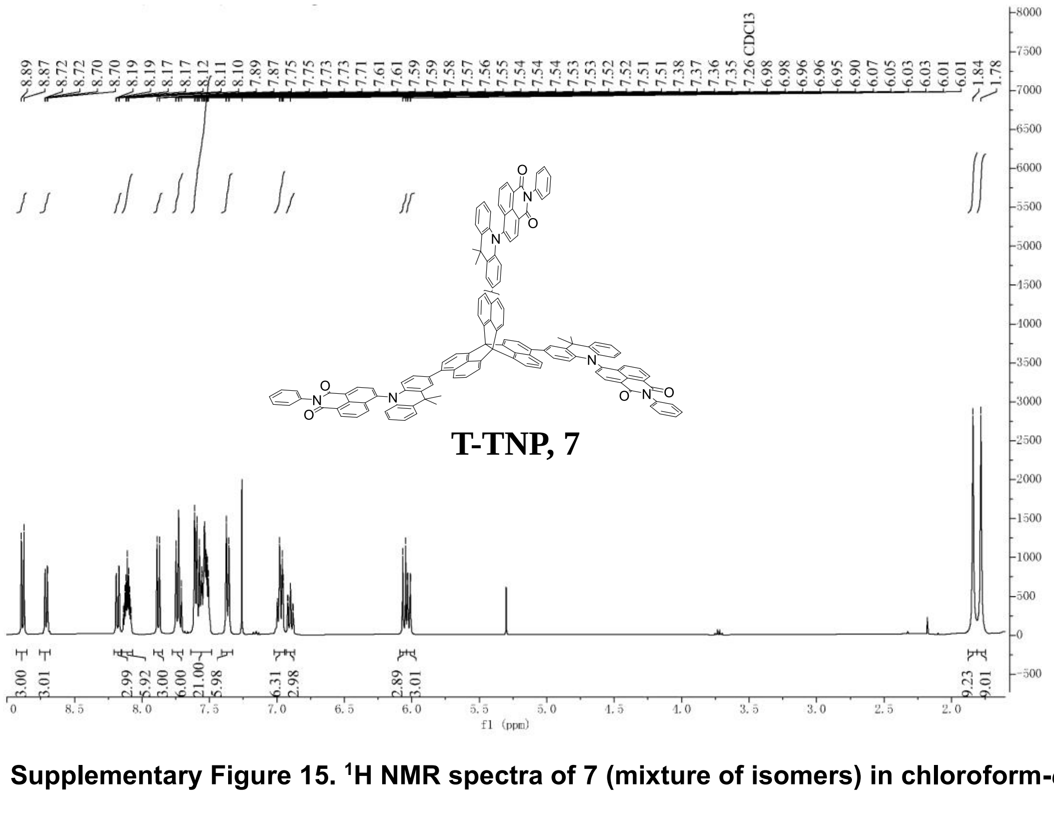

Supplementary Figure 15. ^{1}H NMR spectra of 7 (mixture of isomers) in chloroform-*d*.

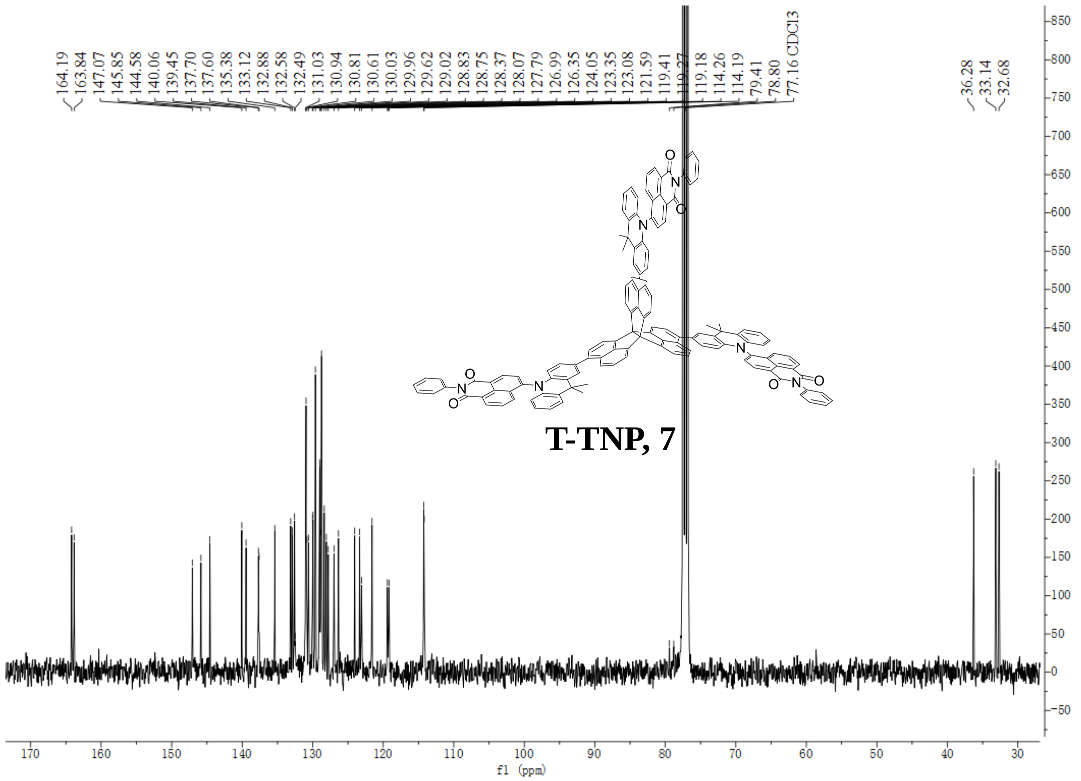

Supplementary Figure 16. **^{13}C NMR spectrum of 7 (mixture of isomers) in chloroform-*d*.**

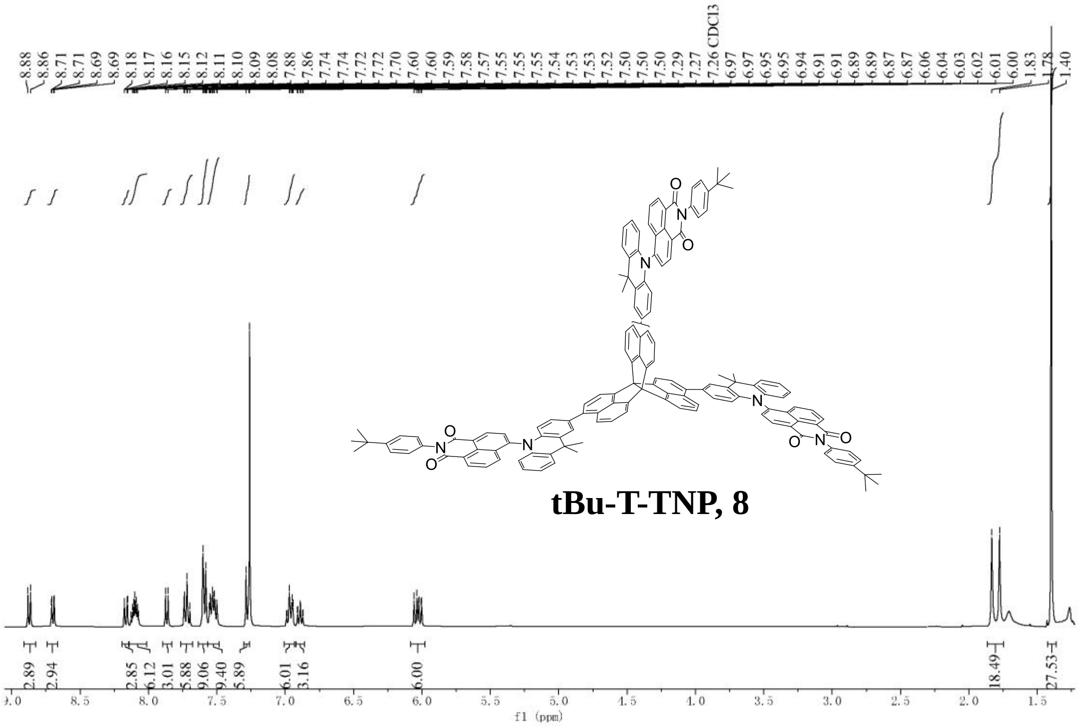

Supplementary Figure 17. **^{1}H NMR spectrum of 8 (mixture of isomers) in chloroform-*d*.**

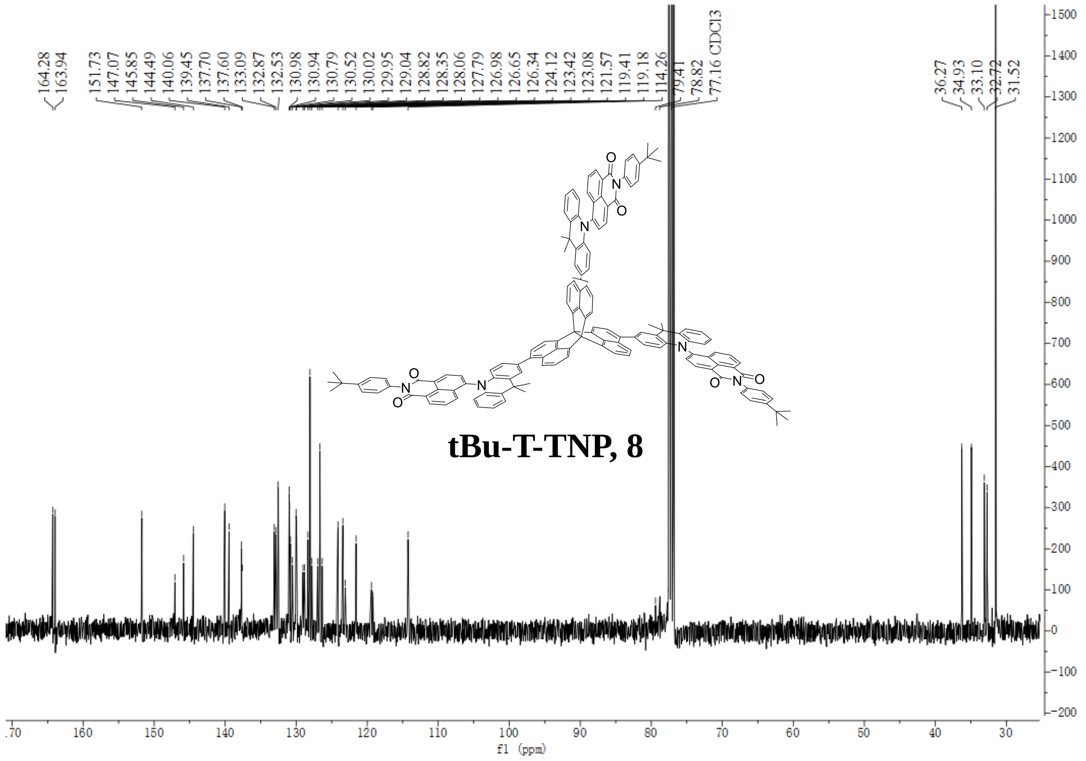

Supplementary Figure 18. ^{13}C NMR spectrum of 8 (mixture of isomers) in chloroform-*d*.

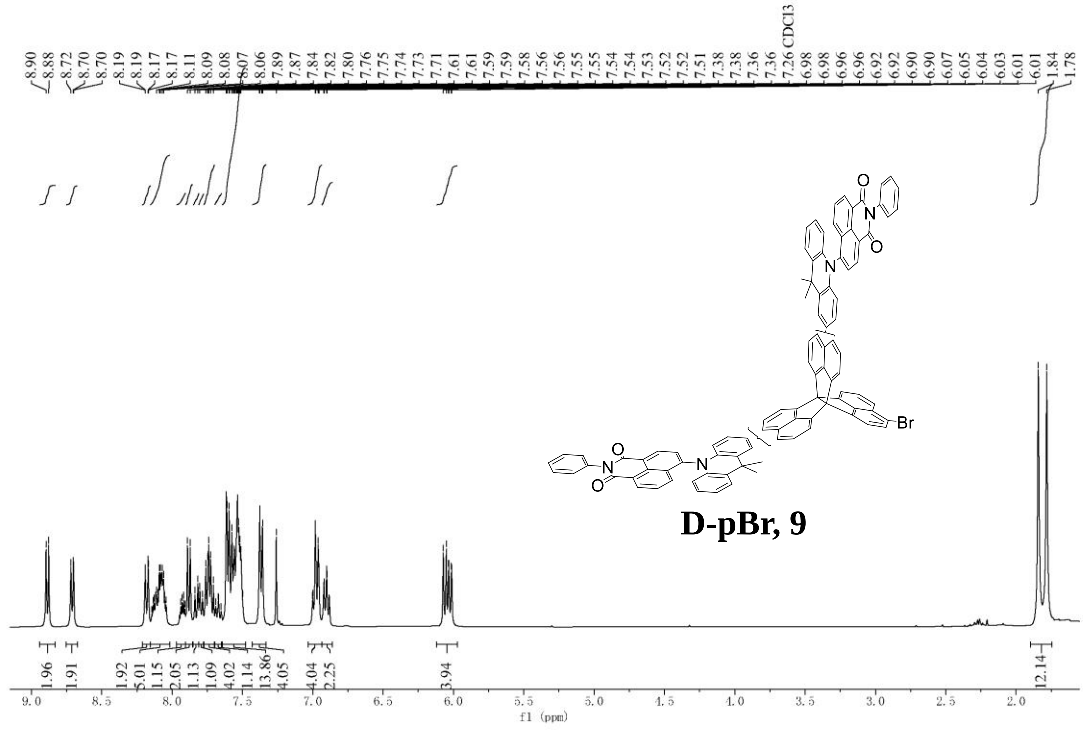

Supplementary Figure 19. ^{1}H NMR spectrum of 9 (mixture of isomers) in chloroform-*d*.

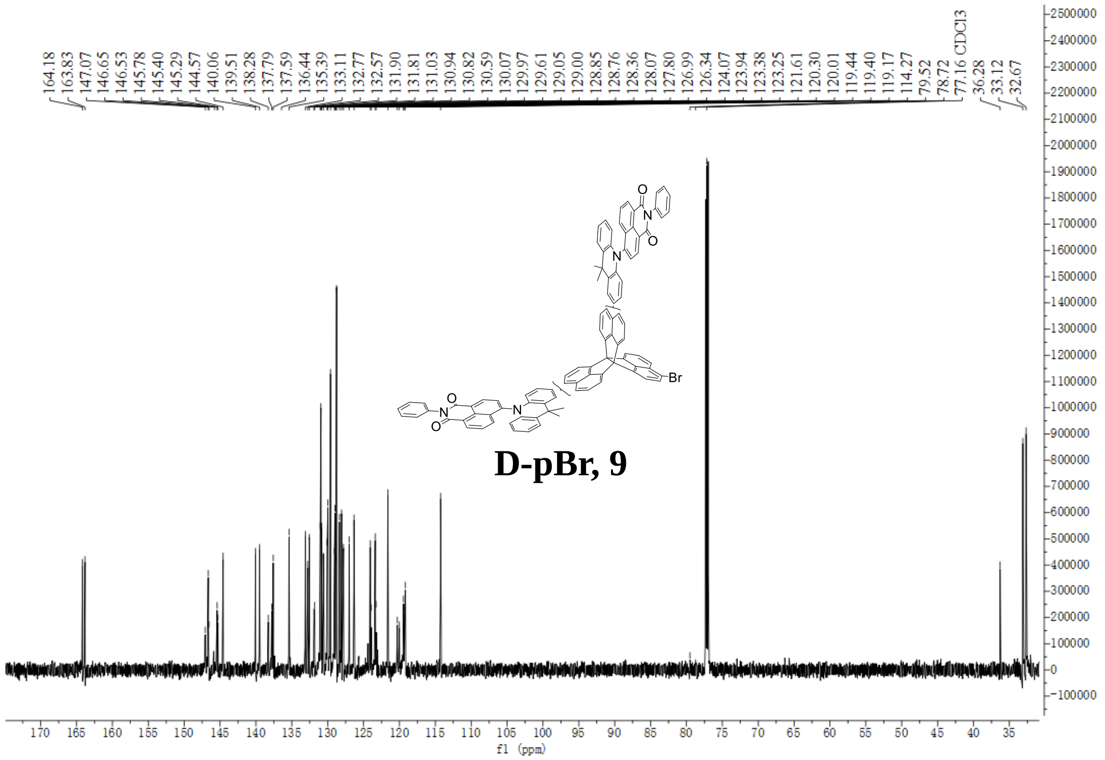

Supplementary Figure 20. ^{13}C NMR spectrum of 9 (mixture of isomers) in chloroform-*d*.

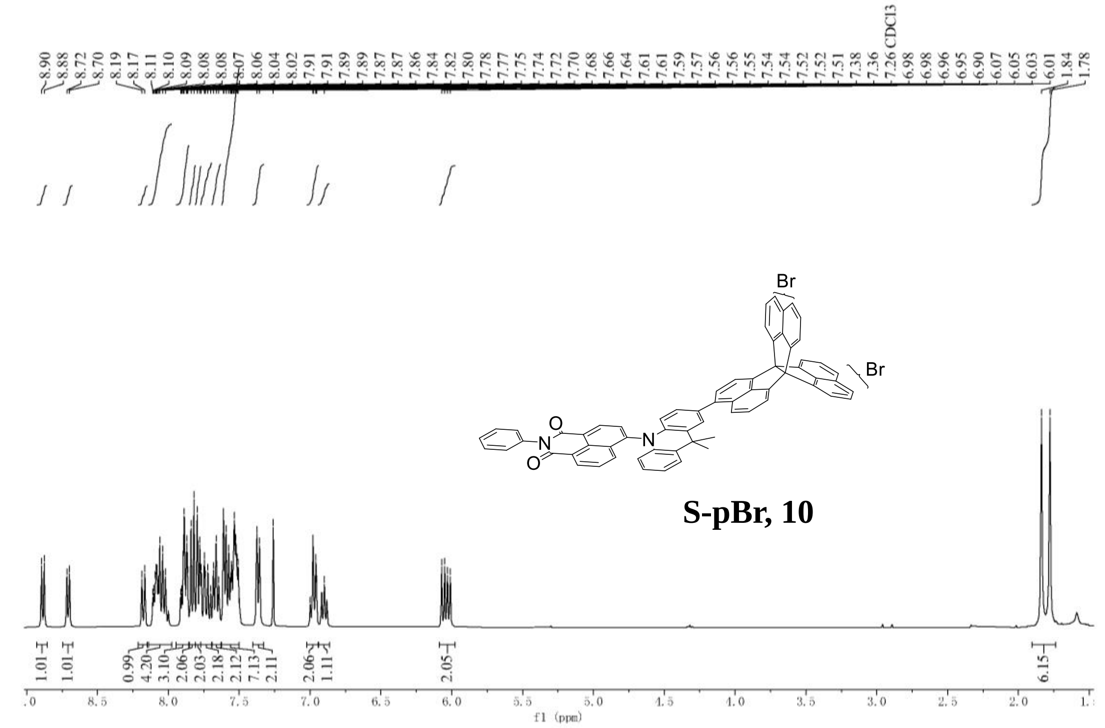

Supplementary Figure 21. ^{1}H NMR spectrum of 10 (mixture of isomers) in chloroform-*d*.

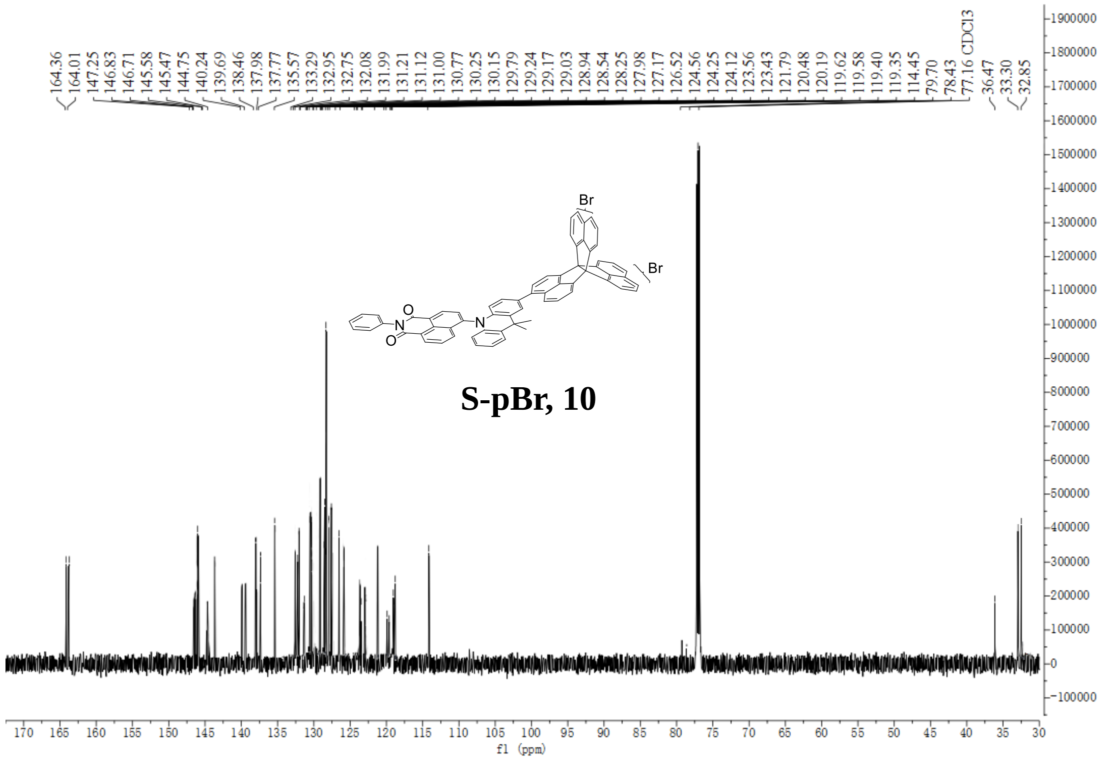

Supplementary Figure 22. ^{13}C NMR spectrum of 10 (mixture of isomers) in chloroform-*d*.

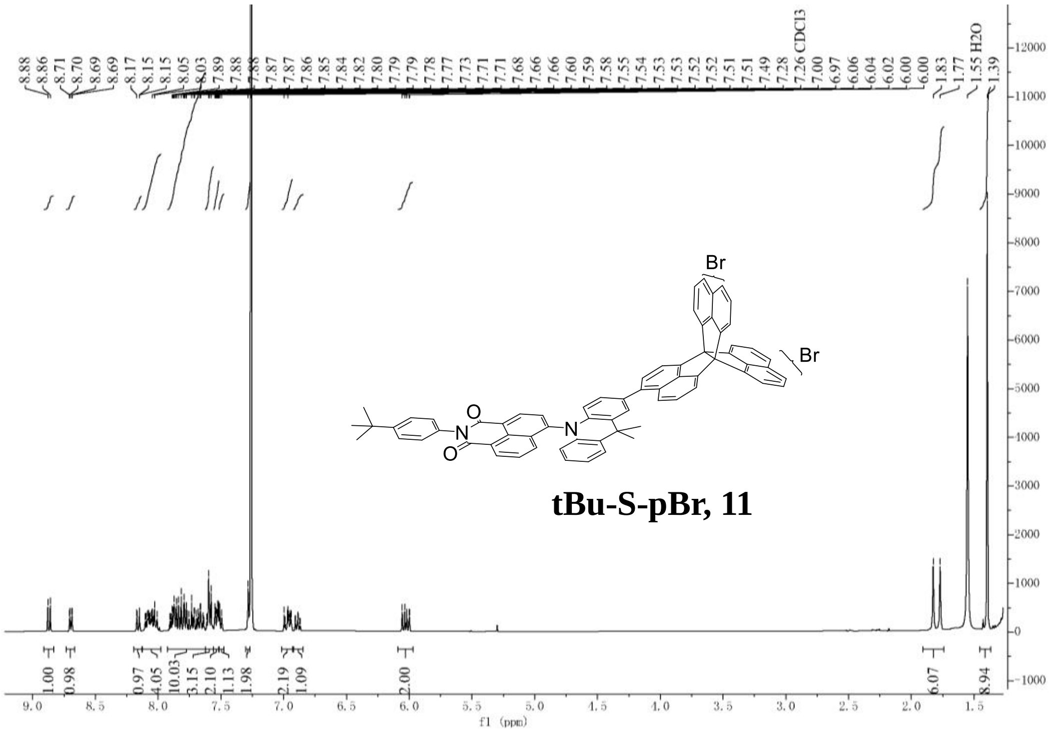

Supplementary Figure 23. ^{1}H NMR spectrum of 11 (mixture of isomers) in chloroform-*d*.

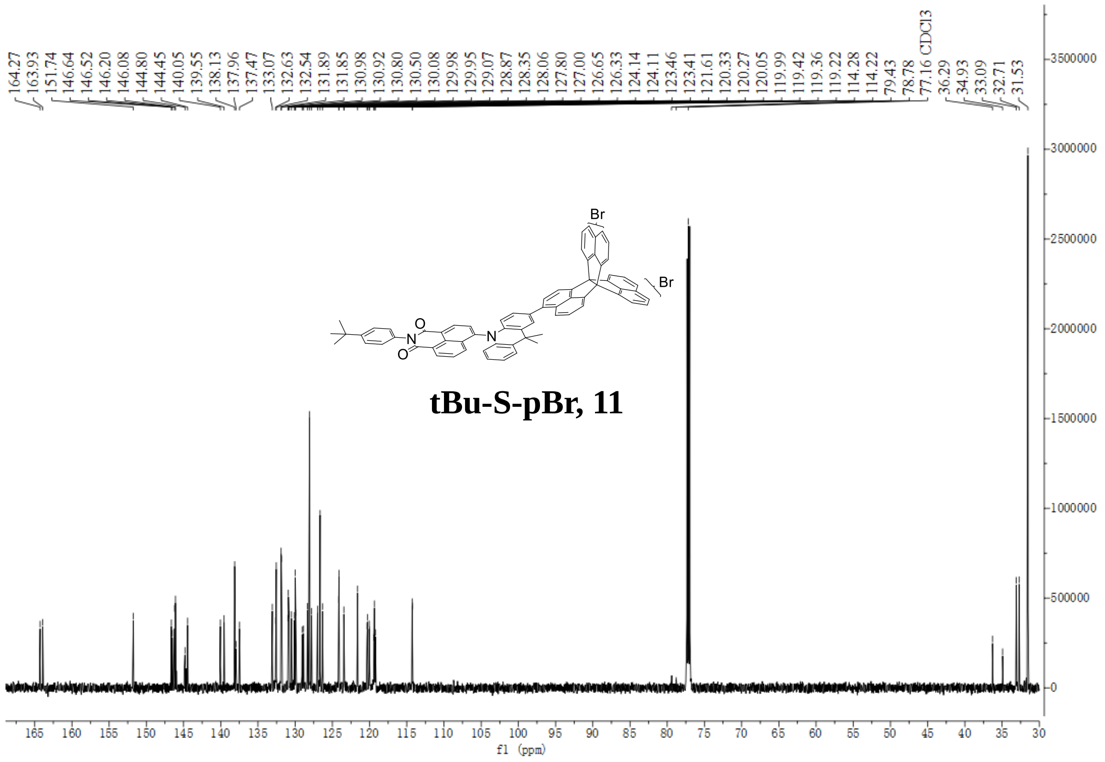

Supplementary Figure 24. ^{13}C NMR spectrum of 11 (mixture of isomers) in chloroform-*d*.

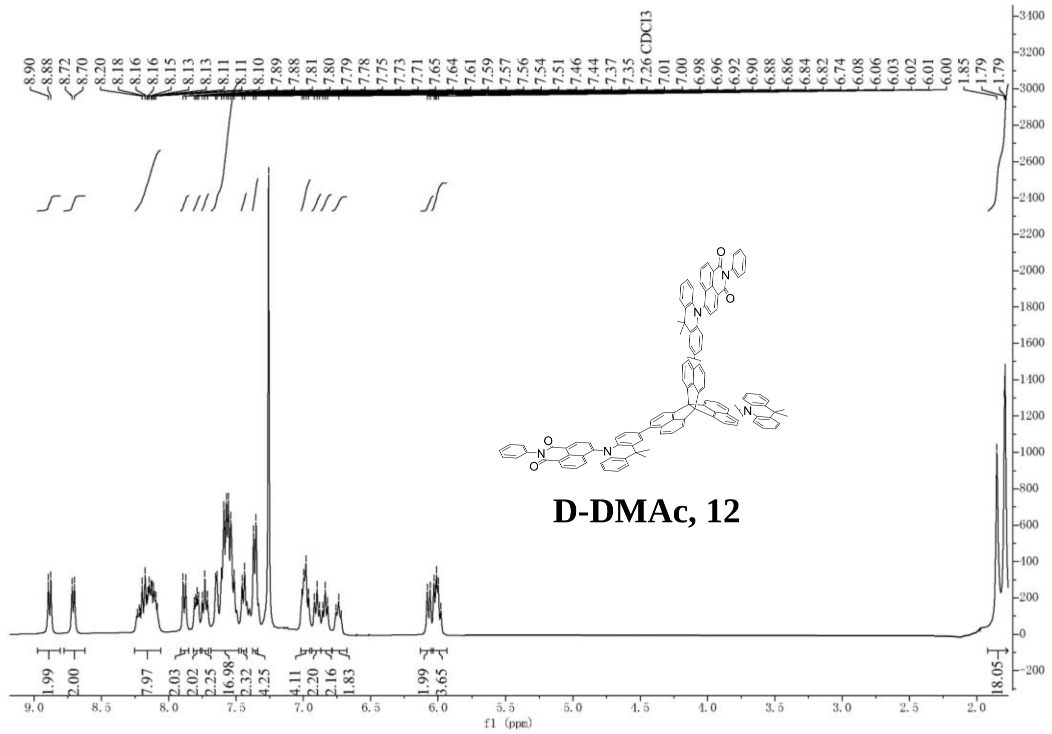

Supplementary Figure 25. ^{1}H NMR spectrum of 12 (mixture of isomers) in chloroform-*d*.

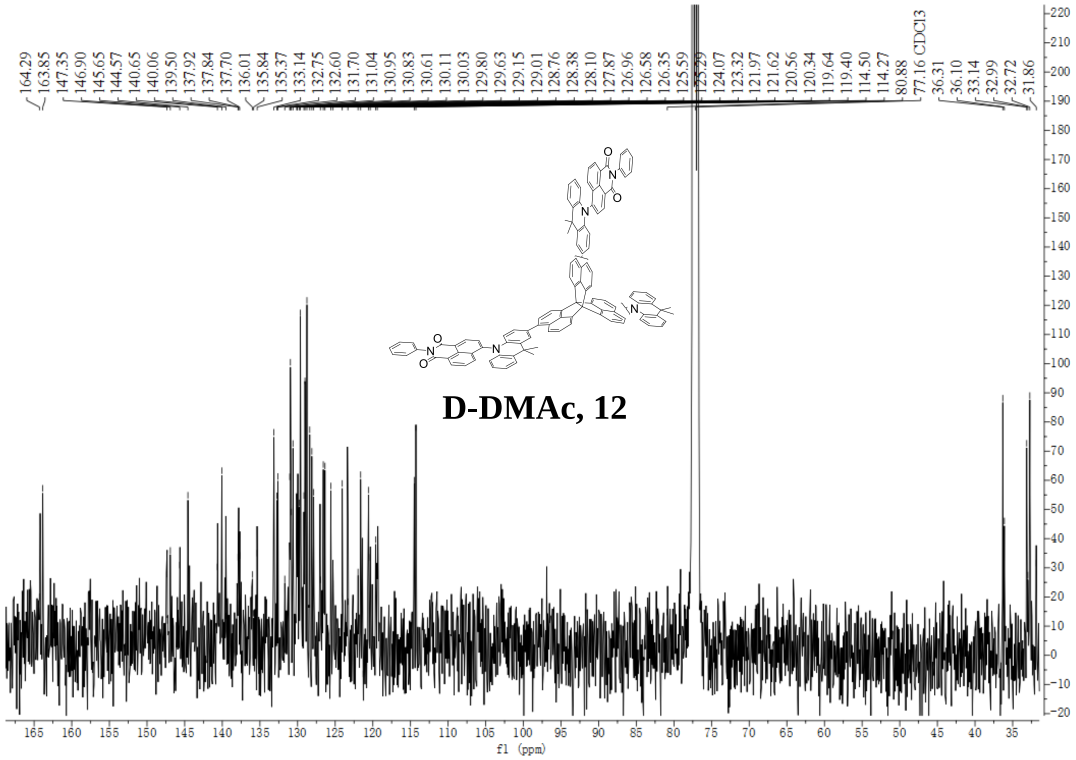

Supplementary Figure 26. ^{13}C **NMR spectrum of 12 (mixture of isomers) in chloroform-*d*.**

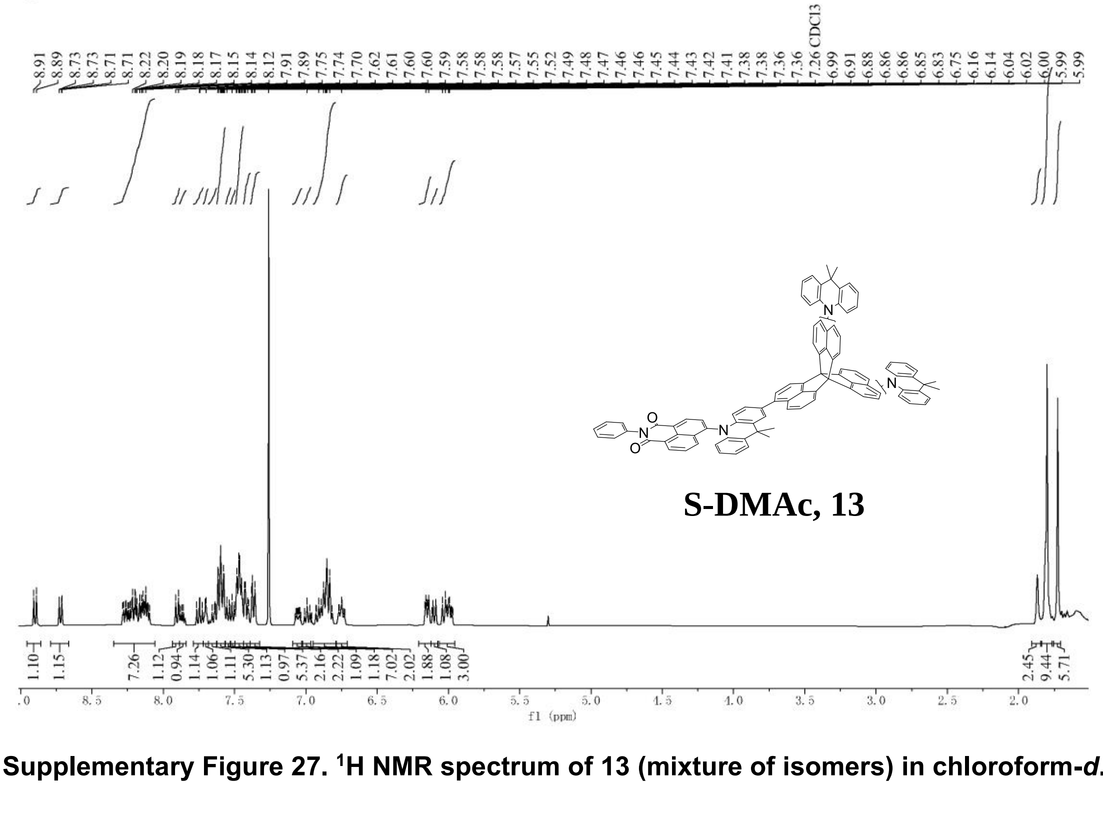

Supplementary Figure 27. ^{1}H **NMR spectrum of 13 (mixture of isomers) in chloroform-*d*.**

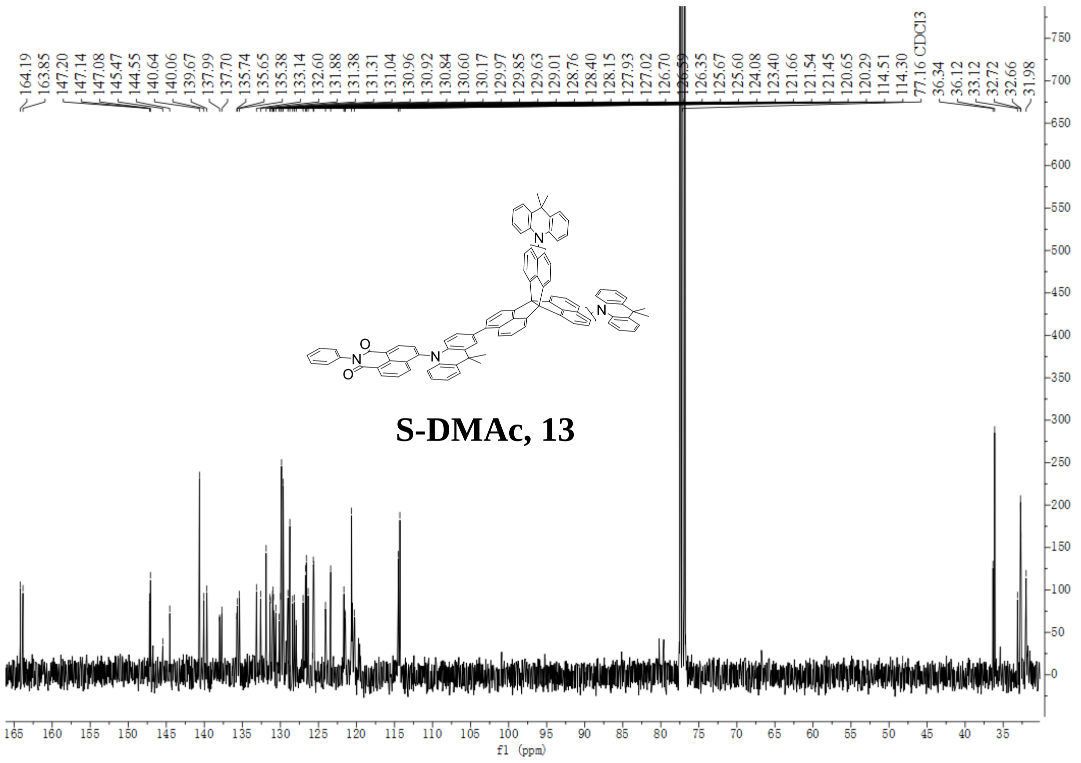

S-DMAc, 13

Supplementary Figure 28. ^{13}C NMR spectrum of 13 (mixture of isomers) in chloroform-*d*.

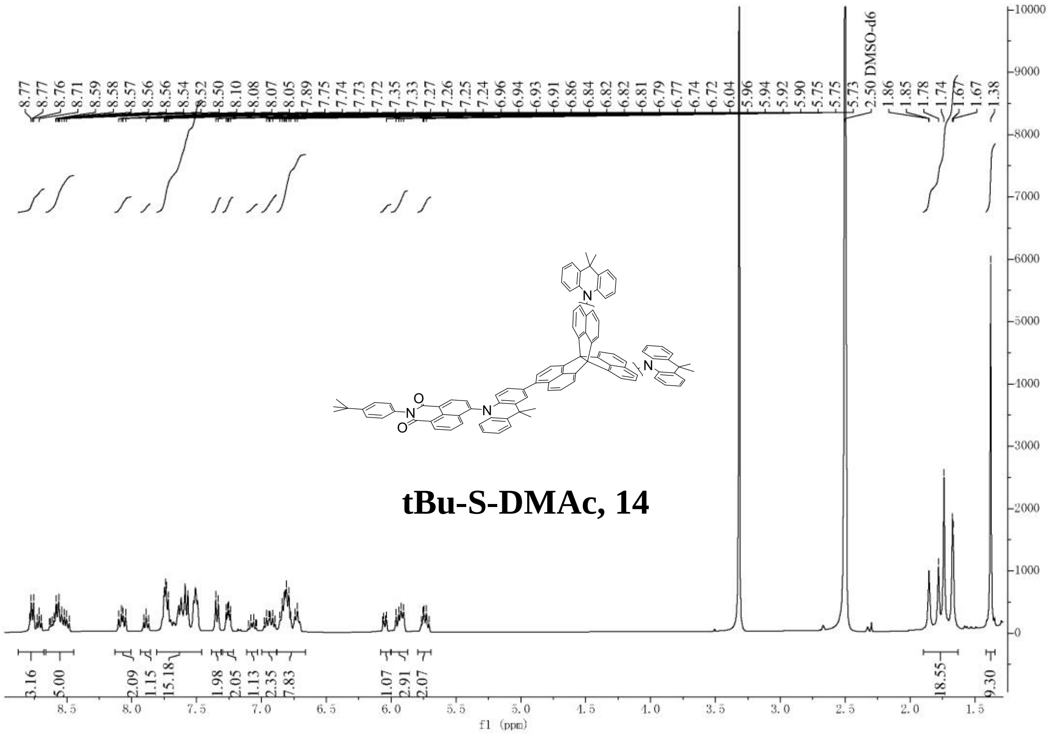

tBu-S-DMAc, 14

Supplementary Figure 29. ^{1}H NMR spectrum of 14 (mixture of isomers) in chloroform-*d*.

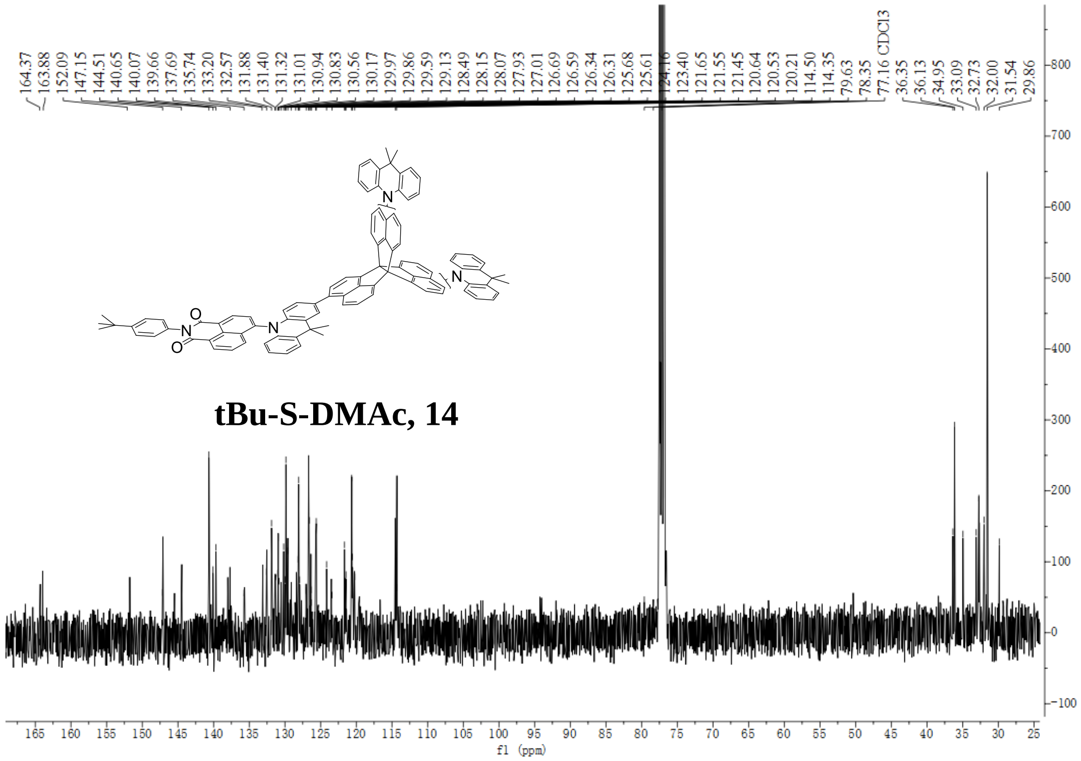

Supplementary Figure 30. ^{13}C NMR spectrum of 14 (mixture of isomers) in chloroform-*d*.

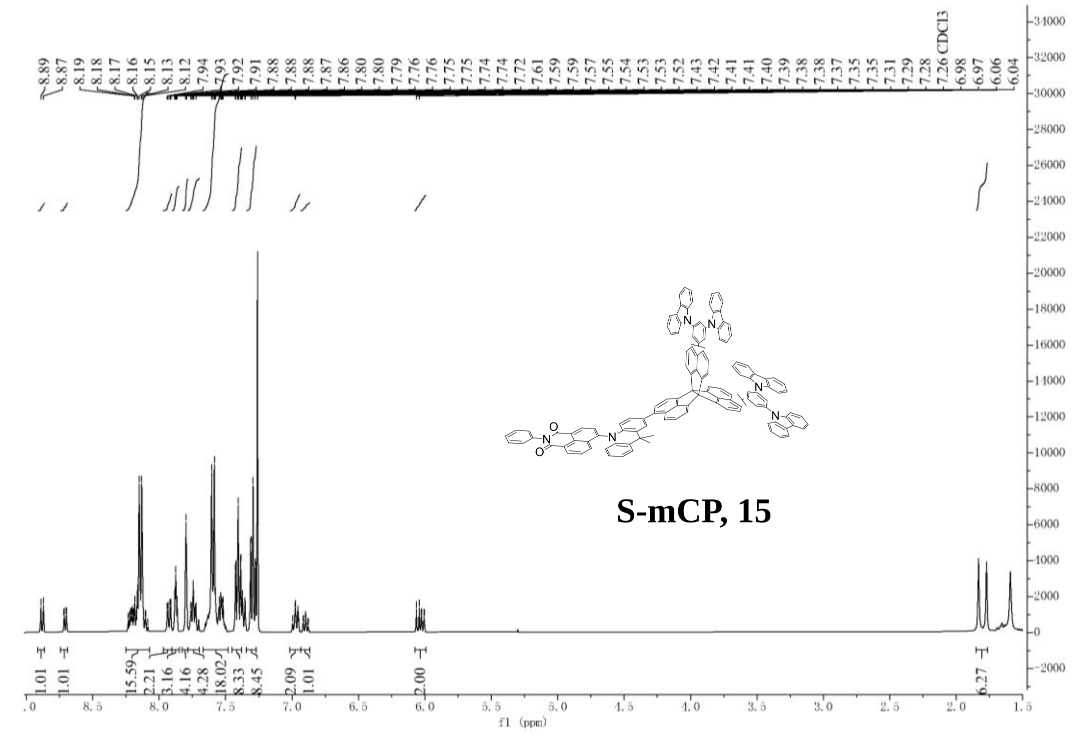

Supplementary Figure 31. ^{1}H NMR spectrum of 15 (mixture of isomers) in chloroform-*d*.

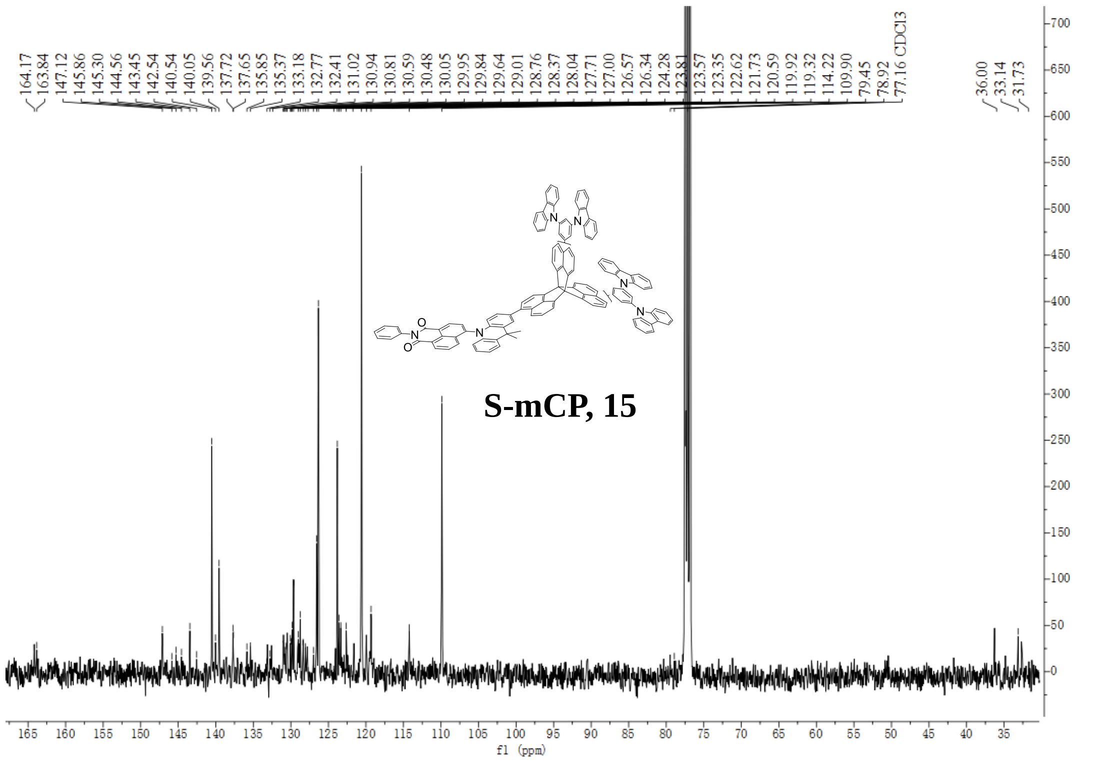

Supplementary Figure 32. ^{13}**C NMR spectrum of 15 (mixture of isomers) in chloroform-*d*.**

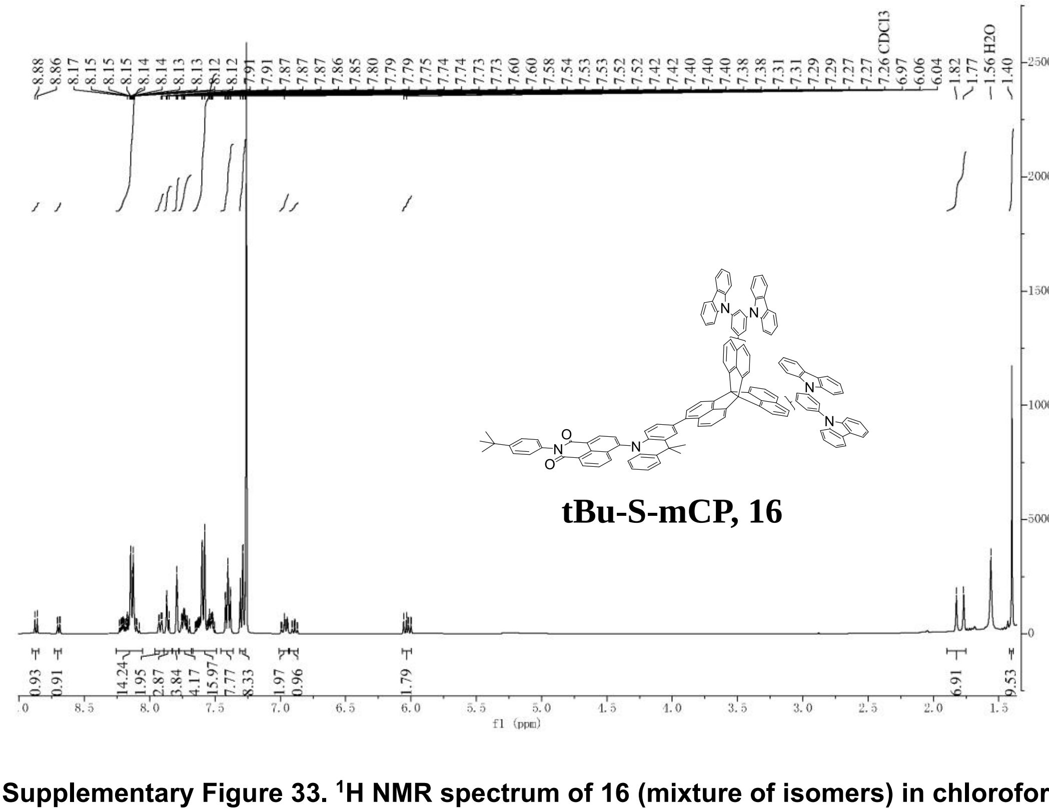

Supplementary Figure 33. ^{1}**H NMR spectrum of 16 (mixture of isomers) in chloroform-*d*.**

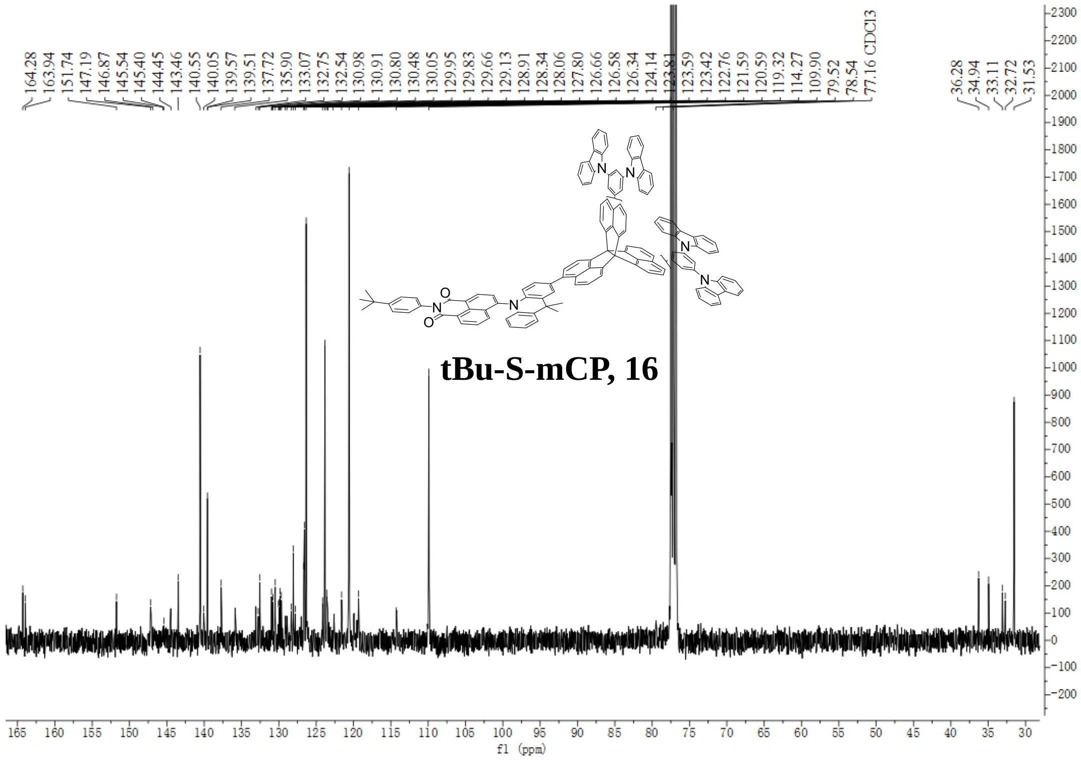

Supplementary Figure 34. ^{13}C NMR spectrum of 16 (mixture of isomers) in chloroform-*d*.

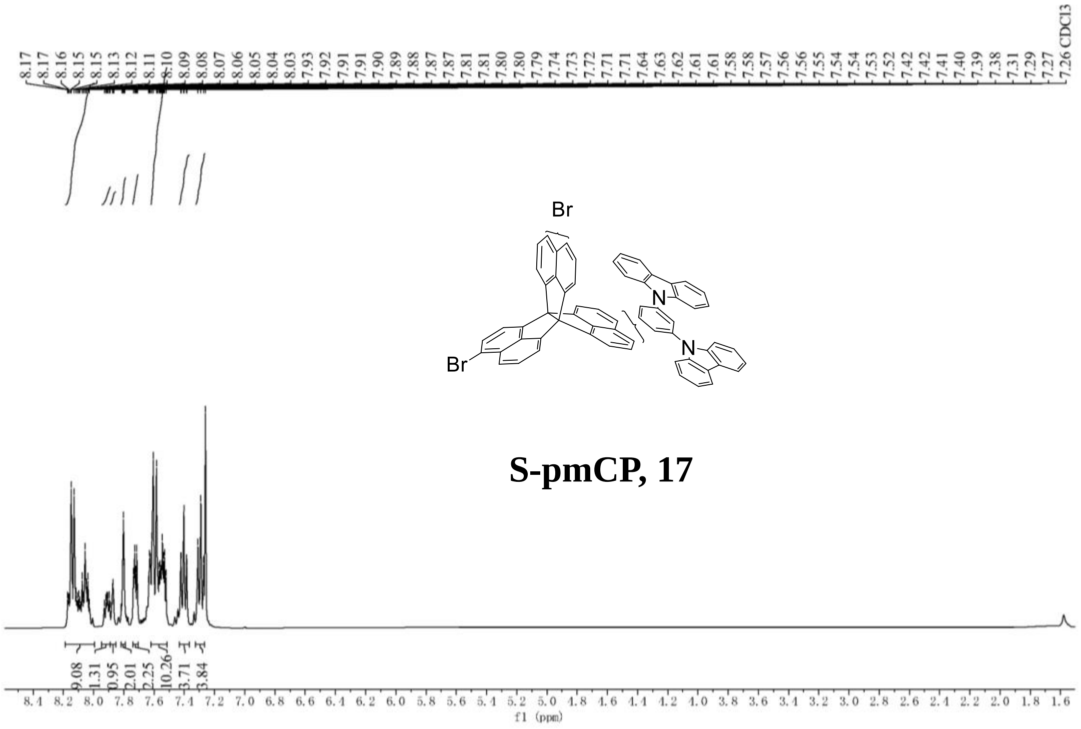

Supplementary Figure 35. ^{1}H NMR spectrum of 17 (mixture of isomers) in chloroform-*d*.

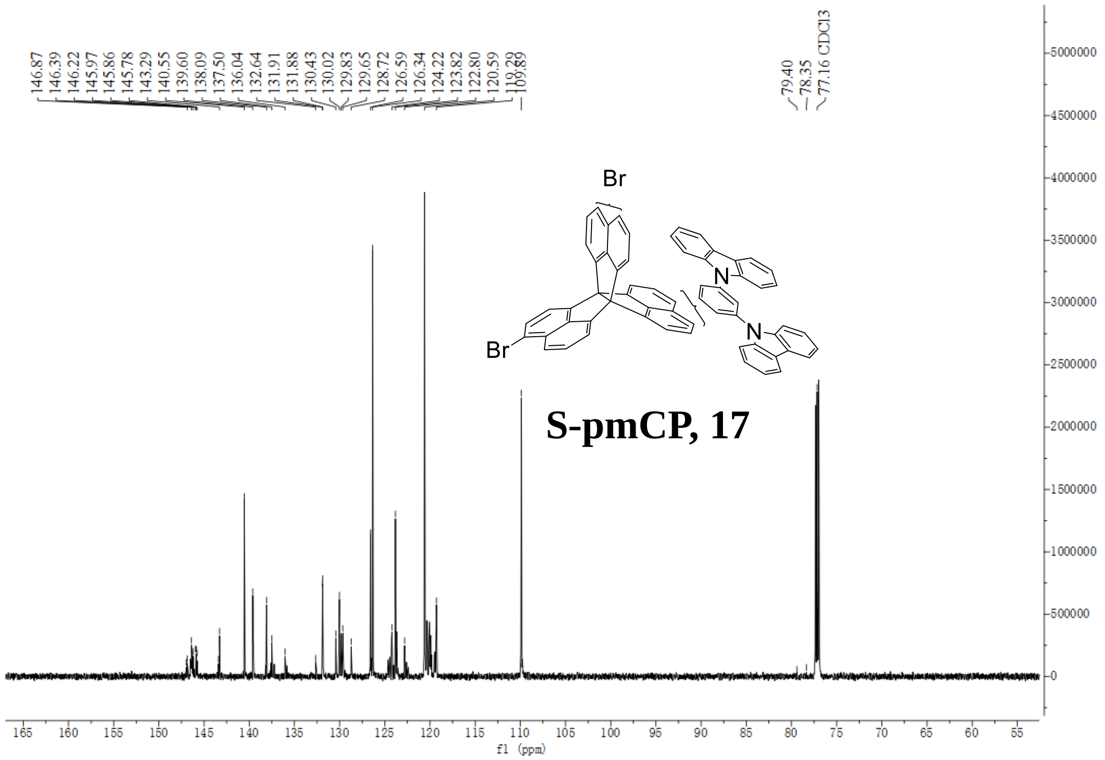

Supplementary Figure 36. ^{13}C NMR spectrum of 17 (mixture of isomers) in chloroform-*d*.

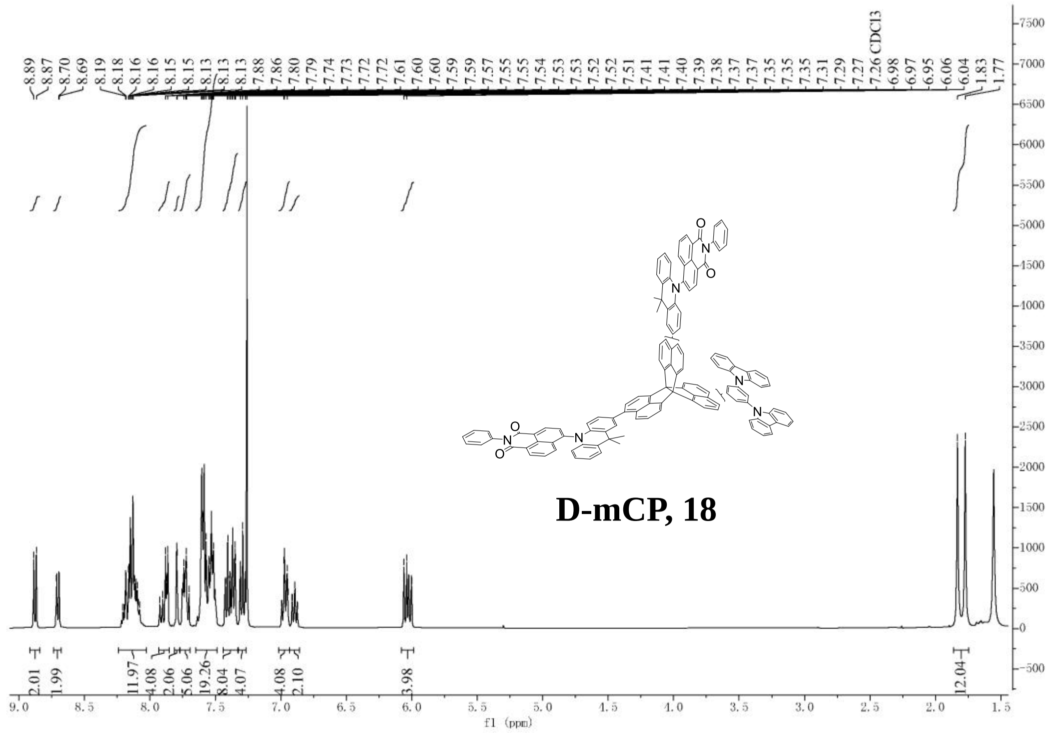

Supplementary Figure 37. ^{1}H NMR spectrum of 18 (mixture of isomers) in chloroform-*d*.

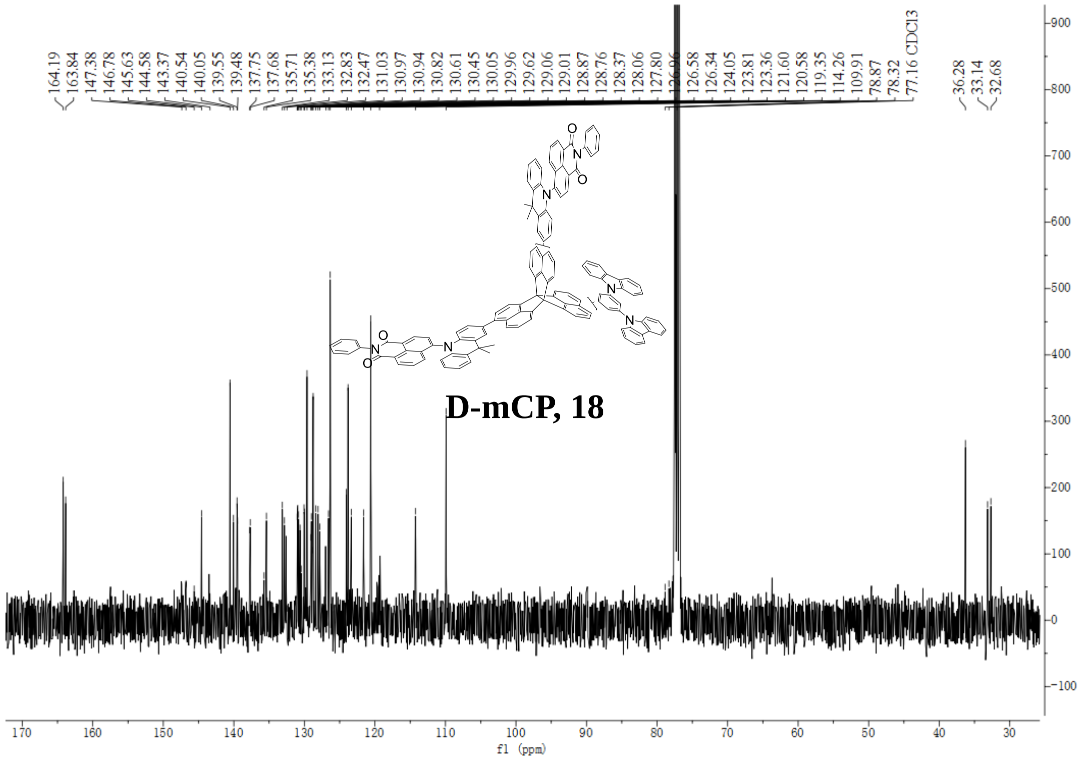

Supplementary Figure 38. ^{13}C NMR spectrum of 18 (mixture of isomers) in chloroform-*d*.

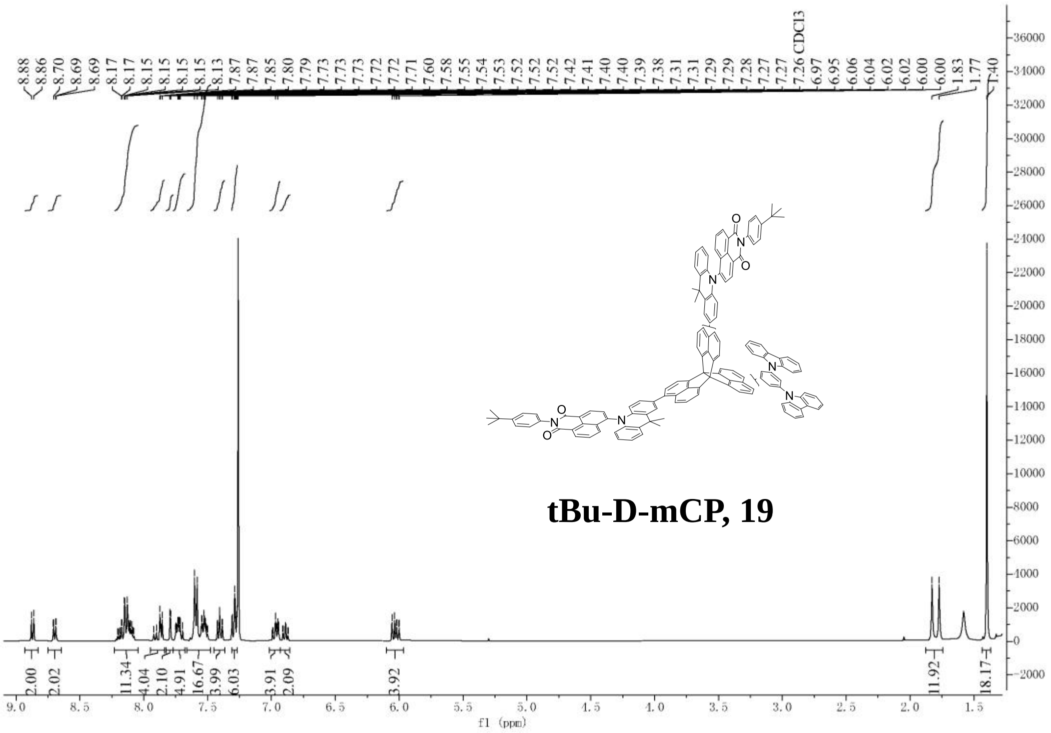

Supplementary Figure 39. ^{1}H NMR spectrum of 19 (mixture of isomers) in chloroform-*d*.

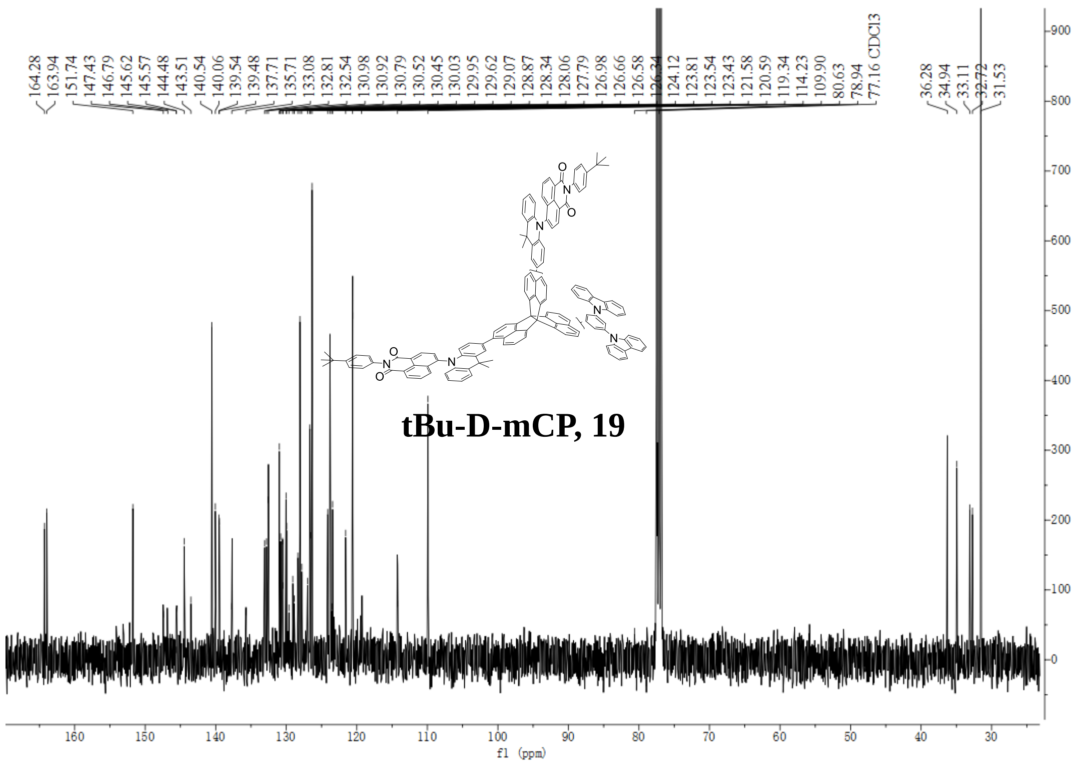

Supplementary Figure 40. ^{13}C NMR spectrum of 19 (mixture of isomers) in chloroform-*d*.

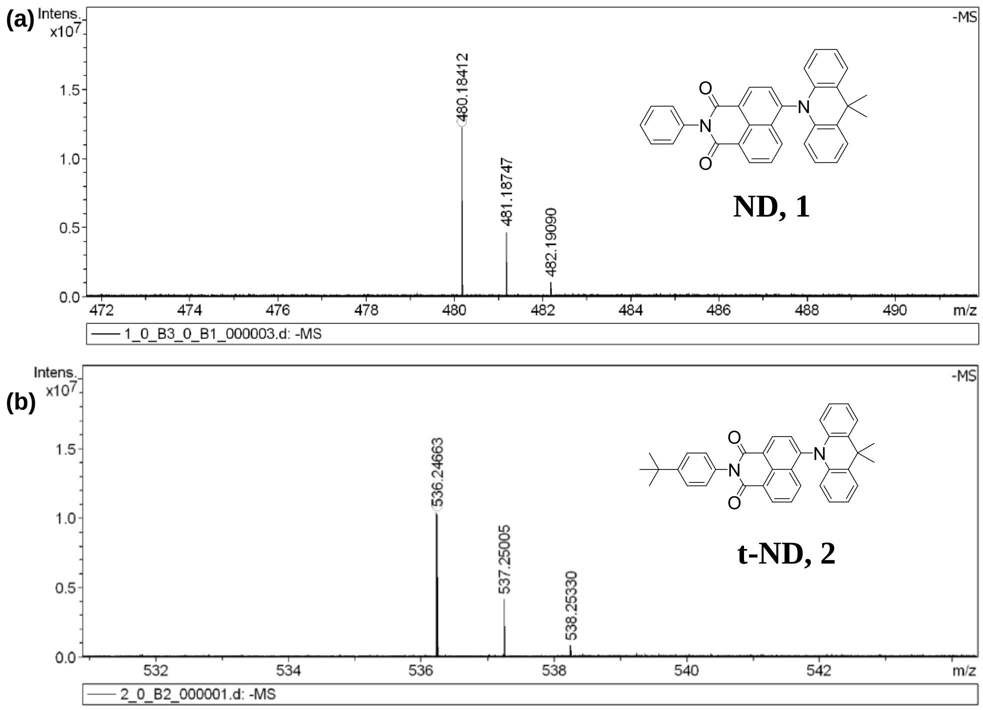

Supplementary Figure 41. High-resolution mass spectra of 1(a) and 2(b)

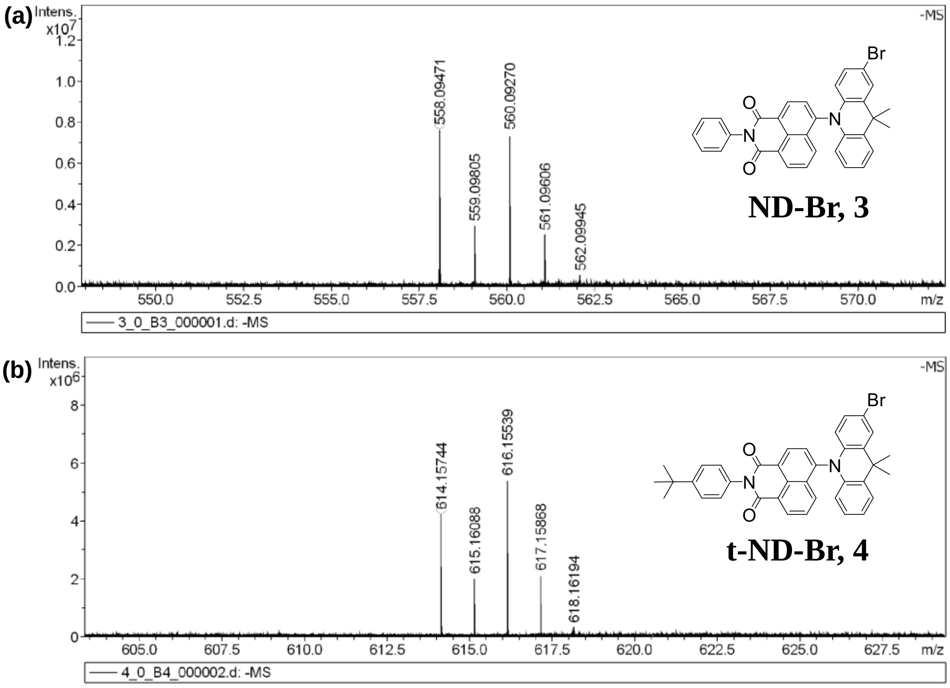

Supplementary Figure 42. High-resolution mass spectra of 3(a) and 4(b)

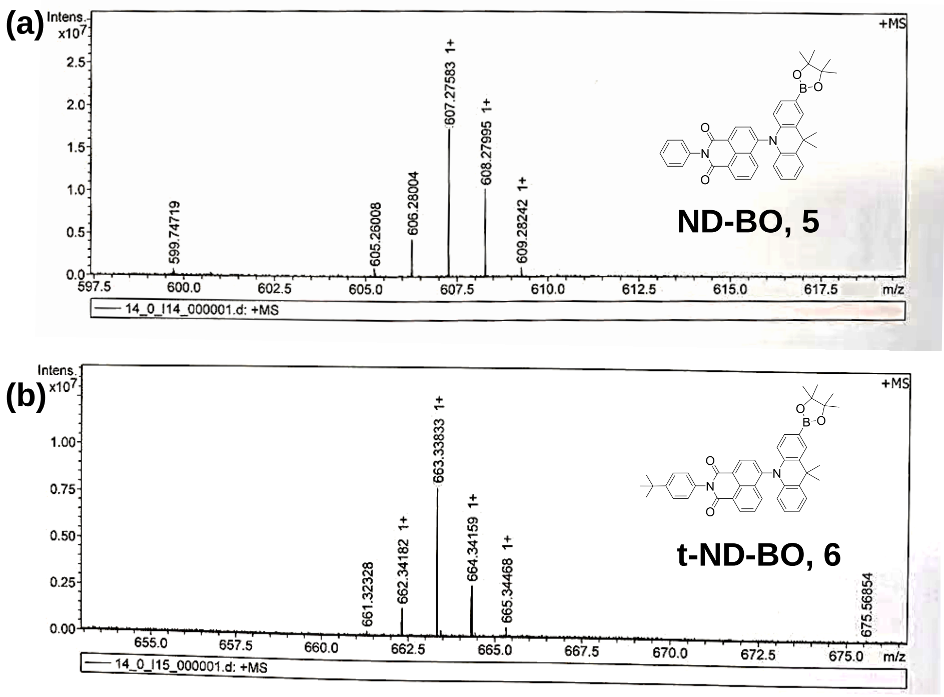

Supplementary Figure 43. High-resolution mass spectra of 5(a) and 6(b)

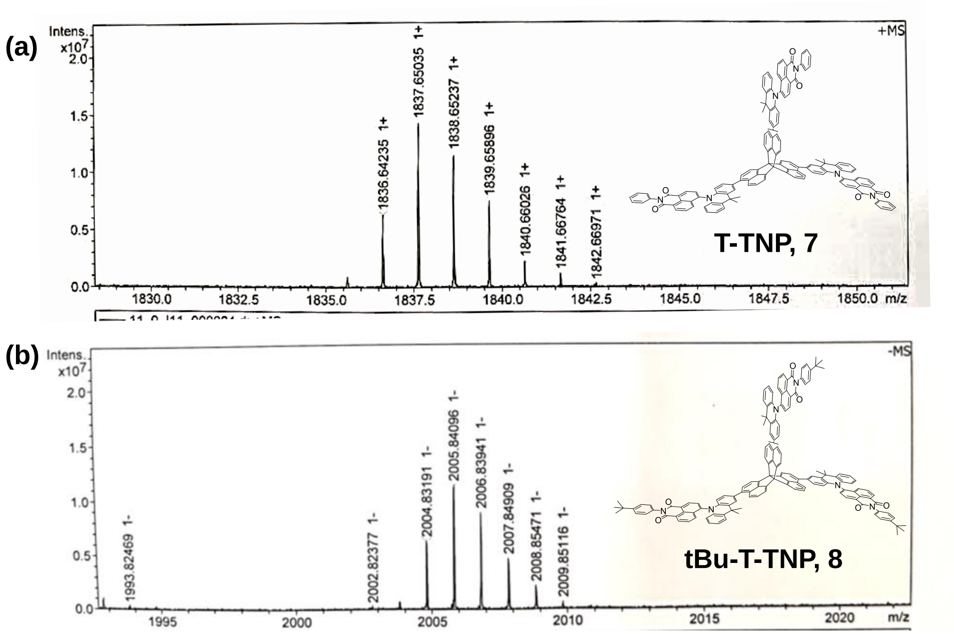

Supplementary Figure 44. High-resolution mass spectra of 7(a) and 8(b)

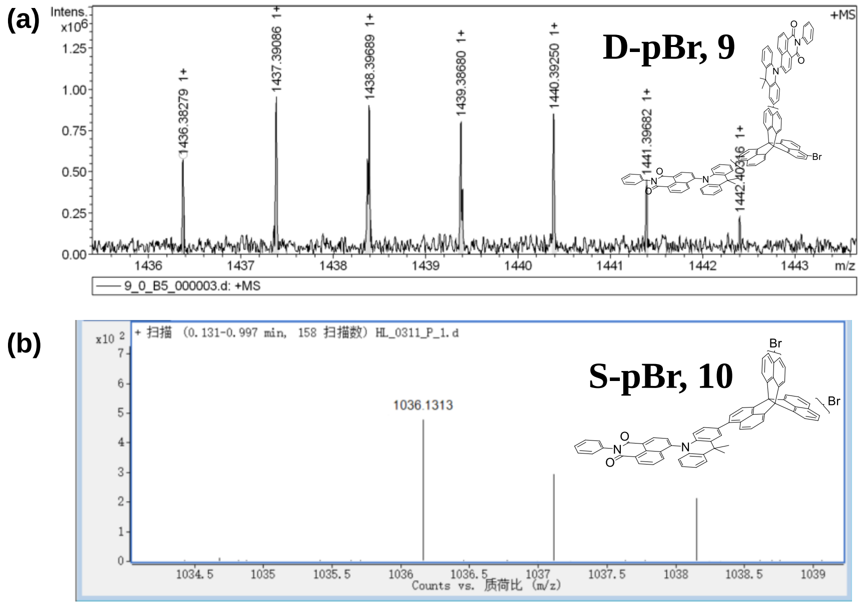

Supplementary Figure 45. High-resolution mass spectra of 9(a) and 10(b)

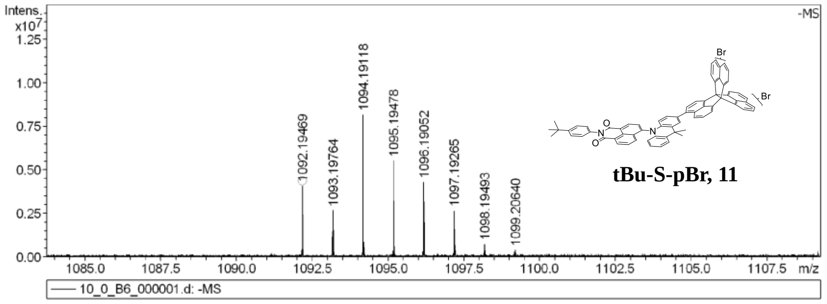

Supplementary Figure 46. High-resolution mass spectra of 11

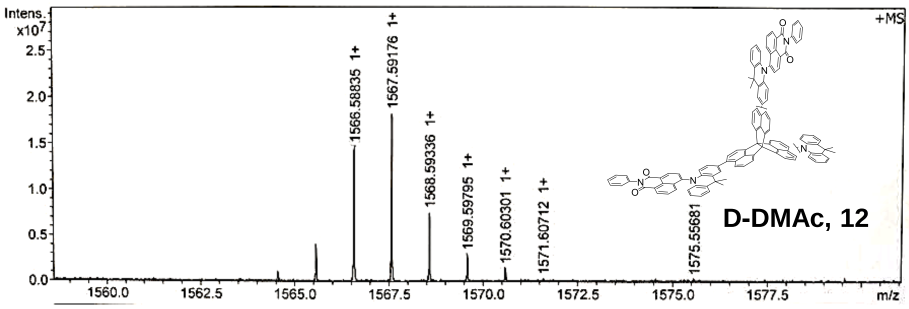

Supplementary Figure 47. High-resolution mass spectrum of 12

(a)

(b)

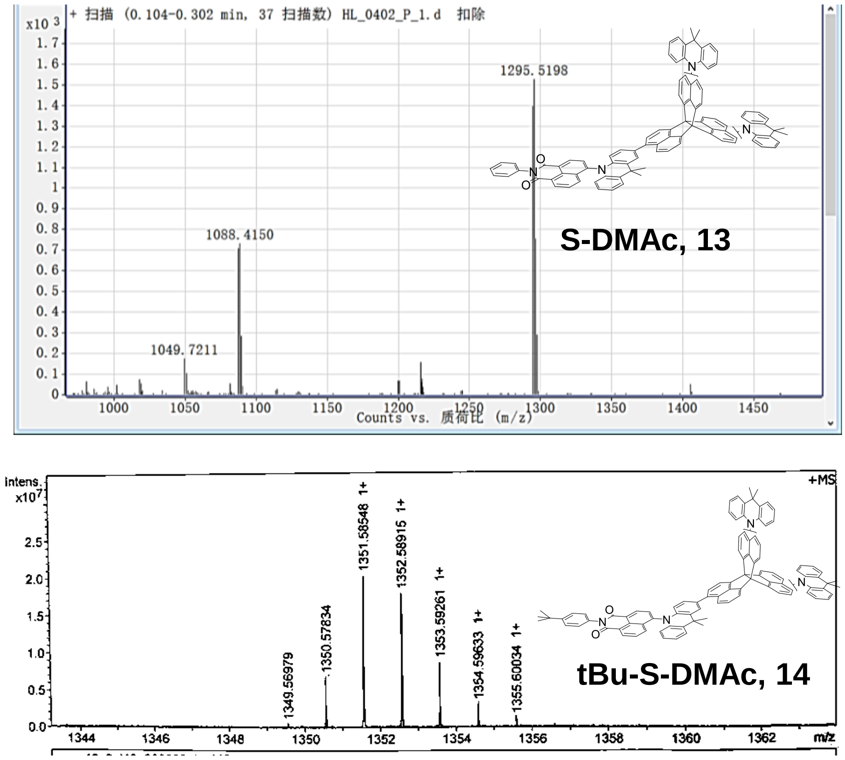

Supplementary Figure 48. High-resolution mass spectra of 13(a) and 14(b)

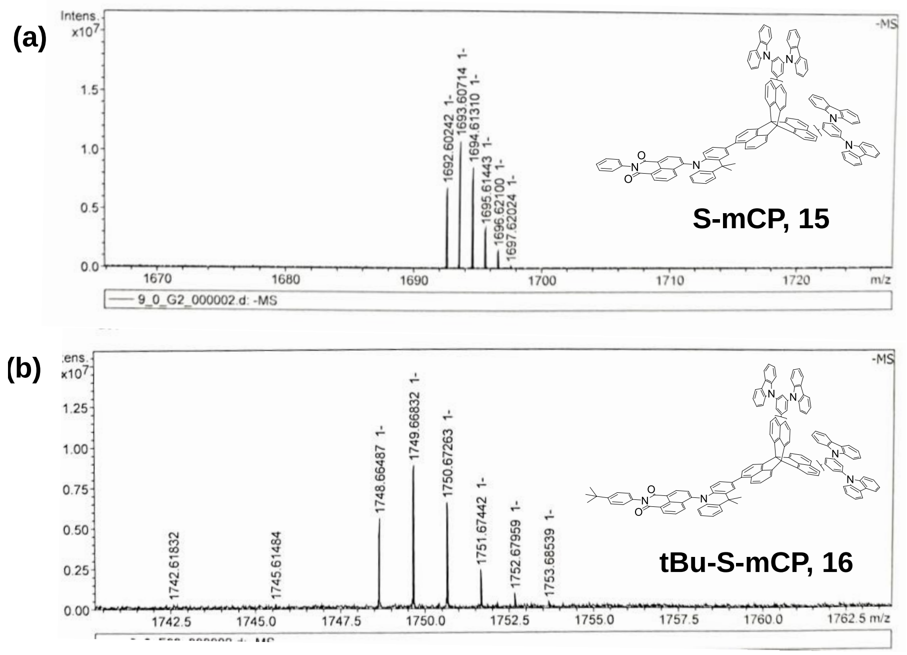

Supplementary Figure 49. High-resolution mass spectra of 15(a) and 16(b)

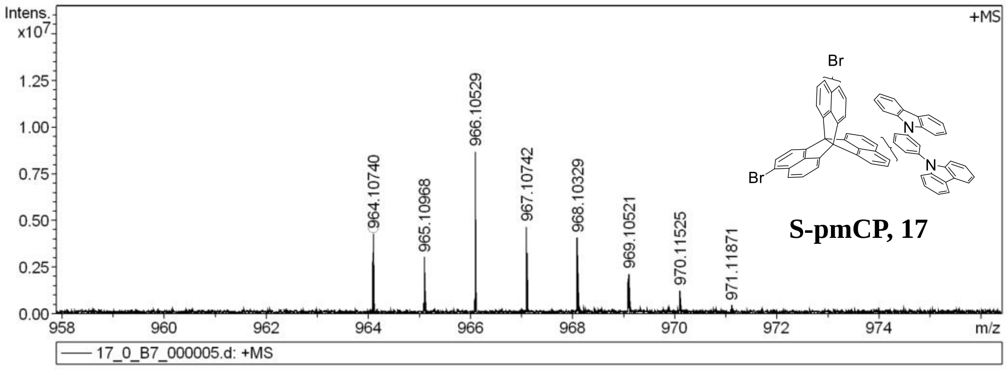

Supplementary Figure 50. High-resolution mass spectra of 17

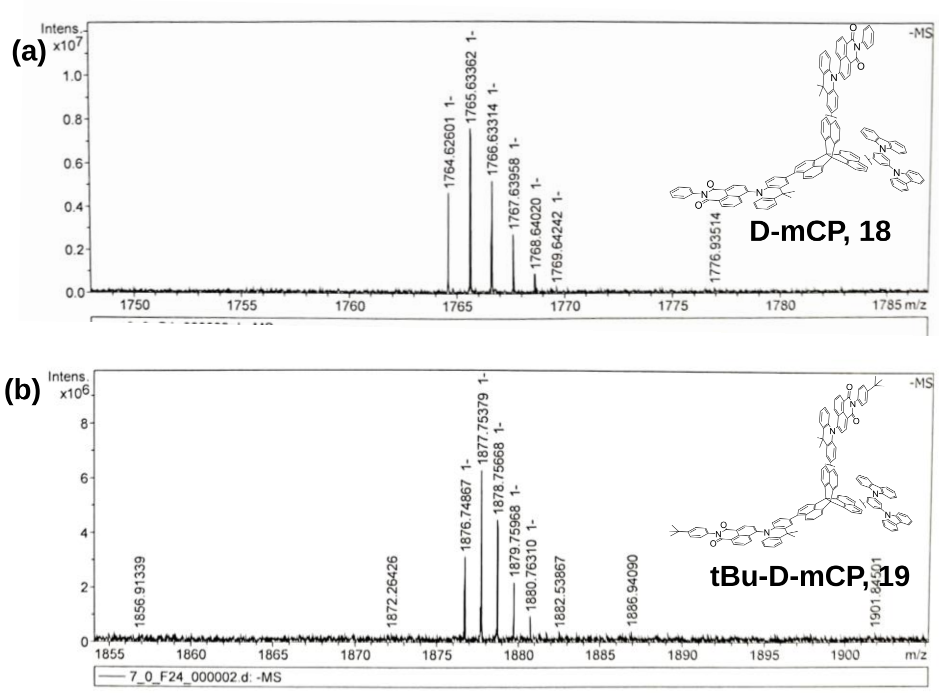

Supplementary Figure 51. High-resolution mass spectra of 18(a) and 19(b)

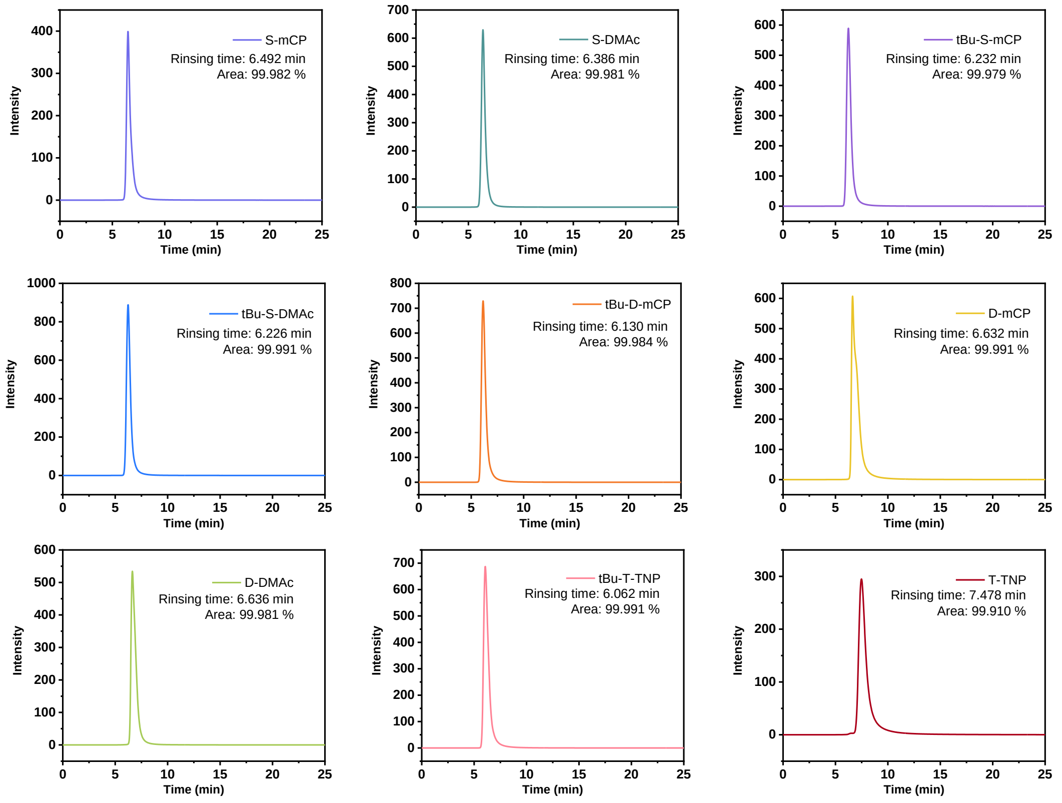

Supplementary Figure 52. High performance liquid chromatography of nine emitters. The rising time and peak area are marked.

Supplementary Note 4: Supplementary figures

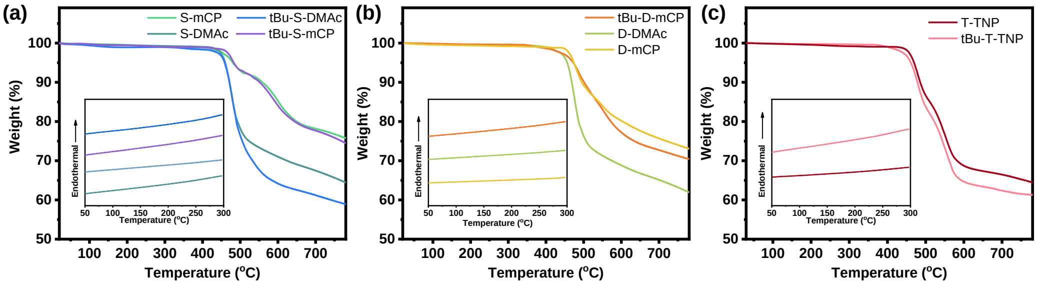

Supplementary Figure 53. Thermal properties characterization. Thermal gravimetric analysis of (a) Single-TADF series (S-DMAc, S-mCP, tBu-S-DMAc, tBu-S-mCP); (b) Double-TADF series (tBu-D-mCP; D-DMAc; D-mCP); (c) Triple-TADF series (tBu-T-TNP, T-TNP). The insets are the differential scanning calorimetry characterizations of the emitters.

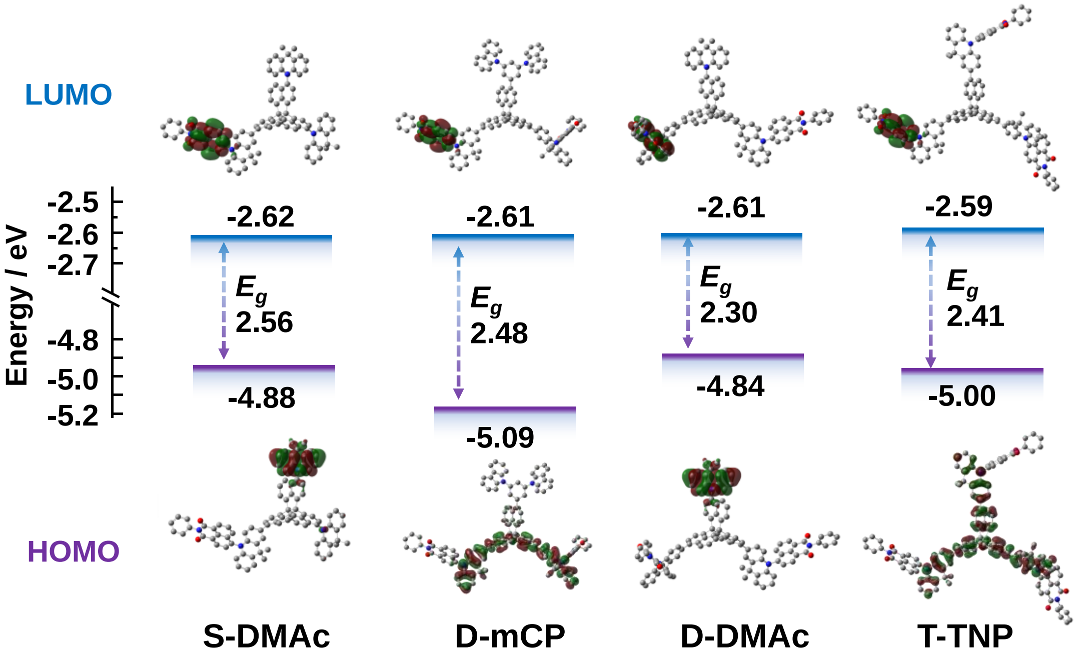

Supplementary Figure 54. Frontier molecular orbital simulation. The distributions of the highest occupied molecular orbital (HOMO) and lowest unoccupied molecular orbital (LUMO) with their energy gaps (E_gs) of four emitters were calculated by density functional theory (DFT) simulation with the B3LYP 6-31G(d) level.

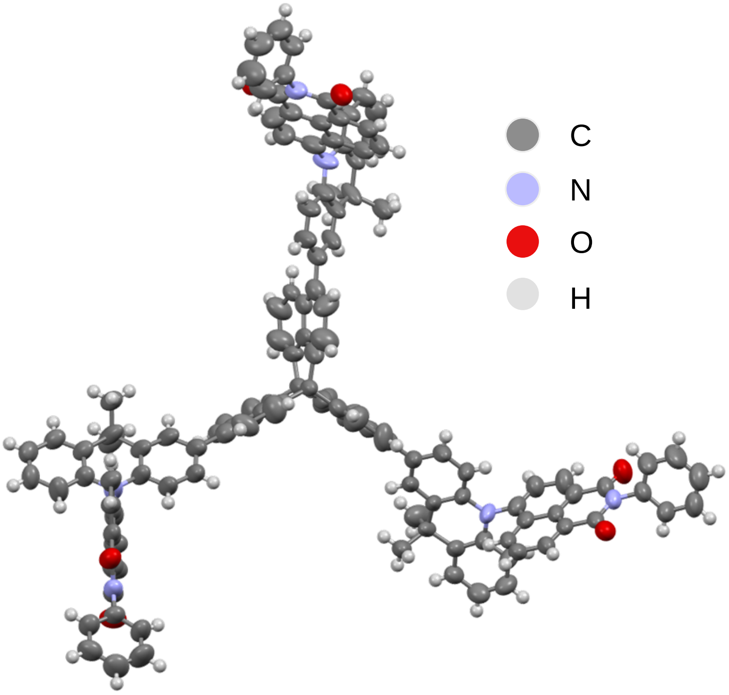

Supplementary Figure 55. Single crystal data of T-TNP. Single-crystal structure with C, N, O atoms in the ellipsoid model shown at a 50% level and H atoms in a ball-stick mode for clarity.

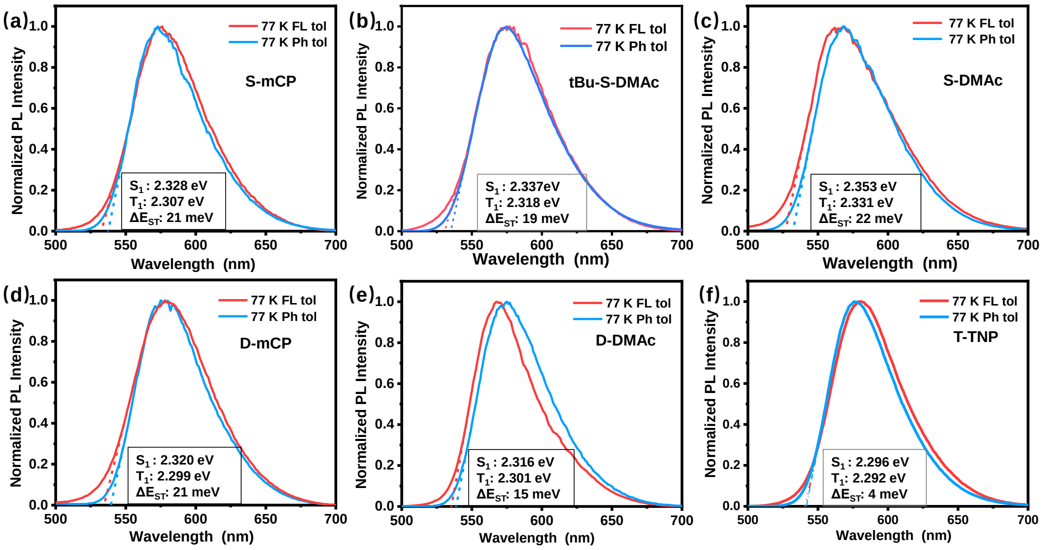

Supplementary Figure 56. Spectroscopy in 77 K. The fluorescence (FL) and phosphorescence (Ph) spectra of the emitters detected in toluene at 77 K for (a) S-mCP; (b) tBu-S-DMAc; (c) S-DMAc; (d) D-mCP; (e) D-DMAc; (f) T-TNP. The energy gap (ΔE_{ST}) between the lowest singlet (S_1) and triplet excited states (T_1) was determined from the difference values of the onset positions of fluorescence and phosphorescence spectra.

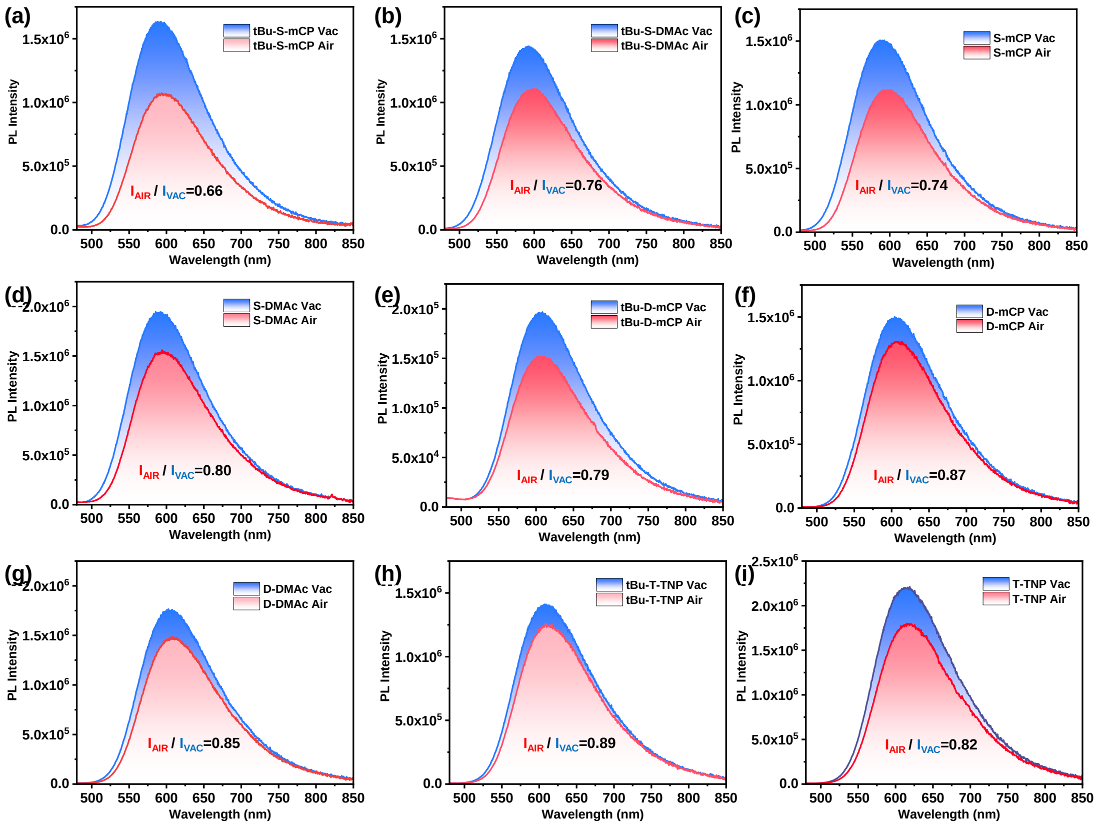

Supplementary Figure 57. Steady-state photoluminescence (PL) spectroscopies in vacuum and air. (a) tBu-S-mCP; (b) tBu-S-DMAc; (c) S-mCP; (d) S-DMAc; (e) tBu-D-mCP; (f) D-mCP; (g) D-DMAc; (h) tBu-T-TNP; (i) T-TNP. The blue and red areas are the steady-state PL spectra in vacuum and in air, respectively. The inset values are the integral ratio of the steady-state spectrum in air to that in vacuum. All the films consist of 2 wt% emitters doped in 5 wt% poly(N-vinylcarbazole) (PVK) and 93 wt% 9H-carbazole-3-carbonitrile (mCP-CN).

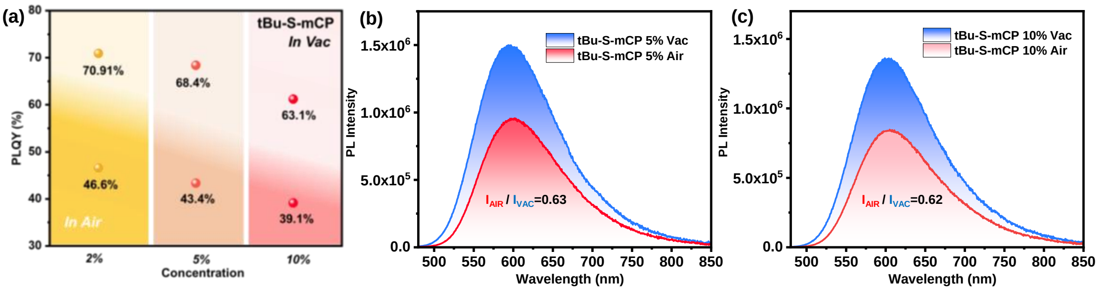

Supplementary figure 58. The Photoluminescence quantum yields (PLQYs) of tBu-S-mCP with the different doping concentrations. (a) The PLQYs of tBu-S-mCP at 2 wt%, 5 wt% and 10 wt% tBu-S-mCP doped in 5 wt% poly(N-vinylcarbazole) (PVK) and corresponding concentrations of 9H-carbazole-3-carbonitrile (mCP-CN) in vacuum (top) and air (bottom). The steady-state spectra in vacuum and air atmospheres of (b) 5 wt% tBu-S-mCP doped films and (c) 10 wt% tBu-S-mCP doped films. The inset values of (b) and (c) are the integral ratio of the steady-state spectrum in air to that in vacuum.

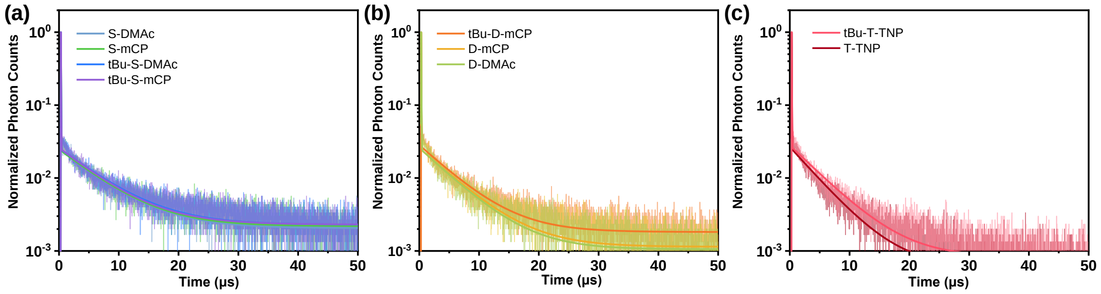

Supplementary Figure 59. The transient photoluminescence spectra of 2 wt% emitters doped in 5 wt% poly(N-vinylcarbazole) (PVK) and 93 wt% of 9H-carbazole-3-carbonitrile (mCP-CN). (a) Single-TADF series (S-DMAc, S-mCP, tBu-S-DMAc, tBu-S-mCP); (b) Double-TADF series (tBu-D-mCP; D-DMAc; D-mCP); (c) Triple-TADF series (tBu-T-TNP, T-TNP). The dotted lines are the measured results, and the solid lines are the fitting results.

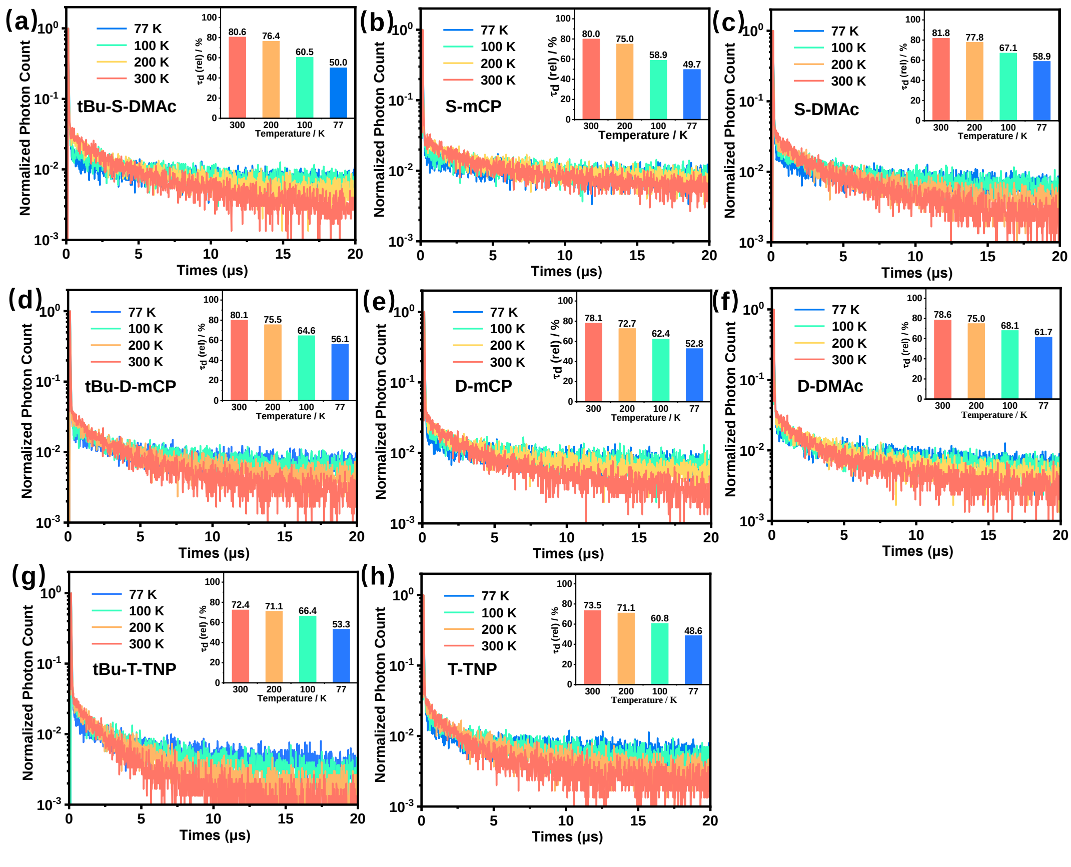

Supplementary Figure 60. The temperature-dependent transient photoluminescence decay spectra. (a) tBu-S-DMAc; (b) S-mCP; (c) S-DMAc; (d) tBu-D-mCP; (e) D-mCP; (f) D-DMAc; (g) tBu-T-TNP; (h) T-TNP. The photoluminescence decays were detected in the blended films from 77 K to 300 K in vacuum. The inset shows the proportion of delayed fractions (τ_d) at different temperatures.

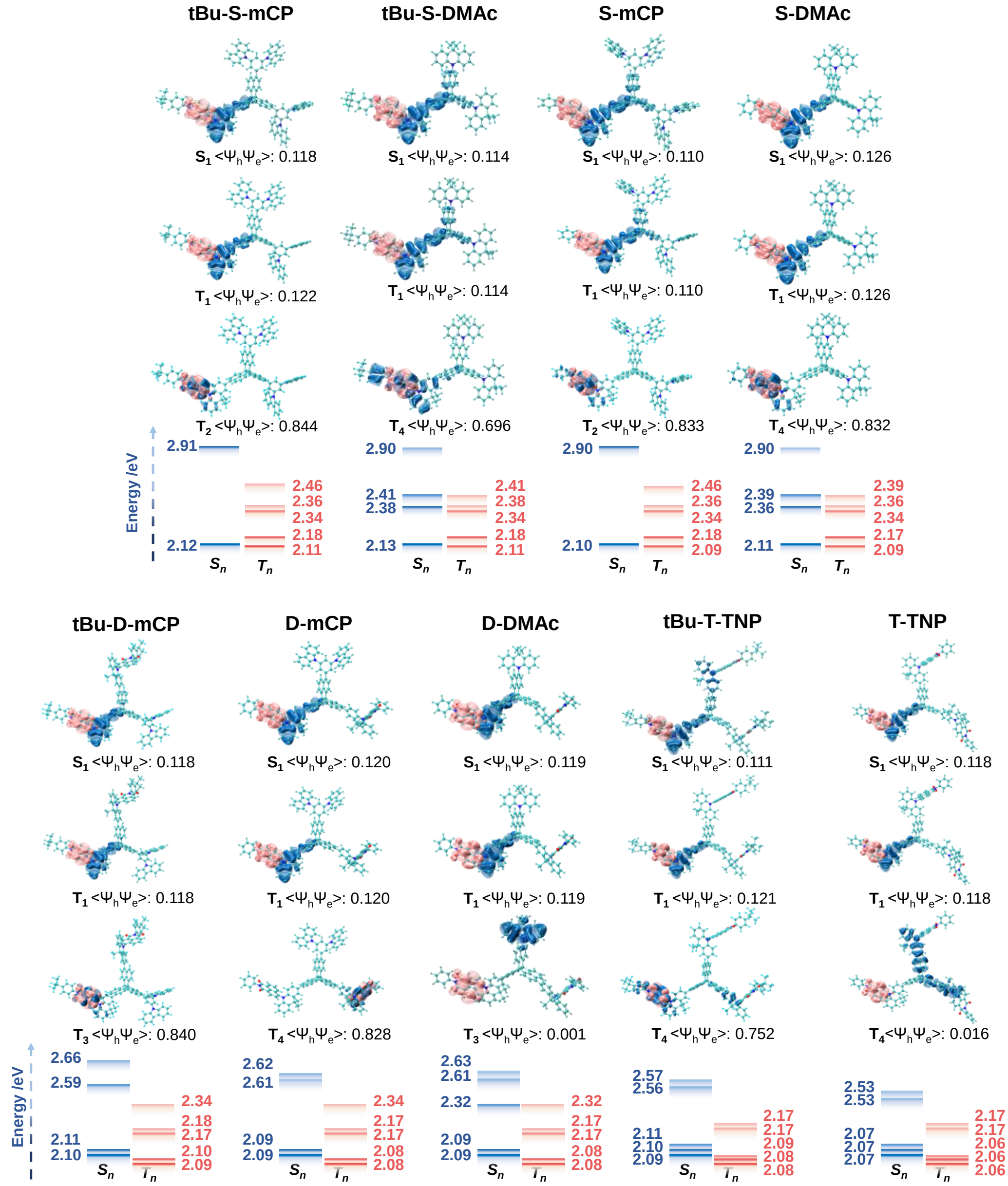

Supplementary Figure 61. Natural transition orbits (NTO). NTO analysis (blue areas are holes and pink areas are electrons), energy levels (S_1 is the lowest singlet excited state, S_n (n=0,1,2,3,4…..) is singlet excited state and T_n (n=0,1,2,3,4…..) is the corresponding triplet excited state), hole–electron overlap integrals (<$\psi_h\psi_e$>) of the emitters.

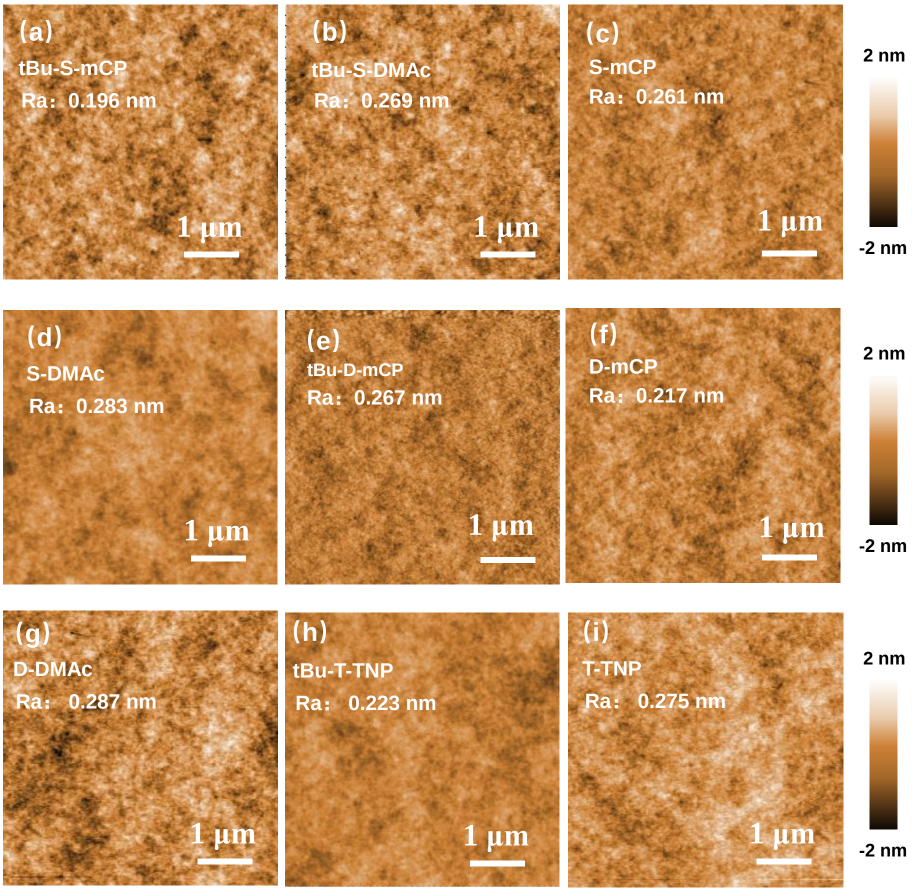

Supplementary Figure 62. Surface roughness of Films. The height images of the blended films detected by atomic force microscopy for (a) tBu-S-mCP; (b) tBu-S-DMAc; (c) S-mCP; (d) S-DMAc; (e) tBu-D-mCP; (f) D-mCP; (g) D-DMAc; (h) tBu-T-TNP; (i) T-TNP. The root mean-square surface roughness (Ra) of the whole scanning area is shown in the images.

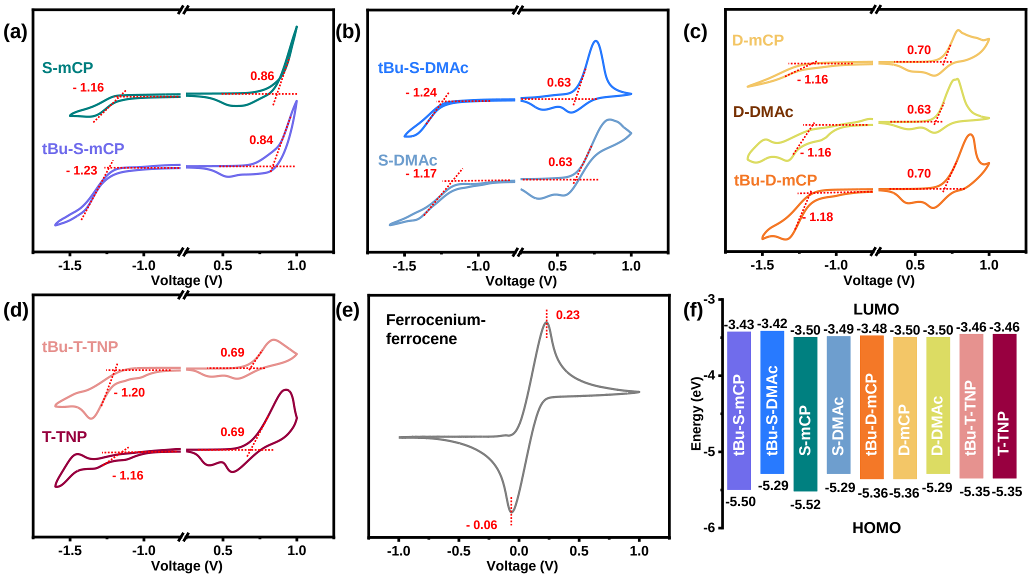

Supplementary Figure 63. Cyclic voltammetry. The Cyclic voltammetry curves of (a) S-mCP and tBu-S-mCP; (b) tBu-S-DMAc and S-DMAc; (c) D-mCP, D-DMAc and tBu-D-mCP;

(d) tBu-T-TNP and T-TNP; (e) the external standard Fc^+/Fc (the peak oxidation and reduction potentials are marked). (f) the corresponding energy levels of highest occupied molecular orbital (HOMO) and lowest unoccupied molecular orbital (LUMO) for nine emitters. The onsets of oxidation and reduction potentials are marked for nine emitters.

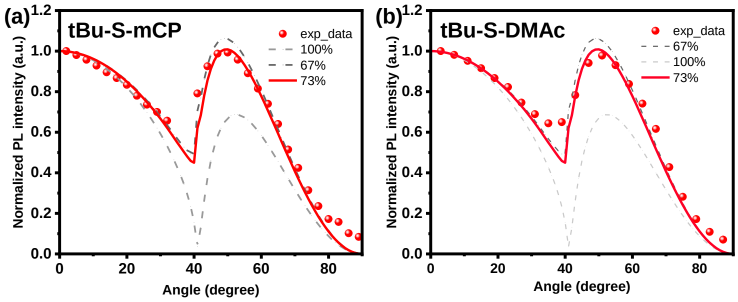

Supplementary Figure 64. Molecular orientation characterization. Angle- and polarization-resolved photoluminescence (PL) spectra of (a) tBu-S-mCP and (b) tBu-S-DMAc (2 wt% doped in 5 wt% poly(N-vinylcarbazole) (PVK) and 93 wt% of 9H-carbazole-3-carbonitrile (mCP-CN)) films. Measured (symbols) and simulated curves (lines and dotted lines) with horizontal dipole ratios ($\Theta_{//}$) are marked.

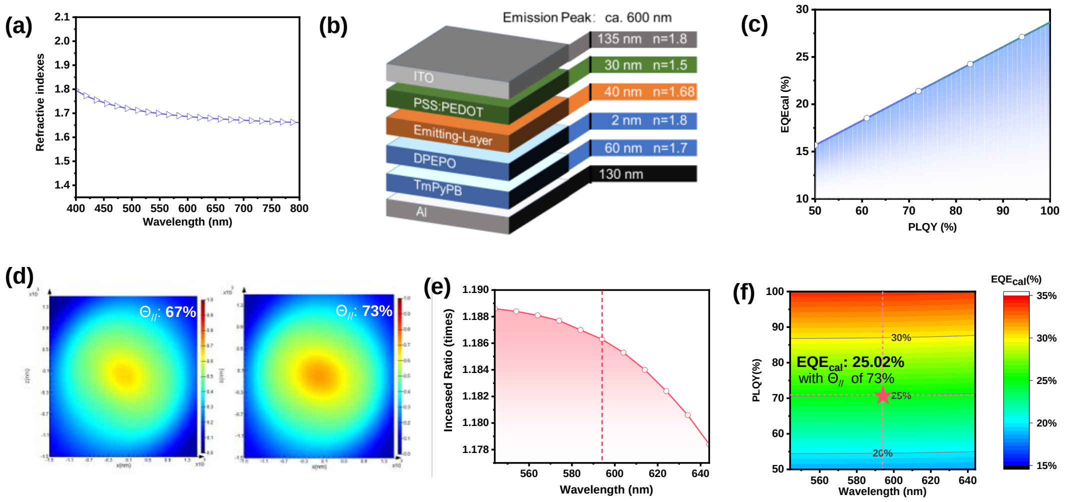

Supplementary Figure 65. Optical simulation. (a) Refractive indexes of tBu-S-mCP doped films measured by variable angle spectroscopic ellipsometry (VASE) (b) Layer structure of the device with the corresponding thickness and refractive indexes (ITO: indium tin oxide; PSS: PEDOT: poly(3,4-ethylenedioxythiophene):poly(styrenesulfonate); PVK: Poly(N-vinylcarbazole); mCP-CN: 9H-carbazole-3-carbonitrile; DPEPO: bis[2-(diphenylphosphino) phenyl] ether oxide; TmPyPB: 1,3,5-tri[(3-pyridyl)-phen-3-yl] benzene; LiF: lithium fluoride; Al: aluminum.). (c) The calculated maximum EQE (EQE_{cal}) according to the photoluminescence quantum yield (PLQY) for device without considering molecular orientation. (d) The front view of luminous dipole emits light to the outside of the substrate with different ratio of horizontal dipole orientation ($\Theta_{//}$: 73% and 67%). (e) The increased ratio of $\Theta_{//}$ 73% versus 67% was calculated using finite difference time domain (FDTD) approach by Lumerical FDTD Solutions 8.7.3 at different wavelengths. (f) The maximum EQE_{cal} according to the PLQY and wavelength for device with ratio of horizontal dipole orientation 73% and the increased ratio of $\Theta_{//}$ 73% versus 67%.

Supplementary Note 5: Supplementary Tables

Supplementary Table 1. Spin-orbit coupling matrix elements (SOCMEs) between the lowest singlet excited state (S_1) and the corresponding triplet excited state (T_n, n=0,1,2,3,4,5) for nine emitters.

λ_{soc} (T_n-S_1) / cm^{-1}	1	2	3	4	5
**tBu-S-mCP**	0.032	0.862	0.158	0.017	0.099
**tBu-S-DMAc**	0.046	0.072	0.086	0.801	0.182
**S-mCP**	0.033	0.869	0.158	0	0.099
**S-DMAc**	0.028	0.067	0.088	0.810	0.167
**tBu-D-mCP**	0.037	0	0.869	0	0.085
**D-mCP**	0.022	0	0	0.837	0.068
**D-DMAc**	0.020	0	0.095	0	0.073
**tBu-T-TNP**	0.028	0	0	0.244	0
**T-TNP**	0.022	0	0	0.044	0

Supplementary Table 2. Summarized performances of solution-processed organic light-emitting diodes (OLEDs) for orange-red small-molecule emitters with thermally activated delayed fluorescence characteristics.

Emitters	λ/ nm[(a)]	V_{on}/ V[(b)]	EQE_{max}[(c)]	CIE(x,y)[(d)]	Ref.
tBu-S-mCP	594	5.4	24.7	(0.54, 0.44)	
tBu-D-mCP	604	5.4	16.4	(0.56, 0.41)	This work
T-TNP	608	5.2	14.9	(0.57, 0.39)	
NAI_R3	622	7	22.5	(0.60, 0.40)	[7]
2SPAC-DBP-2tBuCz	583	2.3	23.7	(0.54, 0.45)	[10]
TS-1	608	5.2	12.58	(0.58, 0.41)	[11]
TAT−DBPZ	604	3.2	15.4	(0.61, 0.38)	[12]
oDTBPZ-DPXZ	612	3.3	18.5	(0.60, 0.40)	[13]
tDBBPZ-DPXZ	620	4.5	10.1	(0.62, 0.37)	[14]
D(DPXZ-Cz)-DCPP	600	2.8	21.6	(0.57, 0.43)	[15]
DDPhCz-DCPP	578	2.8	20.6	(0.48, 0.50)	[16]
PXZPDO	570	5.1	20.1	(0.47, 0.50)	[17]

(a) The peak value of electroluminescence; (b) Turn-on voltage at 1 cd m^{-2}; (c) Maximum external quantum efficiency; (d)Coordinates of Commission Internationale de L'Eclairage.

Supplementary Reference

1. Tao Y. et al. Thermally activated delayed fluorescence materials towards the breakthrough of organoelectronics. *Adv. Mater.* **26**, 7931-7958 (2014).
2. Liu Y. et al. High-efficiency solution-processable OLEDs by employing thermally activated delayed fluorescence emitters with multiple conversion channels of triplet excitons. *Adv. Sci.* **8**, 2101326 (2021).
3. Lu T.& Chen F. Multiwfn: a multifunctional wavefunction analyzer. *J. Comput. Chem.* **33**, 580-592 (2012).
4. Lu T.& Chen F. Calculation of molecular orbital composition. *Acta Chimica Sinica -Chinese Edition-* **69**, 2393 (2011).
5. Xiang H.-Y. et al. Outcoupling-enhanced flexible organic light-emitting diodes on ameliorated plastic substrate with built-in indium–tin-oxide-free transparent electrode. *ACS Nano* **9**, 7553-7562 (2015).
6. Lin H.-W. et al. Pyridine-based electron transporting materials for highly efficient organic solar cells. *J. Mater. Chem. A* **1**, 1770-1777 (2013).
7. Zeng W. et al. Realizing 22.5% external quantum efficiency for solution-processed thermally activated delayed-fluorescence OLEDs with red emission at 622 nm via a synergistic strategy of molecular engineering and host selection. *Adv. Mater.* **31**, 1901404 (2019).
8. Yun J. H.& Lee J. Y. Benzoisoquinoline-1,3-dione acceptor based red thermally activated delayed fluorescent emitters. *Dyes Pigm.* **144**, 212-217 (2017).
9. Zeng W. et al. Achieving nearly 30% external quantum efficiency for orange-red organic light emitting diodes by employing thermally activated delayed fluorescence emitters composed of 1,8-naphthalimide-acridine hybrids. *Adv. Mater.* **30**, 8 (2018).
10. Jiang D. H. et al. Extremely high power efficiency solution-processed orange-red TADF OLEDs via a synergistic strategy of molecular and device engineering. *Adv. Opt. Mater.* **10**, 2102774 (2022).
11. Chen X. et al. Molecular design strategy for orange-red thermally activated delayed fluorescence emitters via intramolecular energy transfer and their application in solution processable organic light-emitting diodes. *Chem. Eng. J.* **428**, 131691 (2022).
12. Liu Y. et al. High-performance solution-processed red thermally activated delayed fluorescence OLEDs employing aggregation-induced emission-active Triazatruxene-based emitters. *ACS Appl. Mater. Interfaces* **12**, 30652-30658 (2020).
13. Chen J. X. et al. Managing locally excited and charge-transfer triplet states to facilitate up-conversion in red TADF emitters that are available for both vacuum- and solution-processes. *Angew. Chem. Int. Ed. Engl.* **60**, 2478-2484 (2021).
14. Chen J. X. et al. Efficient orange-red thermally activated delayed fluorescence emitters feasible for both thermal evaporation and solution process. *ACS Appl. Mater. Interfaces* **11**, 29086-29093 (2019).
15. Zhang Y. X. et al. Solution-processable orange-red thermally activated delayed fluorescence emitters with 3,6-disubstituted carbazole for highly efficient OLEDs with low efficiency roll-off. *J. Mater. Chem. C* **10**, 2034-2041 (2022).
16. Wang B. et al. Highly efficient electroluminescence from evaporation-and solution-processable orange–red thermally activated delayed fluorescence emitters. *J. Mater. Chem. C* **7**, 12321-12327 (2019).
17. Gupta A. K. et al. Thermally activated delayed fluorescence emitters with intramolecular proton transfer for high luminance solution-processed organic light-emitting diodes. *ACS Appl. Mater.*

Interfaces **13**, 15459-15474 (2021).
